# Silicon-photonics optoacoustic sensor for fully wearable blood pressure monitoring

**DOI:** 10.1038/s41467-026-77580-3

**Published:** 2026-09-17

**Authors:** Gil Gelbert, Ron Moisseev, Yoav Hazan, Tamar Harary, Daniel H. Lange, Amir Rosenthal

**Affiliations:** Andrew and Erna Viterbi Faculty of Electrical & Computer Engineering, Technion - Israel Institute of Technology, Haifa, Israel

**Keywords:** Biomedical engineering, Imaging and sensing, Optical sensors, Electrical and electronic engineering

## Abstract

Cuffless blood pressure monitoring is essential for continuous cardiovascular assessment. Current wearable approaches rely on pulse-echo ultrasound patches that track pulsating arterial diameters. However, multi-element designs and precise alignment complicate their applicability as a fully wearable solution. We overcome these challenges by using a highly miniaturized silicon-photonics acoustic detector and optoacoustic excitation to generate ultrasound waves inside the artery. The superior contrast in these optoacoustic signals and the detector’s semi-isotropic sensitivity enable robust artery diameter monitoring with a single detection element, achieving high tolerance to translation and rotation. The detector’s high sensitivity, achieved despite its small size, enables measuring optoacoustic signals from the radial artery with a high contrast at 100 Hz, capturing the features of the blood pressure waveform. Here, we show that the device’s agreement with a reference cuff-based blood pressure monitor highlights the potential of silicon-photonics optoacoustic sensing as a practical solution for continuous, cuffless monitoring in daily life.

## Abstract (web summary)

Continuous cuffless blood pressure monitoring demands wearable sensors insensitive to placement. Here, the authors present a silicon-photonics optoacoustic sensor that accurately tracks arterial diameter and blood pressure using a single miniaturized element, with high tolerance to positioning errors.

## Introduction

Blood pressure (BP) is one of the most informative indicators of cardiovascular function and a critical predictor of morbidity and mortality across a range of chronic conditions. Accurate BP monitoring is essential for diagnosing hypertension, guiding treatment decisions, and evaluating long-term cardiovascular risk. Yet, despite its clinical importance, BP remains difficult to measure continuously and unobtrusively in everyday life. Current methods, which rely on inflatable cuffs or invasive catheters, are either uncomfortable, disruptive, or limited to clinical environments, making them impractical for real-time, long-term monitoring outside of controlled settings. This persistent reliance on bulky or intermittent tools presents a fundamental limitation in how BP data can be collected, analyzed, and ultimately applied to guide clinical or preventive care.

The limitations of clinical BP monitors (Supplementary Notes 1 and 2) have spurred significant interest in the development of cuffless wearable BP monitoring technologies that aim to provide continuous, non-invasive measurement without the need for occlusion or manual operation^1^ (Supplementary Note 3). Many of these approaches estimate BP indirectly using computational models applied on surrogate physiological signals, such as photoplethysmography (PPG)^2–4^, electrocardiography (ECG)^5,6^, or electrical bioimpedance^7^. Early models have used such surrogate signals to extract parameters linked to BP values by physiological models, e.g., pulse transit time^8^ or pulse wave velocity^9^. However, relying on surrogate signals such as PPG has proved to be of limited usability for BP monitoring due to their placement sensitivity (Supplementary Note 4) and need for frequent calibration. With progress in machine learning, the focus has changed to data-driven analysis of a larger set of features exhibited by these signals, often without relying on an underlying physiological model^10^. Despite its promise, the purely data-driven approach falls short of clinical adoption standards owing to overfitting from narrow datasets, sensitivity to physiological variability, and degradation from device placement inconsistencies and environmental interference^11^.

The limitations of surrogate physiological signals highlight the need for a measurement directly linked to blood pressure through a validated physiological model. Arterial diameter offers such a direct correlate, and pulse-echo ultrasound has demonstrated promise in capturing this parameter noninvasively^12–14^. The original implementations have relied on hand-held probes, inappropriate for wearable technology. Significant progress has since been made in developing wearable ultrasound patches capable of real-time imaging, demonstrating 2D^15^ and 3D^16–18^ structural imaging in addition to flow measurements via Doppler ultrasound^19^. Of particular interest for BP measurements, ultrasound patches have shown the capability for monitoring the arterial diameter in the extremities and in the neck, which is used to calculate the BP waveform using a calibrated mechanical model^15,19,20^. In a recent study, wearable ultrasound was clinically validated against a sphygmomanometer in a cohort of healthy patients and against an arterial line for intensive-care patients^21^. In the first cohort, the initial calibration of the mechanical model was found to remain valid up to a year, demonstrating the potential of the artery diameter as a highly reliable proxy of BP.

Despite technological advancements, the nature of pulse-echo ultrasound could limit its applicability for fully wearable BP monitors in smaller arteries, where both the sensor and electronics must be highly miniaturized, and the device must remain insensitive to positioning. This limitation arises because the echoes from the posterior and anterior sides of the artery’s walls are comparable in magnitude to signals from their vicinity (Supplementary Note 5), complicating reliable identification of the arterial boundaries. Consequently, spatial discrimination is required and can be achieved either by fully imaging the artery’s morphology in 3D or by using a focused or collimated acoustic beam that is properly aligned with the artery. Both options are challenging when considering wearable BP monitors: fully wearable 3D ultrasound has only been demonstrated with a sparse 2D, 32-elements array^15^, whereas high-quality 3D images require thousands of elements and sophisticated beamforming, currently achievable only in much bulkier handheld devices^22^. In contrast, a focused or collimated single-element transducer readily facilitates the miniaturization^20^, but requires accurate control of the position and orientation of the transducer, which is difficult to achieve in a wearable device. The most successful design of a wearable ultrasound sensor for BP monitoring relied on a 1D array, where each element effectively generated a semi-collimated acoustic beam, and a desktop ultrasound console was used to separately test which element had the optimal position in which its acoustic beam achieved the optimal overlap with the artery^21^. Thus, 1D arrays still rely on the physical collimation of the acoustic beam, as in single-element transducers, but perform the alignment procedure electronically, rather than mechanically. While this design leads to simpler sensor positioning, its proper operation in small arteries necessitates high element density and a correspondingly large number of elements (Supplementary Note 5). Moreover, 1D arrays are still sensitive to the orientation of the transducer, requiring careful placement of the transducer by a skilled operator.

An alternative approach for visualizing the morphology of blood vessels is optoacoustic (photoacoustic) imaging, where pulsed laser illumination generates acoustic sources inside optically absorbing structures of the tissue^23,24^. Since blood is the most absorbing tissue constituent in the visible and near-infrared range, optoacoustic imaging achieves a superior contrast between blood vessels and the surrounding tissue in comparison to pulse-echo ultrasound, which may enable simpler detection configurations. However, optoacoustic systems conventionally rely on piezoelectric ultrasound transducers to detect the acoustic signals, leading to imaging devices that resemble those of pulse-echo ultrasound in their form factor and data acquisition architecture^25^. One of the challenges in developing wearable optoacoustic systems is that the optoacoustic signal is most often significantly weaker than pulse-echo signals, imposing further tradeoffs in the design. For example, sufficient signal-to-noise (SNR) has so far been achieved by optoacoustic patch technologies by reducing the frame rate to the range of 1 Hz^26^, 10 Hz^27^, and 25Hz^28^ (Supplementary Note 6 and Supplementary Table 2), which is insufficient for capturing the BP waveform. In addition, current optoacoustic patches rely on piezoelectric arrays, thus inheriting the same technological complexities and positioning sensitivity that have constrained pulse-echo ultrasound’s potential as a wearable BP estimation technology.

In this work, we develop a paradigm for cuffless BP monitoring that overcomes the inherent tradeoff between complexity and robustness of previous devices^11^ by using a single-element optoacoustic sensor that does not require high accuracy in its position and orientation to measure the diameter of superficial arteries. The optoacoustic sensor is comprised of a fiber bundle delivering the optoacoustic illumination, and a silicon-photonics acoustic detector (SPADE^29,30^) for capturing the resulting ultrasound signals (Methods). SPADE offers a unique combination of properties that overcome the tradeoffs found in piezoelectric technology, which limits the latter’s suitability for BP monitoring. While the sensitivity of piezoelectric detectors scales with area, SPADE achieves both point-like detection and sufficiently high sensitivity, enabling real-time, averaging-free measurements—crucial for capturing the rapid dynamics of the BP waveform. In addition, SPADE operates with a wide reception bandwidth of DC −125 MHz, which theoretically corresponds to an axial resolution of 11 µm^31^. In practice, this bandwidth far exceeds the maximum signal bandwidth from the superficial radial artery, and the axial resolution is determined by optoacoustic pulse duration, the artery’s geometry, and the acoustic properties of the tissue. The optoacoustic sensor is tested on the distal forearm of healthy volunteers, and its results correlate with a direct measurement of the BP waveform, achieving accuracy within the range associated with high-grade device performance (Supplementary Note 3 and Supplementary Table 1). In addition, our phantom study shows that the diameter estimation achieved by the optoacoustic sensor for cylindrical objects remains consistent over a range of translation and rotation values that may occur in the clinical application due to sensor misplacement or artery orientation. The simplicity and robustness of this methodology—combining the instrumental minimalism of single-element detection with insensitivity to precise positioning—highlights its potential as wearable technology for accurate, unobtrusive BP monitoring.

## Results

### Device design

**Fig. 1:**
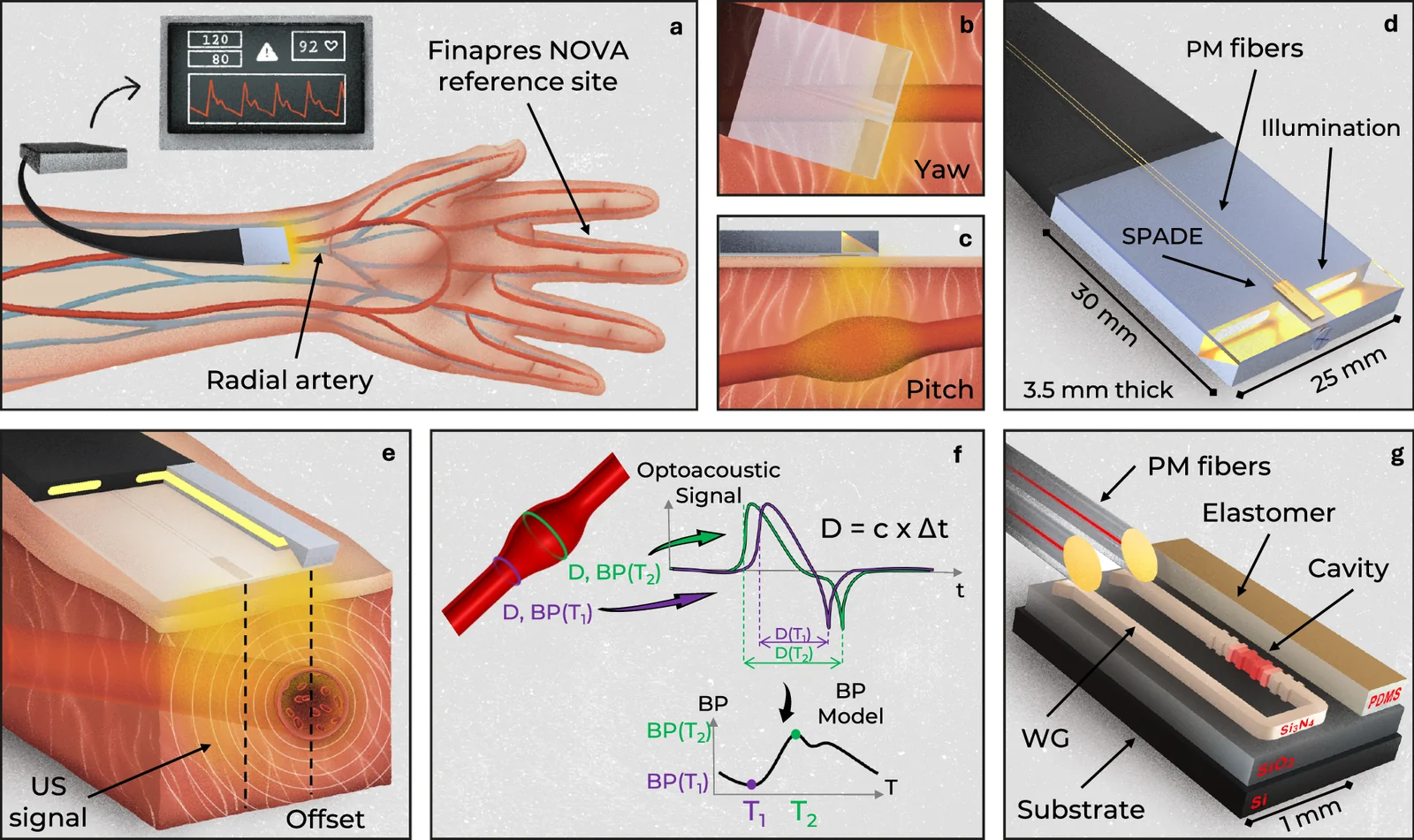
Optoacoustic blood pressure estimation. **a** A wearable optoacoustic sensor targeting the radial artery close to the wrist. The sensor is connected to a console that reads out the optoacoustic signals and converts them into the BP (blood pressure) waveforms. The results are compared to a reference system on the finger. **b**,** c** The sensor is positioned on the skin above the radial artery with an unknown yaw angle, which depends mainly on the placement, and an unknown pitch, which depends mostly on the vertical orientation of the artery itself. **d** The operational end of the sensor, showing the illumination pattern and the SPADE (silicon photonics acoustic detector), which is coupled with two PM (polarization-maintaining) optical fibers. **e** The optoacoustic sensing procedure involves an excitation light from the sensor that diffuses in the tissue and induces outward-propagating US (ultrasound) waves inside the artery that reach the SPADE. **f** During each cardiac cycle, a pressure pulse travels down the arterial tree, altering the arterial diameter. The optoacoustic signal is used to directly measure the arterial diameter, and a physiological algorithm is used to convert it to a BP value. **g** The structure of the SPADE is shown, where the pressure-sensitive volume is the section of the WG (waveguide) marked in red.

The optoacoustic sensor, illustrated in Fig. 1a, measures the diameter of the radial artery and can operate even when positioned with a spatial or angular offset relative to the artery (Fig. 1b, c). The sensor performs two operations on the surface of the tissue: optical illumination, delivered by a fiber bundle, and ultrasound detection performed with SPADE (Fig. 1d). The illumination source (Methods) is a pulsed laser operating at the wavelength of 800 nm, which achieves deep-tissue penetration that reaches the arteries in the forearm, as illustrated in Fig. 1e, and at a repetition rate of 100 Hz, sufficient for sampling the BP waveform. Because of the high optical absorption of blood, more energy is deposited in the artery than in the surrounding tissue, leading to a rapid temperature and pressure increase inside the artery with respect to its surroundings, ultimately resulting in an outward-propagating acoustic wave via the optoacoustic effect (Fig. 1e). This time-resolved acoustic wave, referred to as the *optoacoustic signal*, is governed by the time-of-flight principle, which enables determining the depth of structures based on their delays. Accordingly, the arterial diameter is encoded in the optoacoustic signal and is monitored as the artery expands and contracts during each cardiac cycle (Fig. 1f). Using Hayashi’s empirical model^32^, previously used in ultrasound-based BP monitors^20,21^, the recorded arterial diameter waveform is transformed into the BP waveform (Methods).

The illumination is delivered to the tissue by a flexible ribbon-shaped fiber bundle that was split into two segments at its distal end, leading to two adjacent beams, 10.5 × 5 mm each, and 4 mm apart (Fig. 1d). Because of the broad illumination, the radial artery was properly illuminated even when the center of the sensor was at an offset from the artery (Fig. 1e). To achieve this wide tissue illumination while keeping the bundle thin (3 mm), borosilicate fibers with a high numerical aperture (NA = 0.55) were used together with a slanted mirror (Methods). The SPADE was positioned on the metal partition between the two beams and was connected to its interferometric read-out system by two polarization-maintaining (PM) fibers. Figure 1g shows the internal structure of the SPADE, which includes fiber-to-chip grating couplers to direct light in and out of the chip, and the sensing element: a 1D optical cavity in a silicon nitride core cladded by a transparent elastomer to enhance sensitivity and improve signal fidelity (Methods). The readout system operates by monitoring the pressure-induced refractive-index modulation in the elastomer cladding due to the optoacoustic signal (Methods).

### Properties of the optoacoustic signal

**Fig. 2:**
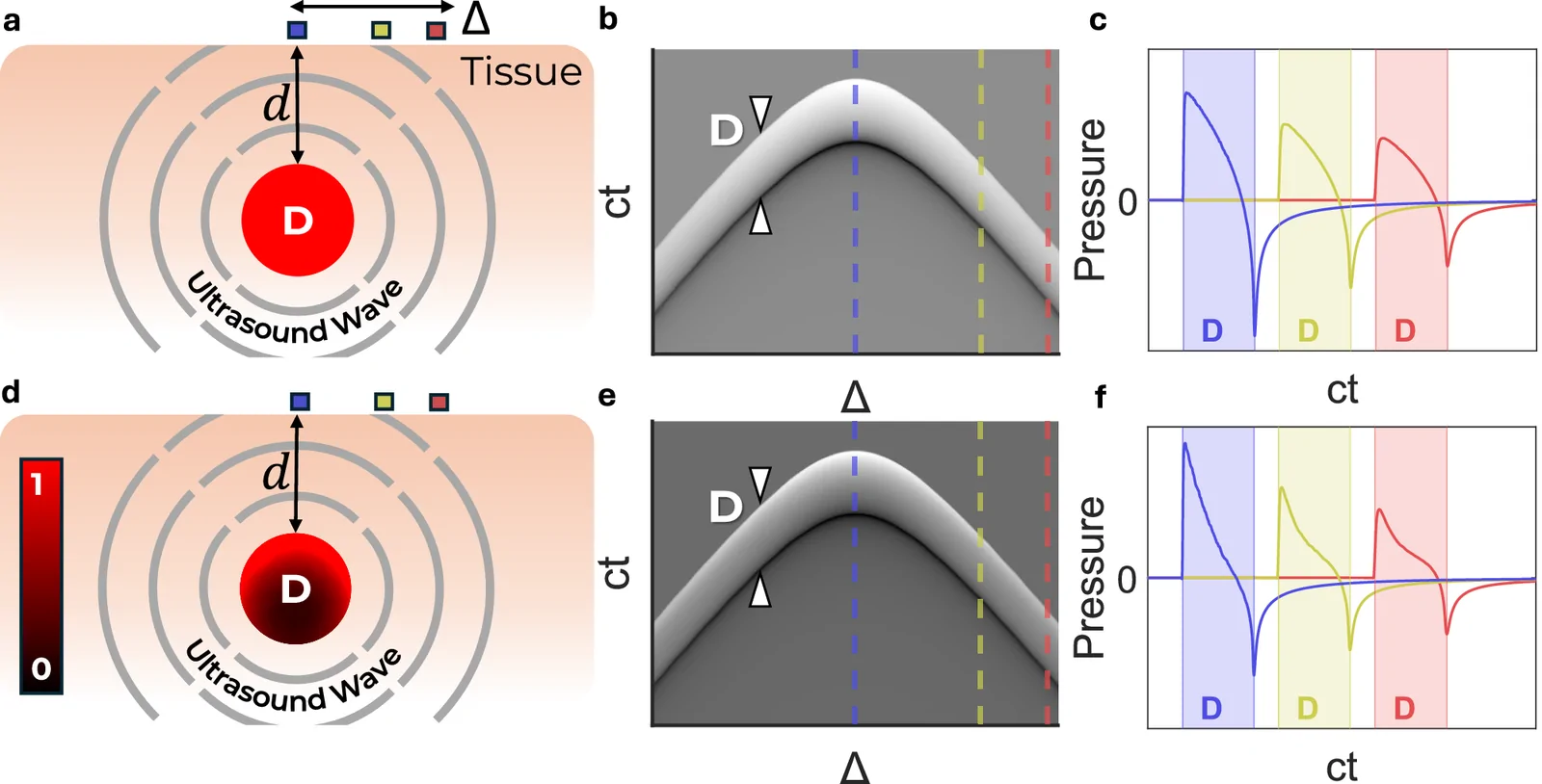
Properties of the optoacoustic signal. **a** Idealized illustration of an isolated artery, represented by a uniform optoacoustic source, with an acoustic detector positioned at an offset Δ. **b** An image representing the acoustic signal as a function of normalized time, ${ct},$ and sensor offset Δ. Regardless of the offset, the width of the signals remains D. **c** Selected 1D pressure waveforms at several positions, showing that D is coded in the signal discontinuities. **d** A realistic illustration of an isolated artery, represented by a cylindrical source with decaying magnitude due to light attenuation in tissue. **e**,** f** The corresponding acoustic signals, presented in the same format, demonstrating that D remains coded in the signal’s discontinuities.

Optoacoustic theory enables the calculation of the acoustic pressure generated by the optical pulse excitation by solving a wave equation for a source representing the energy deposition map in the tissue (Supplementary Note 7). Assuming that the optoacoustic signal is dominated by a single artery, one may use a simplified model in which the optoacoustic source has a cylindrical boundary. When the source is uniform (homogenous) and the detector is infinitesimal, an isotropic analytical solution exists that depends only on the distance of the detector from the cylinder (Supplementary Note 7). For a cylinder with a diameter of D, this analytical solution contains two discontinuities with a temporal separation of D/c, where c is the blood speed of sound. Geometrically, the two discontinuities in the signal code the distances of the detector from the anterior and posterior ends of the cylinder via the time-of-flight principle (Supplementary Fig. 9). Adopting this solution to the typical^33–35^ geometry of the radial artery in the forearm, in which an artery with a diameter $D=2\,{\rm{mm}}$ lies at a depth $d=4\,{\rm{mm}}$ below the tissue surface, and the detector is positioned on the surface with an offset Δ from the artery (Fig. 2a), one can plot $p\left(t,\,\Delta \right)$ (Fig. 2b, Supplementary Note 8), which captures how the optoacoustic waveform changes with Δ. As Fig. 2b illustrates, the delay between the signal discontinuities maintains a constant value of D/c, independent of the offset Δ. Additionally, the optoacoustic signal generally maintains the same shape, where the effect of Δ is limited to the delay and amplitude of the signal (Fig. 2c).

In practice, the optoacoustic source is not homogeneous since it represents the density of the absorbed energy in the tissue, and therefore it decays with depth in proportion to the decay of the illumination intensity. Specifically, the optoacoustic source is proportional to the product of the absorption coefficient, which is an inherent property of the tissue and is indeed approximately uniform within the artery, and the light fluence, which may be calculated when the illumination pattern and tissue parameters are known. Figure 2d shows the simulated optoacoustic source for the same cylindrical geometry of Fig. 2a, but with the addition of the contribution of the fluence decay, calculated for typical tissue parameters (Supplementary Fig. 11). As the figure shows, the artery is no longer a uniform optoacoustic source, and deeper regions of the artery correspond to lower optoacoustic source magnitude due to light attenuation. Nonetheless, the source maintains a cylindrical boundary, which is still coded in the discontinuities of the optoacoustic signals via the time-of-flight principle (Supplementary Fig. 9), as shown by the simulated optoacoustic signals in this case (Fig. 2e, f).

### Device characterization

**Fig. 3:**
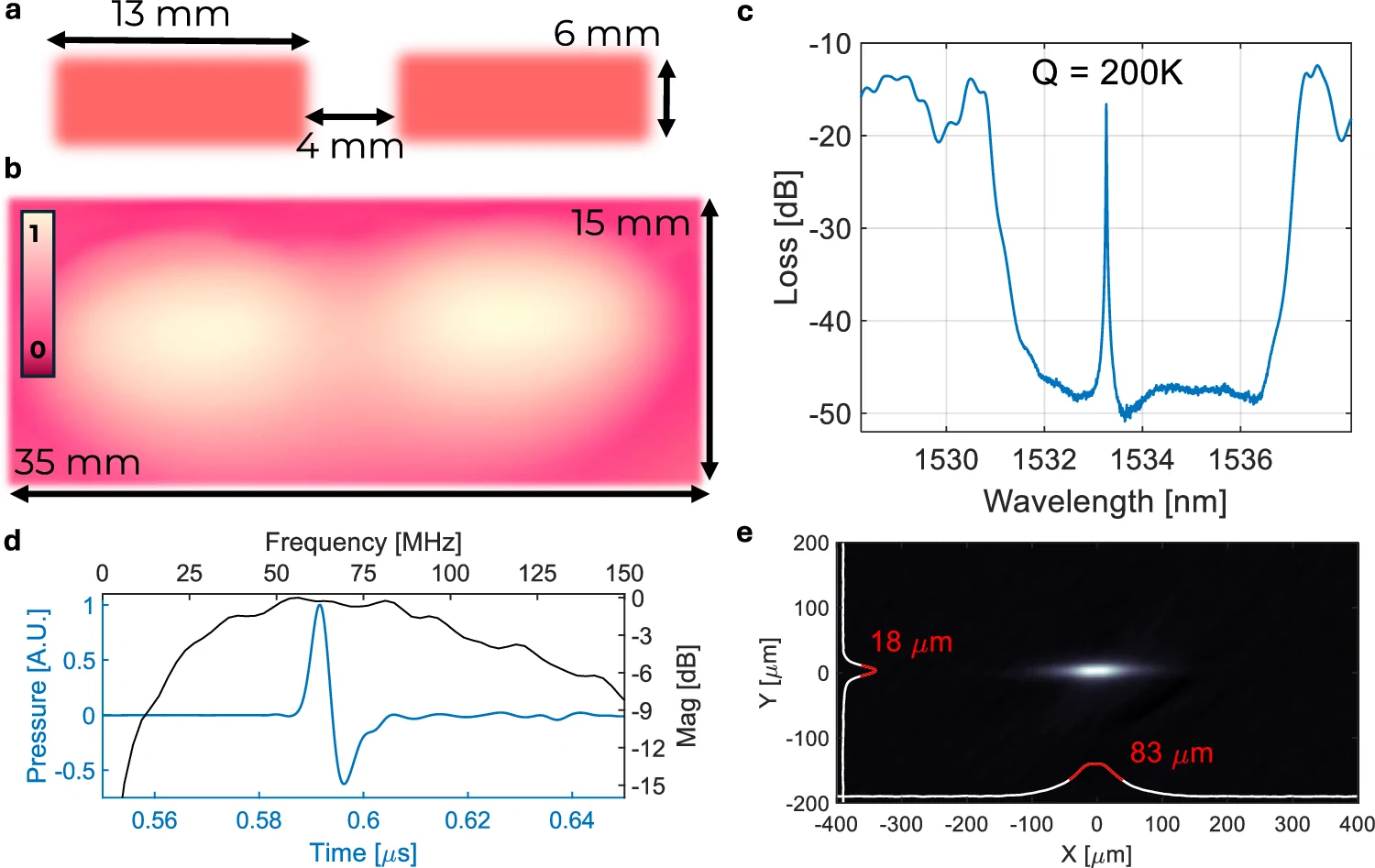
Device characterization. **a** The dimensions of the beam exiting the device at the contact point with the skin. **b** A photograph of the beam at a depth of 4 mm in an optically scattering medium that mimics the tissue, revealing that the two illumination spots are combined due to light diffusion. **c** The optical spectrum of the SPADE (silicon photonics acoustic detector) resonator showing a Lorentzian resonance with a Q factor of 2 × 10^5^. **d** The acoustic impulse response and spectrum of the SPADE, showing a reception bandwidth of 125 MHz at −6 dB. **e** The measured PSF (point spread function) of the SPADE, revealing its effective width and length.

The illumination profile on the surface of the device is shown in Fig. 3a, revealing two spots with dimensions of 13 × 6 mm each and a gap of approximately 4 mm. The width expansion is due to the beams propagating in the glass slide, and the length expansion is due to the beam extending to the sides, beyond the footprint of the device. The fluence and average intensity were 3.14 mJ cm^−2^ and 314 mW cm^−2^, within the ANSI safety limit (Supplementary Note 9). The illumination profiles at typical depths for the radial artery were simulated numerically (Supplementary Fig. 12), accounting for the optical properties of the skin (Supplementary Note 8). The simulated fluence at the anterior wall depth range (3−5 mm) spans 0.8−1.3 mJ cm^−2^, and at the posterior wall depth range (4−7 mm) spans 0.5−1.0 mJ cm^−2^, indicating that the posterior wall receives no less than 50% of the anterior wall fluence across all expected arterial depths, sizes, and sensor offsets.

The illumination profile at a typical depth was also measured using a 4 mm thick tissue-mimicking agar slab placed on the surface of the sensor. As shown in Fig. 3b, light scattering in the slab merges the two illumination beams into a single beam with approximate dimensions of 15 × 35 mm, indicating that the optoacoustic illumination can reach the artery even when the sensor is at an offset of ±10 mm. This experimental result agrees with the simulation performed at the same depth.

The optical transmission spectrum of the resonator used in the SPADE is presented in Fig. 3c, showing a Q-factor of 2 × 10^5^ and fiber-to-fiber insertion loss of 12 dB. The acoustic characterization of the SPADE was performed using the methodology of Ref. ^36^ and is described in Supplementary Note 10 and Supplementary Fig. 13. The acoustic characterization reveals a noise-equivalent pressure (NEP) of 2 mPa Hz^−1/2^, slightly better than the original design of Ref. ^30^. The acoustic impulse response and bandwidth of SPADE are presented in Fig. 3d, demonstrating a reception bandwidth of 125 MHz at −6 dB, corresponding to a theoretical axial resolution of 11 µm^31^. The measurement of the intrinsic bandwidth of SPADE was performed with a designated optoacoustic characterization source with a pulse duration of 1 ns (Supplementary Note 10). The maximum bandwidth of the BP sensor was reduced to 63 MHz due to the longer pulse duration of its optoacoustic source (7 ns, Methods), corresponding to a maximum resolution of 22 µm. Although the physical length of the Bragg grating was 0.5 mm, its effective length was shorter due to light localization in the resonance mode. To experimentally verify this result, the acoustic point spread function (PSF) of the SPADE was measured by performing a tomographic reconstruction of an optoacoustic point source (Supplementary Note 10). Figure 3e shows the measured PSF, revealing an effective sensing length of 83 µm. We note that the width of the PSF, 18 µm, does not represent the effective width of the detector (1 µm), but rather the resolution limitations of the PSF measurement. Accordingly, the SPADE may be geometrically modeled by a line segment with infinitesimal width and height*, i.e*., considerably smaller than the acoustic wavelength, and a length of 83 µm. Moreover, for acoustic wavelengths larger than 166 µm the SPADE may be considered as point-like.

### Robust measurement of the radial artery diameter

A key advantage of the optoacoustic sensor is its ability to measure the arterial diameter without requiring precise control of its position or orientation. Indeed, this ability is inherent to optoacoustic measurements of objects with cylindrical boundaries when they are performed with an infinitesimally small detector (Fig. 2). Although the SPADE used in this work is not infinitesimal (Fig. 3e), we show experimentally that it is sufficiently small to produce consistent measurements of the radial artery in the forearm across a range of sensor offsets and orientations. The first set of measurements was performed on phantoms that mimicked the optical and acoustic properties of the radial artery in the forearm and its surrounding tissue, as well as its geometry (Supplementary Note 11). The diameter of the emulated artery was measured for offsets (Fig. 1e) up to ±10 mm, yaw angles (Fig. 1b) of up to 30°, and pitch angles (Fig. 1c) up to 10°, where the offset and yaw values represented possible misplacement of the sensor, and the pitch represented the variation in the artery’s vertical orientation expected in the wrist region (Supplementary Fig. 16). The results show that the sensor’s orientation did not have significant effect on the accuracy of the diameter measurement, with typical standard deviation (STD) values of approximately 10 µm obtained for all the orientations (Supplementary Fig. 17). We note that this diameter uncertainty, which may be partially explained by the imperfect fabrication of the phantom, translates to a BP estimation STD of only 3.3 mmHg (Supplementary Note 12). In contrast, when the phantom measurement was repeated using a single element from the piezoelectric optoacoustic probe (Supplementary Note 13 and Supplementary Table 4), the diameter could be estimated from the optoacoustic signals only for a smaller span of ±5 mm. In addition, the STD values for the diameter estimations were highly dependent on the orientation of the sensor, where even a modest yaw angle of 5° led to signal distortion that obfuscated the discontinuities in the signal (Supplementary Fig. 21). Numerically, even in the case of perfect orientation the piezoelectric probe achieved a larger STD of 20 µm, and in the case of large yaw angles ( > 10°) STD values could exceed 100 µm (Supplementary Fig. 22).

The second set of measurements was performed on the radial arteries of a volunteer (29-year-old male) close to the wrist, where the control over the position and orientation of the optoacoustic sensor was performed manually. The wrist was palpated to locate a superficial point of the pulsatile radial artery, establishing the initial position for the probe placement. The probe was coupled to the skin with US gel and oriented relatively parallel to the arm at this initial position. The position was then adjusted to achieve minimum delay for the pulsating optoacoustic signal, indicating the optimal positioning in which the SPADE is closest to the artery. This initial positioning maneuver typically requires less than 10 seconds. While readings were being captured, sub-optimal positions were explored by translating the probe across the arm to create a lateral offset with an approximate range of ±5 mm and rotating the probe to increase the yaw up to an angle of 30°.

**Fig. 4:**
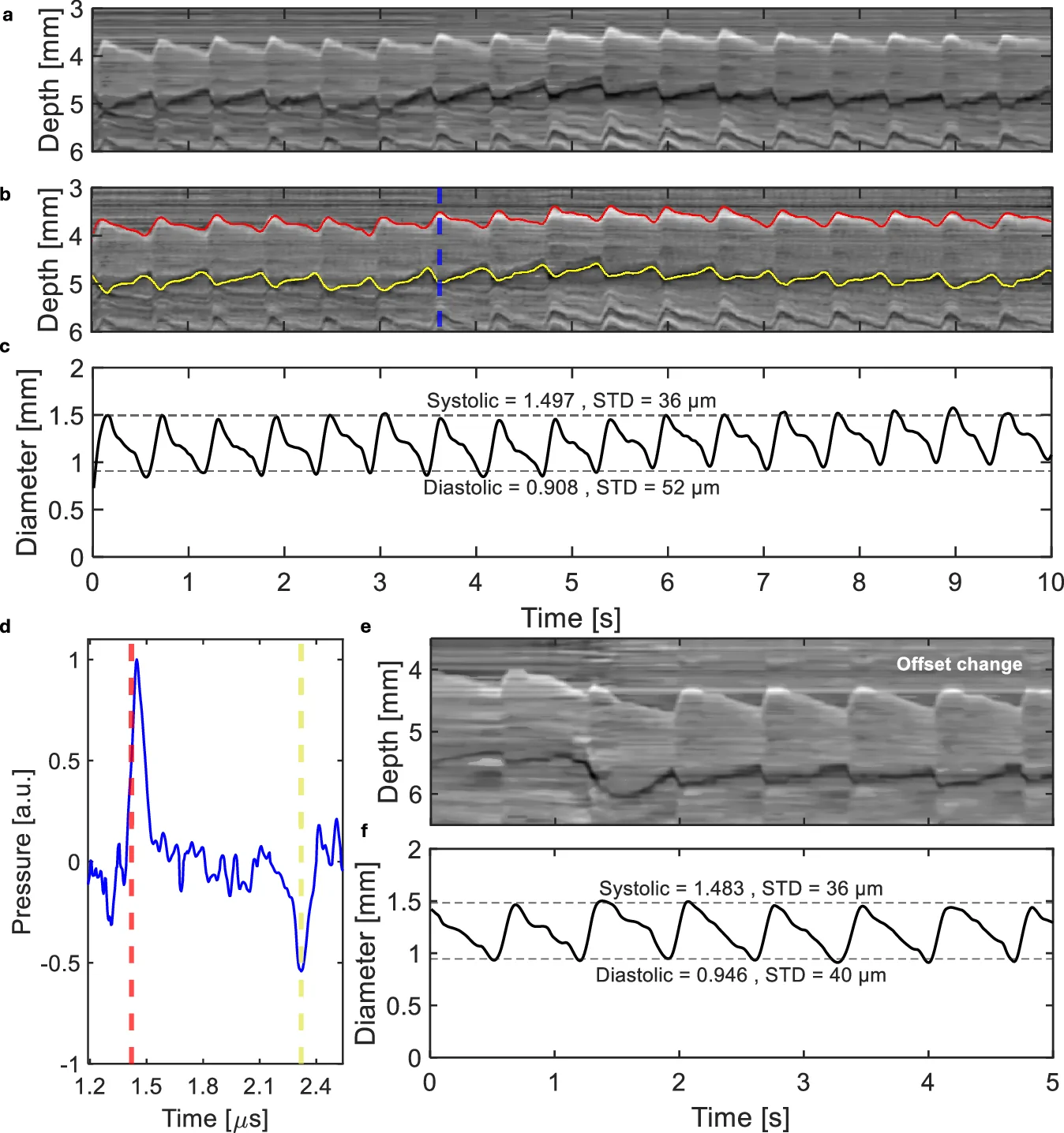
In vivo measurement of arterial diameter. **a** In vivo M-Mode image of SPADE (silicon photonics acoustic detector) signals obtained from the radial artery of a 29-year-old male close to the wrist, and **b** the same M-Mode image with overlaid envelopes. **c** In vivo diameter waveform over time with the average and STD (standard deviation) of the systolic and diastolic levels. **d** A representative 1D pressure signal with marked anterior and posterior positions as they were found. **e** M-Mode recording taken while manually sliding the optoacoustic sensor on the skin, demonstrating that continuous operation is maintained even under motion. The increase in the distance of the artery from the artery is clearly seen in the larger effective depth of the radial signal. **f** The resulting diameter waveform during the motion of the optoacoustic sensor, showing that no distortion or increased estimation STD were observed due to the motion.

Figure 4a shows a 10 s M-mode image of the optoacoustic signal after conditioning (Methods) for the optimal position and orientation, and Fig. 4b shows the same image, but with the signal discontinuities highlighted (Methods), which signify the artery boundaries. The corresponding arterial diameter is presented in Fig. 4c, showing that the diastolic and systolic values remained relatively stable with average values of 0.9 and 1.49 mm with STDs of 52 and 36 µm, respectively. This baseline STD is explained by normal beat-to-beat variation in the BP^37^. Figure 4d shows a representative 1D optoacoustic waveform from the measurement, revealing an excellent signal-to-background ratio of 20 dB, where the intrinsic SPADE noise was approximately half the background signal. In addition, the Figure shows that the leading (positive) and trailing (negative) edges of the arterial signal possessed comparable magnitude. As quantified in Supplementary Fig. 23a, the median anterior-to-posterior signal ratio was 0.96, with 95% of measurements falling between 0.46 and 2.33. Figure 4e shows the first 5 s of an M-mode image and the corresponding diameter signal (Fig. 4f) captured during a manual translation of the optoacoustic sensor on the surface of the arm. As the figure shows, the optoacoustic sensor operated continuously during the measurement, capturing changes in the delay of the optoacoustic signals due to the sensor’s translation. The STDs of the diastolic and systolic diameter estimations during the translation were 40 and 36 µm (calculated over 10 seconds), comparable to the value obtained in Fig. 4c for the optimal sensor position. Therefore, these STD values may also be explained by normal variations in BP over short durations.

In contrast to the high translational robustness of our SPADE-based optoacoustic sensor, measurements performed using a single piezoelectric element, operating in either optoacoustic or pulse-echo mode, exhibited pronounced position sensitivity. For the piezoelectric-based optoacoustic probe (Supplementary Note 13 and Supplementary Fig. 24), the estimated arterial diameters varied substantially over a ± 3 mm offset span, with standard deviations of 125 and 115 µm for the diastolic and systolic phases, respectively. Notably, even within the diameter waveforms recorded at a single offset, the diameter standard deviations exceeded 80 µm, likely due to the reduced signal quality at these positions. The pulse-echo probe (Supplementary Note 5 and Supplementary Fig. 8) demonstrated even greater sensitivity to spatial offsets, where a displacement as small as 0.3 mm resulted in nearly a 200 µm error in the diameter estimation.

**Fig. 5:**
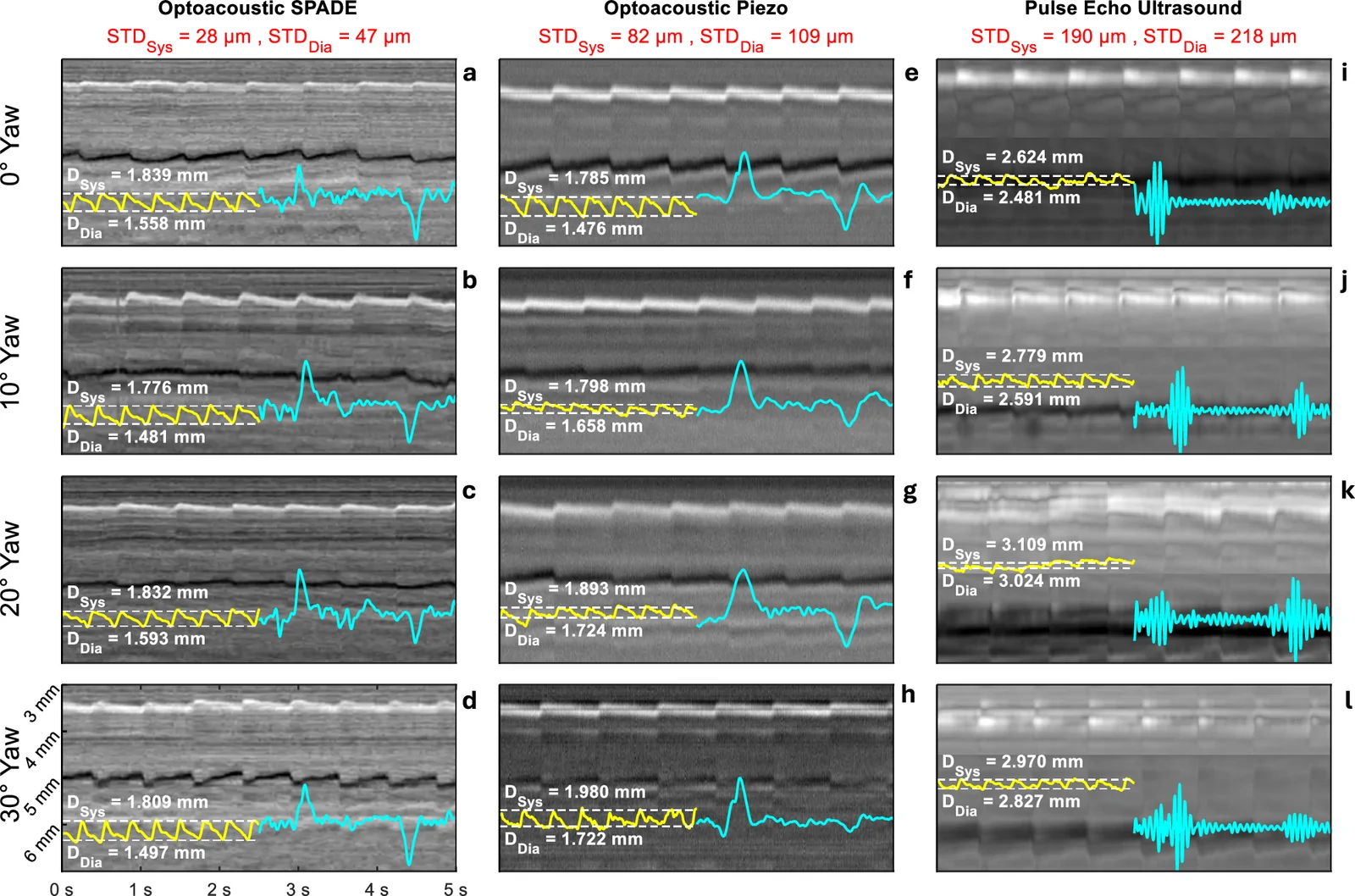
In vivo modalities comparison. M-mode images and diameter estimations of the radial artery of a 29-year-old male close to the wrist for yaw angles ranging 0°−30°, obtained using (**a**–**d**) our SPADE (silicon photonics acoustic detector) based optoacoustic sensor, a single piezoelectric element centered on the artery in an (**e**–**h**) optoacoustic measurement and in a (**i**–**l**) pulse-echo measurement (with the posterior end inverted, to be visually comparable to optoacoustic signals). The blue traces show typical optoacoustic/ultrasound signals, and the yellow traces show the estimated diameter waveform.

Figure 5a–d present the M-mode images acquired with our optoacoustic sensor for yaw angles of 0°−30°, together with the corresponding arterial diameter traces shown in yellow. The STDs of the diastolic and systolic diameters across the four measurements were 47 and 28 µm, respectively, consistent with the stationary measurement results shown in Fig. 4c. The similar waveform morphology observed in all four M-mode images confirms that rotation did not introduce noticeable distortions into the optoacoustic signal. In contrast, when a single element of a piezoelectric optoacoustic probe was used (Fig. 5e–h), which was centered on the artery, yaw rotation caused noticeable signal distortions, with substantially higher STDs of 109 µm (diastolic) and 82 µm (systolic). Even greater estimation variability and signal distortion were observed in pulse-echo ultrasound images (Fig. 5i–l), obtained by a single piezoelectric element (Supplementary Note 5), yielding STDs of 218 and 190 µm for the diastolic and systolic diameters, respectively. To facilitate visual comparison with the optoacoustic data, the pulse-echo M-mode images were post-processed as described in Supplementary Note 5. The significant distortions in the ultrasound images may be explained by echoes from surrounding tissue, whose relative contribution increases with rotation as the acoustic beam loses its alignment with the artery. In contrast, the optoacoustic M-mode images are dominated by the signal from the artery at all yaw angles, resulting in more consistent images. Nonetheless, when the sensing element was not centered on the artery, both the optoacoustic and ultrasound signals from the piezoelectric probes experienced more significant distortions (Supplementary Fig. 25). Finally, a systemic offset was observed between the two modalities—diameter estimates obtained from pulse-echo ultrasound were up to 1 mm larger than those from optoacoustics. This difference arises because optoacoustic sensing measures the inner arterial diameter, whereas ultrasound measurements include contributions from the arterial walls.

### Blood pressure monitoring and validation

**Fig. 6:**
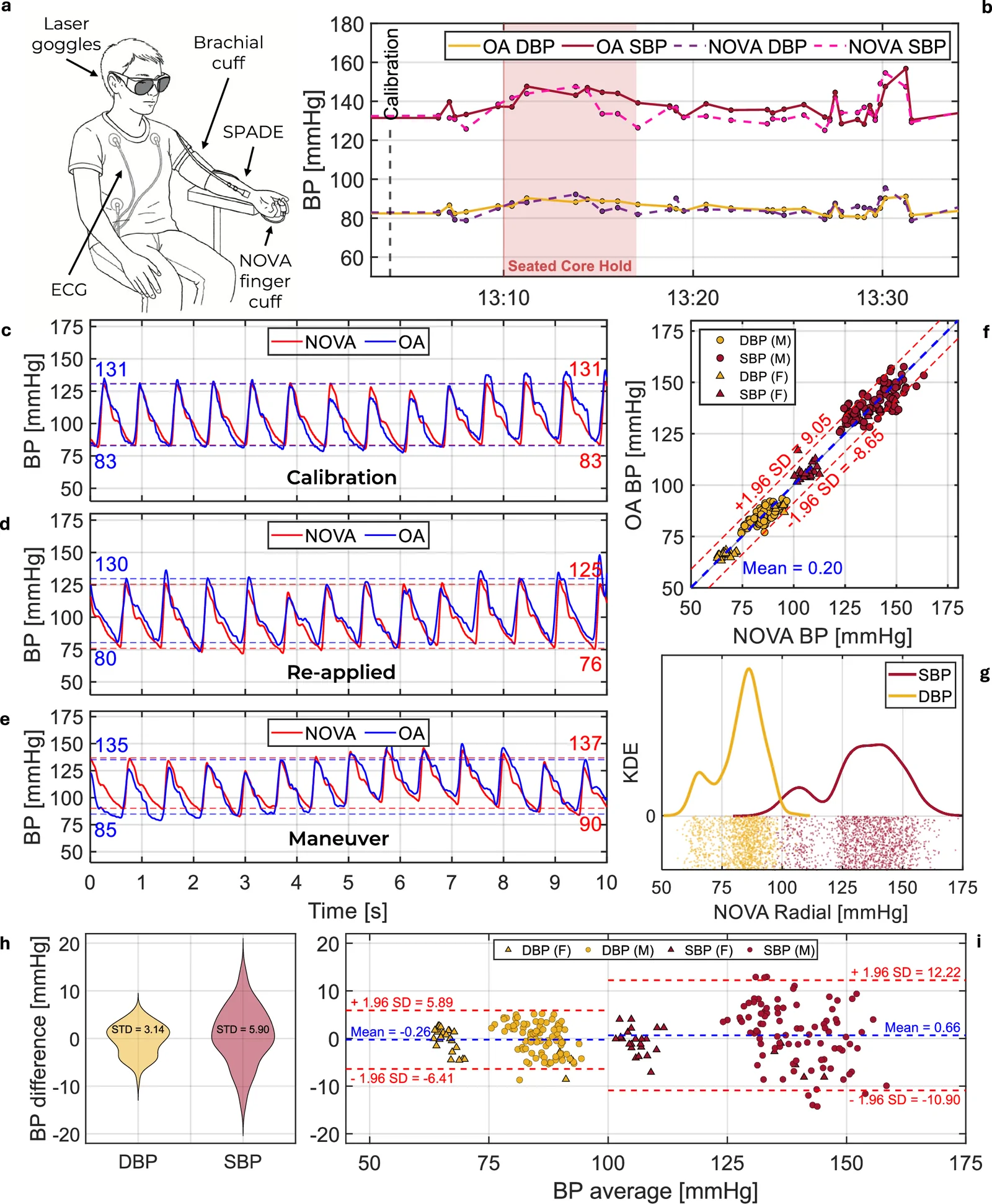
BP monitoring and validation. **a** An illustration of the test subject during the measurement. The left hand is supported at heart’s height. **b** The SBP (systolic blood pressure) and DBP (diastolic blood pressure) readings of the reference (NOVA) and optoacoustic (OA) sensor are plotted together for a continuous measurement with a duration of over 30 min, where the calibration was performed several days earlier (on the male volunteer). The seated-core-hold position is assumed, showing elevated blood pressure values for both measurement techniques. **c**–**e** The blood pressure waveforms of the male volunteer in sets of 10 s, composed of 1000 estimations each, obtained in **c** the calibration session, **d** 2 h after calibration with removal and reapplication of the optoacoustic sensor, and **e** during the seated-core-hold maneuver. **f** A scatter plot comparing the results of the NOVA and optoacoustic measurements, where each point (*n* = 120) is an average over a 10-sec measurement. The light dashed lines represent the 95% agreement margins. **g** The distribution of the SBP and DBP values of each cardiac cycle recorded, showing large variation (male and female aggregated, *n* = 1460). **h** A Violin plot showing the error distributions (male and female aggregated, *n* = 120). **i** A Bland-Altman plot of the data set, *n* = 120.

The last set of in vivo measurements was performed on two volunteers (29-year-old male—slightly hypertensive, 42-year-old female—slightly hypotensive) to assess the ability of our optoacoustic sensor to estimate BP. All the measurements included a synchronized reading of the arterial diameter, obtained by the optoacoustic sensor affixed to the tissue via a Sondafix patch and ultrasound gel (Supplementary Fig. 43), alongside ground truth BP waveforms from a reference Finapres NOVA cuff-based system (Methods). The measurements were performed for two types of static positions: seated resting position and seated-core-hold position (Fig. 6a and Supplementary Note 14). Each measurement lasted 14 sec (trimmed to 10 sec—Methods) and in total 120 measurements were conducted over the course of three months. The diameter readings from the optoacoustic sensor were transformed into BP waveforms using Hayashi’s empirical model^32^, where the calibration values were determined once per subject, in a resting position, at the beginning of the study (Methods). After each measurement session, the optoacoustic sensor was removed and then later reapplied at approximately the same region in the subsequent session, without precise feedback on the position or orientation of the sensor. Thus, the optoacoustic measurements were performed with position and orientation uncertainty with respect to the calibration measurement, emulating how such a sensor would be positioned on the skin in real-life scenarios if integrated into a wearable device.

Figure 6b shows a comparison between the diastolic and systolic BP values of the optoacoustic and reference systems obtained over a span of 30 min that included the seated-core-hold position, where the calibration was performed several days earlier. As expected, an elevation in the BP was recorded for both systems during the seated-core-hold position. Figure 6c-e show representative BP waveforms of the two systems obtained at different sessions: the calibration session, a session performed two h after calibration, and a seated-core-hold session. The figures clearly show that the agreement between the two systems is maintained over the entire cardiac cycle and not only for the diastolic and systolic values. Figure 6f shows a scatterplot in which the reference diastolic and systolic BP values are compared to those measured by the optoacoustic sensor, revealing good agreement between the two. The total score of the system in terms of mean absolute difference (MAD) is 2.6 mmHg diastolic, and 4.7 mmHg systolic (Methods), comparable to values reported by leading wearable ultrasound BP patches in studies of similar participant numbers^7,20^, and within the range associated with high-grade device performance. Figure 6g shows a distribution of the diastolic and systolic BP values measured by the reference system, revealing a substantial variation that confirms that the sensor’s performance was evaluated far from the calibration points. Figure 6h shows the error distribution of the estimation of the systolic and diastolic BP values, while Fig. 6i is a Bland-Altman plot of the measurements. Additional results from the clinical study are summarized in Supplementary Fig. 45.

## Discussion

We have developed a paradigm for cuffless BP monitoring, based on a placement-insensitive optoacoustic sensor that directly measures the arterial diameter with high accuracy. Previous wearable devices for monitoring the arterial diameter have relied on piezoelectric ultrasound patches^20,21^, a technology that has shown high maturity and versatility in numerous applications^17,19^, in contrast to the limited performance and applicability of current optoacoustic patches^26,27^. Additionally, pulse-echo ultrasound has the inherent advantage of stronger acoustic signals in comparison to optoacoustics, which facilitates its transition to a wearable technology, where design constraints can lead to a reduction in signal magnitude.

Despite the greater maturity and conformability of current ultrasound technology for wearable applications, our work shows that the optoacoustic approach carries an inherent advantage for BP monitoring: the superior contrast it achieves between blood vessels and their surrounding tissue, offering a solution to the placement sensitivity of ultrasound patches. In pulse-echo ultrasound, the back-scatter signals from the blood vessels are comparable in magnitude to the surrounding tissue (Supplementary Figs. 46 and 47), requiring position and orientation-sensitive focusing to reject background signals. This challenge can be partially solved by using dense ultrasound arrays, in which at least one element is correctly positioned with respect to the artery, but this results in increased system complexity. The density of the array needs to be increased when targeting smaller arteries to maintain optimal overlap between the chosen element and both the posterior and anterior sides of the artery. In the case of the radial artery, the element pitch of leading patches appears insufficient for accurate estimation of the artery diameter (Supplementary Fig. 8). In optoacoustics, the signal from superficial, isolated blood vessels is often so strong that it surpasses all background signals, thereby obviating the need for exact detector alignment, and thus the need for arrays. To leverage the optoacoustic contrast advantage, omnidirectional detection is needed, which requires miniaturization levels that can be achieved in piezoelectric technology only at a severely reduced sensitivity^38^ that is inappropriate for optoacoustic applications.

The optoacoustic sensor developed in this work is enabled by the recently developed technology of SPADE^29,30,36^, which achieves both high sensitivity and omnidirectional detection, thus fulfilling the conditions for placement-insensitive sensing. In addition, the SPADE exhibits a high reception bandwidth of 125 MHz, which goes far beyond practical values required for this application. Specifically, the attenuation of the high acoustic frequencies (Supplementary Table 3) reduced the maximum bandwidth in our in vivo measurements to 28 MHz, corresponding to an acoustic wavelength of approximately 49 µm^31^. In comparison, the typical Transmission/Reception bandwidth and resolution of pulse-echo ultrasound based BP patches were 3 MHz and 231 µm, respectively^15,16,20,21^. Thus, although the full bandwidth of SPADE is not necessary to meet the practical requirements of this application, its ability to capture the entire usable acoustic bandwidth is advantageous, particularly because conventional pulse-echo approaches cannot access the higher-frequency components of the signal. An additional advantage to our approach is that the optoacoustic sensor measures the true lumen diameter, whereas the estimation via pulse-echo ultrasound also includes an unknown contribution from the artery wall thickness, which may change over time in hypertensive patients^39^. As shown in our phantom study, the high performance of the SPADE enabled diameter estimations with a typical uncertainty lower than 10 µm over a wide range of positions and orientations. In the clinical measurements, the effect of changing the position and orientation of the sensor was smaller than the beat-to-beat diameter variations and therefore could not be quantified. In contrast, when piezoelectric transducers were used, either in optoacoustic or pulse-echo mode, uncertainty values as high as 100 µm were achieved in the phantom measurement and even larger values were observed when the probe was translated or rotated above a pulsating artery.

To evaluate the performance of the optoacoustic sensor, it was clinically tested and compared with a cuffs-based BP monitor (brachial and finger). The testing was performed for over three months, where the positioning of the sensor was performed without alignment markers, requiring only the presence of a pulsatile M-mode signal, leading to an uncertainty in position and orientation that would be expected for a wearable device. The average MAD between the two devices, calculated over 120 measurements, was 3.6 mmHg, similar to previously reported values for pulse-echo ultrasound-based BP patches^7,20,21^. Possible contributing factors to the MAD include the approximate nature of the empirical model used to covert the diameters to BP values, inaccuracies in the reference devices^40,41^, and external force applied by the Sondafix patch. Full compliance with recent validation standards remains to be demonstrated in a larger clinical study.

The current study focused on BP monitoring in a resting position, following the AHA guidelines^42^. Nevertheless, our sensor is inherently motion-compatible: as shown in Fig. 4e, probe displacement does not impact the accuracy of the diameter estimation. Additionally, while variations in the coupling conditions may lead to fluctuations in the amplitude of the optoacoustic signals, they do not affect how the artery diameter is encoded in the delays of the signal. Finally, our robust wavelength stabilization scheme (Methods) for SPADE further supports reliable performance during motion.

Due to the preliminary nature of this proof-of-concept study (*n* = 2), statistical analysis of sex-based differences was not feasible. However, both a male and a female subject were purposefully included to establish initial sensor feasibility across different biological baselines. Future full-scale clinical validation (target *n* > 85) will be sufficiently powered to evaluate sex as a biological variable, as required by standards in this field.

Several improvements to the optoacoustic sensor could facilitate its application as a wearable device. On the hardware side, the table-top lasers used for the optoacoustic excitation and SPADE interrogation may be replaced with portable versions. For example, the bulky wavelength-tunable OPO source used in our work operated only at a single wavelength and delivered a modest pulse energy of 4.9 mJ to the skin, resulting in SNR above the required level, as tissue background noise exceeded SPADE’s intrinsic noise. Furthermore, as shown in Supplementary Fig. 42, the short pulse duration of the OPO source, 7 ns, was not essential in this work since it corresponds to an acoustic bandwidth of 63 MHz, which exceeds the bandwidth limitation imposed by acoustic attenuation. A longer pulse duration of 20 ns would lead to an intrinsic bandwidth of 21 MHz, a value that still exceeds the typical bandwidths expected for this sensing depth (Supplementary Fig. 41). Therefore, comparable performance may be achieved by compact laser diode arrays operating at the single wavelength, *e.g*., 808 nm^25,43^, or 908 nm^44^, which support configurable pulse energies and durations within the relevant ranges. Additionally, if two laser diodes with different wavelengths are used sequentially, the oxygen saturation inside the artery may be recovered, thus providing an additional relevant vital sign. Further miniaturization of the excitation source may also be achieved by vertical-cavity surface-emitting lasers (VCSELs), which may produce optoacoustic signals comparable to those of laser diodes^45^. The widely tunable external cavity laser (ECL) used in our study for the SPADE interrogation (Methods) may be replaced by a distributed feedback (DFB) laser diode that operates at a wavelength close to the optical resonator in SPADE. Moreover, following recent advances in silicon photonics, the entire interrogation system may be fabricated inside the SPADE chip, with the wavelength stabilization replaced with on-chip heaters^46–49^. Since only a single SPADE element is required, data acquisition can be performed using miniaturized, low-cost integrated circuits (e.g., ADC3563, TI, USA), simplifying the electronics compared to the multi-channel systems used in current ultrasound patches. Finally, improving the Q-factor of SPADE can lead to higher sensitivities, thus enabling the use of lower powers in the optoacoustic and interrogation lasers to further simplify the system. For example, while the Q-factor of the SPADE used in this work was 2 × 10^5^, values exceeding 10^6^ are commonly reported in Si_3_N_4_ platforms^50^.

We note that while the focus of our work was on demonstrating that BP monitoring can be performed with a single SPADE element, using a sparse array with a few elements^51,52^ may further enhance the performance. For instance, such an array could enhance the sensor’s robustness to positioning and enable assessment of arterial ellipticity under externally applied force. However, even a small number of sensing elements may increase overall system complexity in the case of parallel interrogation since it would require scaling up many of the system’s components. Serial interrogation can simplify the array configuration by using wavelength encoding, which will enable the fabrication of all the resonators on the same bus waveguide, thus sharing the same system components.

On the algorithmic side, it is important to note that the optoacoustic sensor provides access to the entire pulse pressure waveform rather than only discrete values corresponding to systolic and diastolic pressures. The full waveform carries rich information about cardiovascular dynamics that was not used in this work. Its morphology reflects arterial compliance, peripheral resistance, and wave-reflection phenomena—all of which can change with physiological variation, disease states, and therapeutic interventions^53,54^. Consequently, waveform-based estimation offers an opportunity for more robust operation than achieved by beat-to-beat extrema detection, since it leverages the continuous relation between arterial diameter and pressure across the cardiac cycle, facilitating the use of more complex models and deep-learning algorithms^55,56^. Furthermore, continuous access to the waveform opens avenues beyond pressure estimation. Features such as the slope of the systolic upstroke, the timing of the dicrotic notch, and subtle changes in pulse contour have been linked to left ventricular function, arterial stiffness, and autonomic regulation. Capturing these subtleties with high fidelity allows the same measurement to support a spectrum of applications, from early detection of vascular abnormalities to calibration-free BP estimation algorithms^57–59^. For example, in ref. ^57^ it was demonstrated that the calibration parameters in Hayashi’s empirical model, used in our work, may be estimated directly from features in the diameter waveform using multivariate analysis.

## Methods

### SPADE fabrication

SPADE is a technology for ultrasound detection developed in ref. ^29,30^, based on silicon or silicon nitride waveguides^36^. It possesses several features that are important for this work: high sensitivity, miniaturization, omnidirectional response, high signal fidelity, and large bandwidth. The main component in a SPADE system is the silicon photonics resonator coated with PDMS (Fig. 1g), in which acoustic pressure is converted to a modulation of the resonator wavelength. In this work, the resonator was based on a Bragg mirror, formed by side corrugation of a Si_3_N_4_ waveguide, and a localized defect region with a Gaussian profile (Supplementary Fig. 26a) that generated the resonance with a Q-factor of 2 × 10^5^. While higher Q-factors have been reported for Si_3_N_4_ micro-rings^50^, this often comes at the cost of large ring diameters—hundreds of microns, which leads to focused detection^60^. In contrast, the light localization achieved in our Bragg-cavity design enables point-like, omnidirectional detection (Supplementary Table 4), which is essential for robust detection of arterial signals regardless of probe positioning. The Si_3_N_4_ waveguide core had a width of 1 µm, height of 350 nm, and a side-corrugation period of 490 nm. The waveguide was terminated on both ends with apodized grating couplers to enable robust fiber coupling to the chip. The considerations in choosing the fiber-coupling approach are presented in Supplementary Note 15. The fabrication was performed using the multi-project wafer (MPW) service of Ligentec (Switzerland).

After the MPW fabrication, several post-processing procedures were performed to enable our application. First, the top silicon oxide (TOX) cladding was removed using wet etching (BOA 1:7) until the waveguide was partially exposed, etching only 20 nm of the embedded oxide layer. To avoid over-etching, the process was monitored via reflectometry (ATF 2100, NanoSpec, Japan) and stylus profilometry (Bruker, USA). In the next step, the chip was diced (DAD3350, Disco, Japan) to a width of 1.5 mm (Supplementary Fig. 26a) and cleaned of organic residues using Piranha solution. The chip was then dried at 300 °C for 10 min and placed in an O_2_ plasma asher (HF8, Axic, USA) for 5 min to enhance the adhesion to the over-cladding, which comes next.

The resonator region was coated with PDMS (SYLGARD 184, Dow, USA) using spin coating (3 min at 10 K RPM resulting in a thickness of 3 µm). This common type of PDMS was selected over several alternatives (SYLGARD 527, NuSil LS-6943) because it better protects the SPADE chip from potential damage when it directly contacts the skin. Its adhesion to the chip was sufficiently strong to prevent tear offs while handling and enabled the use of ultrasound gel without performance degradation for over 2 months. To ensure robust and optically stable coupling, a Kapton tape mask was used to prevent PDMS from coating the grating couplers. After the PDMS coating, the chip was left overnight to degas in cryogenic vacuum ( < 5 µTorr) and was finally baked in an oven to cure the PDMS coating (30 min at 80 °C). The chip was then glued (NOA61, Norland, USA) to a 0.5 mm thick glass slide (25 × 25 mm, Yixing Purshee Optical Elements, China). The glue is then cured with UV light for 10 min (CS20K2, Thorlabs, USA).

### SPADE assembly

Using a fiber polisher (TRIG, KRELLTECH, USA), two polarization-maintaining fibers (AFW technologies, Australia) were polished to a 48° angle ($\alpha=+ 3^\circ$ in Supplementary Fig. 27d). The facets of the polished fibers were first coated with gold (200 nm using PVD, Temescal, USA) and then bonded to the grating coupler with Norland NOA61, which completely covered their coated tips. This procedure ensured that the gold coating is buried inside a protective layer, preventing it from being rubbed off. The Norland NOA61 adhesive was selected for its strong adhesion to silica, minimal optical absorption and excellent clarity in the C-L band, low viscosity, near-zero shrinkage, and non-toxicity.

The fiber-to-chip coupling system (Supplementary Fig. 36) involved 6-axis micrometric manual stages, as the 6 dB insertion loss per-coupler required a misalignment smaller than 1 µm in planar positioning, and smaller than 0.5° in the pitch, yaw and roll angles. Also included in the procedure is a microscope objective lens (×10, Mitutoyo, Japan) connected to a camera (15103, Allied Vision, Germany), broadband illumination (Fiber-Lite MI-LED A7, Dolan-Jenner, USA), and a tunable CW laser with a power meter (81606A, Keysight, USA) used to assess the transmission spectrum. In the first step of the alignment, a multimode (MM) fiber, connected to the power meter, was passively aligned above the output grating coupler using feedback from the camera and a PM fiber, connected to the CW laser, was actively aligned to the input grating coupler using feedback from the power meter and bonded to the chip (Supplementary Fig. 27). We note that the MM fiber required only passive alignment due to its large core and high numerical aperture, leading to much looser tolerances in comparison to the PM fibers. In the second step, the MM fiber was replaced by the second PM fiber, which was actively aligned and bonded to the output coupler.

After the initial bonding, the fibers were further protected with a polymer coating along their length to protect them from contact with the skin. Rigid parts, from the end of the chip to the end of the glass slide, were encapsulated with the UV cured OP-67-LS (Dymax, USA), chosen for its strong adhesion to silica, fast curing, low shrinkage, and extreme hardness. Flexible parts were recoated with the Desolite 950-200 (Covestro AG, Germany), used to prevent them from breaking where the original jacket was removed. Supplementary Figs. 27 and 28 illustrate the encapsulation process. The minimal distance between the couplers for which this procedure was found possible is 0.5 mm.

The glass slide with the SPADE was then attached to the illumination bundle with Kapton tape. Between usages the tape was removed, and the slide was rinsed with both deionized water to remove ultrasound gel residue, and ethanol for disinfection. The two PM fibers were fixed to the illumination fiber bundle with scotch cable ties, keeping them organized while preventing tensile stress.

### SPADE readout

The theoretical optical transmission spectrum of such a resonator and the interrogation system used with that device are shown in Supplementary Fig. 29, where a CW interrogation laser is actively tuned to the maximal transmission wavelength. In this state, light is localized at the center of the resonator, and acoustic sensing is localized with it. Pressure changes in the vicinity of the resonator change its inherent resonance wavelength and consequently change the phase through it. The sensor’s responsivity is the conversion efficiency from pressure to phase modulation, and it is proportional to the steepness of the phase slope (Supplementary Fig. 29a), which, in turn, is proportional to the resonator’s Q factor.

A Mach Zehnder Interferometer (MZI) with a differential photo detector is used to detect the US induced phase changes through the sensor, using the scheme in ref. ^29^. The MZI is balanced, *i.e*., the phase delay in both its arms is approximately equal, thus leading to substantial cancellation of the CW laser’s phase noise^29^. A fiber stretcher (Paulsson, USA) is driven by a feedback circuit with a frequency response of 0−10 kHz to lock the MZI to a quadrature^61^, in which the power delivered to the two photodetectors is equal, leading to a zero differential signal in the absence of external perturbations. Since the ultrasound signals contain mostly frequencies far greater than the bandwidth of the feedback circuit, the perturbations they induce in the resonance wavelength are translated into phase modulation of the CW laser at the resonator output, pushing the MZI slightly out of quadrature and creating a differential voltage signal proportional to the magnitude of the ultrasound signal.

### Laser wavelength stabilization to enable wearability

Because of the high-Q factor of the resonator and the high photo-elastic and thermo-optic coefficients of PDMS^62,63^, even minute pressure or temperature variations in the optoacoustic sensor may shift the resonator’s resonance wavelength so that it no longer matches the laser wavelength, leading to loss of signal. To enable continuous operation of the sensor as a wearable device, a feedback system was developed to lock the laser to the resonator to overcome the external disturbances: pressure variations while the sensor was dragged on the skin until a beating signal was found; temperature variations during the application of the ultrasound gel to the optoacoustic sensor and first contact with the skin; and temperature fluctuations due to the partial absorption of the pulsed optoacoustic illumination by elements in the optoacoustic sensor.

The large wavelength drifts (up to 1 nm s^−1^) due to temperature changes and the rapid fluctuation due to applied pressure required a two-tier stabilization procedure described in Supplementary Fig. 30. The interrogation laser (a diffraction-grating based ECL, TSL-550, Santec, Japan) included two wavelength tuning mechanisms: coarse digital tuning with a discrete resolution of 0.1 pm and span of 130 nm, in addition to fine analog continuous tuning with a span of 120 pm and maximal slew rate of 30 nm s^−1^. The first tier of stabilization was based on the dither locking (Supplementary Fig. 30), using the laser’s continuous analog tuning in tandem with a FPGA-based system (Moku:Go, Liquide instruments, Australia) to overcome rapid fluctuations in the resonance wavelength. The second tier of stabilization used an Arduino controller and the laser’s digital tuning to follow slow drifts in the resonance wavelength to ensure that the feedback signal of the first tier does not exceed the laser’s fine-tuning range. The process was monitored with the 2204 A (PicoScope, UK) oscilloscope.

### Optoacoustic illumination

The excitation laser source is an OPO pulse laser (SplitLight EVO I, InnoLas, Germany) with pulse width of 7 ns, repetition rate of 100 Hz, maximum pulse power of 30 mJ, and wavelength tuning capabilities from 680 to 980 nm. In all the measurements, the laser operated at a wavelength of 800 nm. The laser is connected to a custom-designed fiber bundle (Armadillo SIA, USA) beginning with a round input ferule (active area 5.5 mm in diameter) that splits into two rectangular outputs with dimensions of 1.5 × 8 mm each. The laser pulse energy was reduced to 10 mJ, and its free-space beam was coupled to the fiber bundle with 60% end to end efficiency. A custom-made mirror at the two outputs reflected the light vertically into the skin and expanded the width of the beam from 1.5 to 6 mm on skin’s surface (Supplementary Fig. 26). This split illumination profile was selected to allow for dark-field conditions^64^, directing more light around the vessel, thus—boosting the amplitude of the posterior boundary (Supplementary Fig. 23b). The mirror was produced from aluminum using CNC, polished and gold coated (PVD, Temescal, USA) to achieve high reflectivity in NIR^65^. Because not all the light from the bundle reached the mirror and the imperfect polishing of the mirror, the total pulse energy that reached the tissue was measured (PM100USB and S425C, Thorlabs, USA) to be 4.9 mJ, fulfilling the ANSI Z136.1 safety requirements (Supplementary Note 9). The thickness of the illumination probe was 3 mm, leading to a total thickness of 3.5 mm for the optoacoustic sensor when accounting for the 0.5 mm thickness of the glass slide on which the SPADE was bonded.

### Finapres NOVA as the reference BP monitor

Following the lines or ref. ^7^, we have employed the Finapres NOVA (Finapres Medical Systems, the Netherlands) system. Finapres NOVA is an FDA approved cuffed BP estimation system that is applied to the finger and produces the full BP waveform in real time. Photographs of the system are shown in Supplementary Fig. 31. Although the system can correct BP variations due to changes in the height of the finger by using a height compensation unit (HCU) and a physiological model, all the measurements were performed while keeping the hand at the heart’s height. The system’s “Physiocal” function was used throughout, as it improves accuracy by periodically deflating the finger cuff to reestablish a baseline. The frequency of these events depends on the patient’s state and generally reduces as the measurement progresses. These events induced breaks in BP waveforms (1-2 cardiac cycles long), which were automatically interpolated from adjacent cycles.

An automatic calibration procedure with a brachial cuff (“BraCal”) was performed at the beginning of each session, where sessions lasted no more than 30 min. The brachial cuff was deployed on the left arm (same arm onto which the finger cuff and our sensor are deployed), as calibration with the contralateral arm is less accurate. When the brachial cuff was inflating, radial readings were paused. While not required, using BraCal is a condition for achieving an accuracy of 3 mmHg for the NOVA system, as disclosed in the system’s ISO 81060-2 certification. BraCal allows for the accurate generation of the full brachial BP waveform from the finger waveforms (“reBAP” algorithm), thus making the readings comparable to conventional brachial cuff systems. This automatic conversion is performed in real-time using generalized waveform filtering and level correction^66,67^. The brachial waveforms lag 4 seconds behind the finger waveform, requiring 14 sec readings to produce trimmed 10 sec blocks. The accuracy of the conversion, as described in published works^68,69^, is 3 ± 8 mmHg (SBP) and 4 ± 6 mmHg (DBP). Additionally, our own accuracy evaluation is given in Supplementary Fig. 32.

The NOVA system in our possession was calibrated by the manufacturer before the experiments, and all peripheral accessories were tested and certified (finger cuffs were checked for leaks, HCU and I/O interface were calibrated). Despite known physiological similarities between the finger and radial BP waveform^70^, it is still desired that the reference BP monitor produces the radial BP waveform, which is the one measured by our optoacoustic sensor. Accordingly, the finger and brachial BP waveforms produced by the NOVA system were used to computationally produce the radial waveform via MAP-anchored interpolation^71–74^. A typical result of this conversion is shown in Supplementary Fig. 33.

### Shielding Finapres PPG from optoacoustic pulses

The Finapres NOVA system employes a NIR PPG sensor at the finger cuff as part of its volume-clamp method^75^. Our optoacoustic excitation, which also operated in the NIR was found to interfere drastically with the operation of that sensor due to its high intensity the similar wavelength. To resolve this issue, we used a finger cot (Supplementary Fig. 34), custom made from laser absorbing fabric (BK5, Thorlabs, USA), to shield the finger cuff from the disruptive excitation light. The clear tubing connecting the cuff with the local pump module had to be protected with dark sleeves, as they acted as optical fibers, directing stray light into the sleeve. The cot was very loosely fitted to prevent any alteration to blood flow or cuff inflation.

### Signal acquisition

The data acquisition for the BP waveforms was performed using the 5444D (PicoScope, UK) oscilloscope at a rate of 250 MS s^−1^ with a depth of 12 bits. After every trigger event (100 Hz), 7 µs of acoustic signal was captured, corresponding to a depth of 1 cm in tissue. Three signals were captured: the pressure signal from the SPADE readout system (Supplementary Fig. 29), and the finger and brachial reference BP waveforms from the NOVA system. Supplementary Figs. 35 and 36 present, respectively, the system schematics and a photograph of the complete setup. MATLAB R2024b was used for orchestration.

In addition to the acquisition of the BP waveforms, an auxiliary sampling device (UCA222, Behringer, Germany) was used to capture a 2-lead ECG signal. The ECG acquisition was synchronized with the BP monitors by using the same trigger for both sampling devices (Supplementary Fig. 37). The synchronized measurement of the radial waveform by the optoacoustic sensor and the ECG signal enables the system to capture the pulse arrival time and pulse wave velocity (Supplementary Fig. 38), parameters associated with BP values, which are used in some physiological models (Supplementary Note 3).

### Signal processing

The system acquires 100 BP readings per second but does not output them in real time. Instead, data is processed in 10-sec blocks and handled in parallel with the acquisition to maintain continuous sampling (with ≤0.2 sec gaps between blocks; Supplementary Fig. 39a). After acquisition, the processing routine is triggered automatically and performed in MATLAB 2024b. The raw signals (Supplementary Fig. 39c) contain a background signal, formed of acoustic interferences from sensor and tissue optoacoustic signals, and noise that is shot-noise-dominated. The background signals are approximately twice the intensity of the shot noise. Data can be visualized either as 1D waveforms or 2D images (Supplementary Fig. 39b). Processing begins with twofold interpolation of the sampled signals, followed by a 600 kHz high-pass filter to remove non-ultrasound components. The non-pulsatile baseline is subtracted using the median profile of the measurement. Noise is suppressed using Savitzky-Golay filtering^76^ in both dimensions and BM3D^77^ denoising in 2D. Finally, unsharp masking along the time dimension enhances the pulsatile signal (Supplementary Fig. 39d).

To isolate the arterial optoacoustic signal, the conditioned signal is multiplied by a window whose temporal support is automatically determined via a morphology-aware, multi-scale approach. This procedure effectively truncates preceding stray signals and trailing acoustic reflections. The temporal boundaries of the anterior and posterior signal edges were localized to within 50 ns, after which the exact positions of the inflection and minimum points were extracted automatically, as described in the following method. Off-line processing allowed us to verify, and in 5% of cases manually correct, the automatic boundary localization; manual correction was straightforward, as the true boundary is unambiguous upon visual inspection. Localization failures were attributable to residual background and acoustic reflection signals and occurred with approximately equal prevalence at the anterior and posterior boundaries. No trigger events were rejected, and all were correctly localized prior to the final inflection/minimum extraction stage, where no intervention was performed. This deterministic algorithm is a natural candidate for replacement by a learning-based approach—one that, given the visual unambiguity of the boundary, would readily yield a near-perfect, fully automated detection when trained on data from a future large-scale study.

### Diameter estimation

For an ideal optoacoustic signal from a cylindrical source, the diameter may be estimated by locating the two discontinuities in the signal. In practice, the measured signal undergoes a low-pass operation due to several reasons: finite duration of the optoacoustic pulses, limited bandwidth of the acoustic detector, and acoustic attenuation of high frequencies in the tissue. This low-pass operation affects the two discontinuities in different ways (Supplementary Fig. 40). In the early discontinuity, the signal jumps from zero to a maximum value, and if the low-pass kernel is symmetric, its position can be determined by finding the inflection point in the low-passed signal. The simulations show that for an effective −6 dB bandwidth of 6 MHz or higher, the inflection point coincides with the theoretical discontinuity. In the late discontinuity, the analytical solution diverges to minus infinity, and the location of the discontinuity is identified by finding the minimum in the low-passed signal, which also remains invariant for an effective −6 dB bandwidth of 6 MHz or higher. As shown in Supplementary Fig. 41, despite the effect of acoustic attenuation at high frequencies, the measurement bandwidth remains above 6 MHz for measurements offset of 10 mm, justifying our choice of estimation method.

In Supplementary Fig. 40, we evaluated the stability of the discontinuity points. The high-bandwidth simulated acoustic signal was filtered using Gaussian kernels corresponding to various bandwidths. The relationships between the Gaussian kernel standard deviation (STD), its rise time, the kernel’s −6 dB bandwidth, and the rise time of the corresponding step signal (error function) are detailed in Supplementary Table 5 and Supplementary Fig. 42b.

### Blood pressure estimation from diameter readings

Blood pressure estimation from the arterial diameter, measured by the optoacoustic sensor, is performed using Hayashi’s empirical model for transmural pressure

1 $$p\left(t\right)={p}_{{\rm{dia}}}\cdot \exp \left[\beta \left(\frac{{D}^{2}\left(t\right)}{{D}_{{\rm{dia}}}^{2}}-1\right)\right]$$

where $D\left(t\right)$ and $p\left(t\right)$ are diameter and pressure waveforms, respectively, and ${p}_{{\rm{dia}}},\beta,{D}_{{\rm{dia}}}$ are the calibration parameters, which are calculated from a 10-sec synchronized measurement of the optoacoustic sensor and reference Finapres NOVA system. Specifically, the average values of the diastolic diameter (${D}_{{\rm{dia}}}$) and pressure $({p}_{\rm{dia}})$ over the 10-sec measurement are used to calculate β using the following formula:

2 $$\beta=\frac{\mathrm{ln}({p}_{{\rm{sys}}}/{p}_{{\rm{dia}}})}{{\left({D}_{{\rm{sys}}}/{D}_{{\rm{dia}}}\right)}^{2}\,-1}$$

The calibration was performed once, and the same calibration parameters were used to convert the diameter waveform from the optoacoustic sensor to a BP waveform.

### Clinical testing

The clinical tests were conducted with adherence to the approval provided the intuitional review board at the Technion—Israel institute of technology (FWA-00013345). Informed consent was obtained from all human research participants. The test room was regulated to 24 °C and was quiet and free of distractions. Information about the test was clearly displayed. In the test protocol, the subject first puts on the measurement gear: ECG contacts, brachial BP cuff, NOVA finger cuff and finger cot. Cuff sizes (brachial and finger) are chosen to match from three optional sizes. The measurement session is preceded by a 5-min relaxation period in which the volunteer remains in a seated position with the laser safety goggles on. The measurement begins with a calibration of the NOVA system with the brachial cuff, which is followed by the application optoacoustic sensor, and by successive 14-sec readings in which the NOVA pressure waveforms and the optoacoustic sensor data are collected. Durning the data acquisition, the subject remains motionless while assuming the seating position or the seated-core-hold position.

### Blood pressure performance evaluation

The performance of the optoacoustic sensor as a BP monitor was analyzed in accordance with the IEEE 1708a standard^78,79^. Statistical performance evaluation was performed by finding the systolic and diastolic pressures for each cardiac cycle and then averaging 10-seconds worth of consecutive cycles to measure the systolic and diastolic BP pairs from the optoacoustic system $({{SBP}}_{{\rm{OA}}},{{DBP}}_{{\rm{OA}}})$ and from the NOVA reference system $({{SBP}}_{{\rm{REF}}},{{DBP}}_{{\rm{REF}}})$. For a set of N 10-second readings, the following mean absolute difference (MAD) metrics were calculated:

3 $${MA}{D}_{{\rm{SBP}}}=\frac{{\sum }_{n=1}^{N}\left|{{SBP}}_{{\rm{REF}}}^{n}-{{SBP}}_{{\rm{OA}}}^{n}\right|}{N},$$

4 $${MA}{D}_{{\rm{DBP}}}=\frac{{\sum }_{n=1}^{N}\left|{{DBP}}_{{\rm{REF}}}^{n}-{{DBP}}_{{\rm{OA}}}^{n}\right|}{N},$$

### Reporting summary

Further information on research design is available in the Nature Portfolio Reporting Summary linked to this article.

## Supplementary information

Supplementary Information

Reporting Summary

Transparent Peer Review file

## Source data

Source Data

## Data Availability

The main data supporting the findings of this study are available within the article and its Supplementary Information. Additional data are available at https://doi.org/10.5281/zenodo.20841180^80^. Source data are provided with this paper.
