# Supplementary material for: Silicon-photonics optoacoustic sensor for fully wearable blood pressure monitoring
Source: Nat Commun. 2026 Sep 17;17:10931. doi: 10.1038/s41467-026-77580-3 (PMC13680363; doi:10.1038/s41467-026-77580-3)
Supplement: Supplementary file 1 [file 41467_2026_77580_MOESM1_ESM.pdf]

Silicon-photonics optoacoustic sensor for fully wearable blood pressure monitoring

Supplementary Information

Gil Gelbert[1], Ron Moisseev[1], Yoav Hazan[1], Tamar Harary[1], Daniel H. Lange[1a], and Amir Rosenthal[1a*]

[1] Andrew and Erna Viterbi Faculty of Electrical & Computer Engineering, Technion - Israel Institute of Technology, Haifa 32000, Israel.

[1a] These authors jointly supervised this work.

* Author to whom correspondence should be addressed: amir.r@technion.ac.il.

Contents

Supplementary Notes 4
- Supplementary Note 1 – Blood pressure fundamentals 4
- Supplementary Note 2 – Cuffed blood pressure estimation 5
- Supplementary Note 3 – Cuffless blood pressure estimation 6
  - Estimation standards 6
  - Estimation systems 7
  - Estimation models 8
- Supplementary Note 4 – Limitations of PPG based systems 10
- Supplementary Note 5 – Pulse-echo ultrasound diameter estimation 11
  - Tx/Rx both using individual elements 11
  - Planewave Tx / individual element Rx 12
  - Tx/Rx both using planewaves 12
- Supplementary Note 6 – Optoacoustic patches 13
- Supplementary Note 7 – The optoacoustic signal of a uniform cylinder 14
- Supplementary Note 8 – Optoacoustic simulations 15
- Supplementary Note 9 – Safety 16
- Supplementary Note 10 – SPADE characterization (NEP, BW, PSF) 17
- Supplementary Note 11 – Phantom experiments with SPADE 18
- Supplementary Note 12 – Error propagation 19
- Supplementary Note 13 – Piezoelectric optoacoustic imager 20
- Supplementary Note 14 – Inducing BP changes 21
- Supplementary Note 15 – Grating couplers 22

Supplementary Tables 23
- Supplementary Table 1 – Comparing cuffless blood pressure estimation standards with this work 23
- Supplementary Table 2 – Comparing wearable optoacoustic platforms with this work 24

Supplementary Table 3 – Acoustic properties of simulation and phantom materials 25
Supplementary Table 4 – Comparing a piezoelectric sensor to SPADE 26
Supplementary Table 5 – Gaussian kernel relations 27
Supplementary Figures 28
Supplementary Figure 1 – Properties of an artery 28
Supplementary Figure 2 – Sphygmomanometery principle of operation 29
Supplementary Figure 3 – PPG and PWV.. 30
Supplementary Figure 4 – PPG`s susceptibility to inert changes 31
Supplementary Figure 5 – PAT from PPG 32
Supplementary Figure 6 – The Telemed system 33
Supplementary Figure 7 – Ultrasound imaging of the wrist vessels... 34
Supplementary Figure 8 – Pulse-echo diameter estimation with Tx/Rx using individual elements..... 35
Supplementary Figure 9 – Time of flight principle in optoacoustics 36
Supplementary Figure 10 – The optoacoustic signal of a cylindrical source..................................... 37
Supplementary Figure 11 – Diffuse-optics Monte-Carlo simulation 38
Supplementary Figure 12 – Fluence distribution 39
Supplementary Figure 13 – NEP, BW, and PSF evaluation systems 40
Supplementary Figure 14 – Phantom experiment with SPADE.. 41
Supplementary Figure 15 – Phantoms imaging and 1D signals from SPADE and from piezoelectric sensors.. 42
Supplementary Figure 16 – Pitch angles of the radial artery 43
Supplementary Figure 17 – Phantoms diameter estimation with SPADE... 44
Supplementary Figure 18 – Blood pressure model error propagation... 45
Supplementary Figure 19 – The piezoelectric optoacoustic imager (PE-OA) 46
Supplementary Figure 20 – Piezoelectric optoacoustic *in vivo* imaging.. 47
Supplementary Figure 21 - Piezoelectric optoacoustics yaw sensitivity... 48
Supplementary Figure 22 – Phantoms diameter estimation with piezoelectric optoacoustics 49
Supplementary Figure 23 – |Max/Min| ratio of the optoacoustic signal 50
Supplementary Figure 24 – *In vivo* diameter estimation at different offsets using a single element of a piezoelectric optoacoustic probe with ideal orientation 51
Supplementary Figure 25 – *In vivo* diameter estimation with a piezoelectric transducer in optoacoustic and pulse-echo modes under non-zero offset and yaw angles.................................. 52
Supplementary Figure 26 – Photographs of SPADE and the fiber bundle... 53
Supplementary Figure 27 – Sensor coupling 54
Supplementary Figure 28 – Photographs of the optoacoustic sensor 55
Supplementary Figure 29 – The SPADE resonator and readout system 56

Supplementary Figure 30 – Laser stabilization scheme 57
Supplementary Figure 31 – The Finapres NOVA system 58
Supplementary Figure 32 – Validation of NOVA brachial BP accuracy 59
Supplementary Figure 33 – Brachial, radial, and finger BP waveforms 60
Supplementary Figure 34 – Finger blackout cot 61
Supplementary Figure 35 – System layout schematic 62
Supplementary Figure 36 – Photographs of the readout system 63
Supplementary Figure 37 – Data acquisition and synchronization 64
Supplementary Figure 38 – Recording with ECG and extracted PAT 65
Supplementary Figure 39 – Optoacoustic signal acquisition and processing 66
Supplementary Figure 40 – Bandwidth-limited diameter estimation 67
Supplementary Figure 41 – Bandwidth changes with offset 68
Supplementary Figure 42 – Bandwidth and resolution of sensors 69
Supplementary Figure 43 – Photographs of the optoacoustic sensor as a wearable device 70
Supplementary Figure 44 – Inducing blood pressure changes 71
Supplementary Figure 45 – Additional BP estimation plots 72
Supplementary Figure 46 – Pulse-echo diameter estimation with planewave Tx / individual element Rx 73
Supplementary Figure 47 – Pulse-echo diameter estimation with Tx/Rx using planewaves 74
Supplementary References 75

Supplementary Notes

Supplementary Note 1 – Blood pressure fundamentals

Blood circulation follows a two-phase cardiac cycle: *diastole* (ventricular filling) and *systole* (ventricular ejection), together constituting a single heartbeat. Blood is pumped from the heart into the aorta, which branches extensively into the arterial tree, terminating at the arterioles where the microcirculation connects to the venous system. The venous system then returns deoxygenated blood to the heart. The vascular network can be modelled as a system of compliant tubes of varying diameter, elasticity, and branching geometry (Supplementary Fig. 1), whose properties depend on anatomical location, age, disease state, and vascular tone. Within this network, blood, treated as an incompressible viscous fluid, flows with characteristic parameters including velocity, pressure, and shear stress, with regimes ranging from laminar to turbulent flow. These hemodynamic principles are extensively reviewed in Ref.[1].

Blood pressure (BP) can be measured at multiple sites along the vascular tree, but its interpretation depends critically on measurement conditions. In clinical practice, "blood pressure" conventionally refers to arterial pressure measured at the brachial artery with the arm supported at heart level under standardized conditions[2]. These conditions, covering patient position, environment, and measurement method, are codified in clinical guidelines to minimize transient effects and ensure comparability with reference values. Deviations from these conditions reduce clinical utility and may preclude actionable interpretation.

Examples of non-standard measurements include:

1. BP during physical activity, which is relevant mainly in exercise testing[3,4] but of limited general clinical value.
2. BP at non-brachial sites, *e.g.,* aortic pressure[5], which is used in specialized settings but differs from brachial BP.
3. BP in non-standard positions[6], *e.g.,* supine measurements require correction for hydrostatic bias.

Commercial BP devices and regulatory standards are aligned with this standardized context. Regarding motion particularly, some automated devices, such as those by OMRON (Japan), actively withhold readings if motion or speech are detected to preserve measurement integrity. Motion also makes manual sphygmomanometry difficult, unreliable, and it extends beyond its clinical guidelines. Finally, arterial-line BP monitoring is incompatible with subject motion. A healthy volunteer fitted with an arterial line for device testing would be required to remain still, and such an arrangement is itself strongly discouraged by the standards and misaligned with good clinical-study practice. On the other hand, a patient sick enough to warrant an arterial line is too ill to move.

Supplementary Note 2 – Cuffed blood pressure estimation

Blood pressure (BP) measurement is performed using a limited set of methods recognized as accurate and clinically valid, which can be divided into invasive and non-invasive approaches. Invasive methods involve direct arterial access via catheterization (arterial line, A-line), where a fluid-filled catheter-tubing system transmits the arterial pressure to an external pressure sensor positioned at heart level. This approach provides continuous, high-fidelity BP waveforms and is widely used in cardiology across patient conditions, but its invasiveness restricts its application to specific clinical scenarios. Alternative minimally-invasive approaches include extravascular ultrasound combined with microbubble contrast injection[7–9].

Non-invasive measurement relies on sphygmomanometry. A cuff applied to an extremity, typically the upper arm, occludes arterial flow, and the detection of arterial blood-flow sounds allows determination of systolic and diastolic pressures (Supplementary Fig. 2). Traditional manual sphygmomanometers used a hand-inflated cuff, a stethoscope, and a pressure gauge (sometimes mercury-based), whereas modern automatic devices employ computer-controlled inflation with electronic detection of Korotkoff sounds. Automatic sphygmomanometers achieve accuracies exceeding natural BP variability and provide discrete readings, typically once per minute, without requiring clinical supervision. Manual systems remain relevant in standards such as IEEE 1708 for the evaluation of cuffless BP devices[10,11].

Supplementary Note 3 – Cuffless blood pressure estimation

Cuffed BP devices provide accurate measurements for most clinical applications but have several limitations. They are uncomfortable, particularly for individuals with elevated BP, and prolonged use, such as in 24-hour ambulatory monitoring, can disrupt sleep. Measurements are intermittent, and the devices do not provide the full BP waveform, which could offer additional insights into cardiovascular health.

From a design perspective, the ideal cuffless BP system would be non-invasive, comfortable, and safe. It would measure the conventionally defined clinically meaningful BP with validated accuracy, be suitable for use by the general population and be operable without professional assistance. It would potentially provide beat-by-beat readings or even the full BP waveforms, alongside additional hemodynamic parameters such as stroke volume and oxygen saturation. Achieving these criteria has remained an unsolved challenge for over 40 years.

Successful cuffless BP monitoring requires three fundamental components: a universally accepted estimation model, standardized evaluation criteria, and a measurement system compatible with the model. To date, none of these components have been fully realized, and no system has met all fundamental requirements. Early efforts relied on rudimentary models derived from physics and limited measurements, with progressive improvements in sensor technology enabling more sophisticated models. Advances in computing and data storage facilitated iterative refinement of both models and systems, allowing for increasingly complex and validated approaches.

Many studies over the years have explored these approaches, reporting varying levels of validation for cuffless BP estimation. However, the absence of widely accepted evaluation standards limited the comparability and clinical relevance of these results. The introduction of the first standards[12] established evaluation criteria, providing a framework to guide development and assessment. Successive updates and amendments to these standards have continued to raise performance expectations, revealing limitations of earlier designs. Currently, no commercially available system fully complies with the most recent standards. Some devices were certified under earlier standards, while many consumer products, such as smartwatches, do not claim clinical validity in their BP measurements; nevertheless, they retain significant market share. The medical community does not consider readings from these devices reliable for clinical decision-making.

We review these three cornerstones - estimation models, evaluation standards, and measurement systems - while positioning our main contribution within the context of measurement systems.

Estimation standards

We focus on the most recent standards for cuffless blood pressure devices (The ISO 81060-2 by itself is not for cuffless devices), which serve as our reference: The 2019 version of the IEEE 1708 standard for wearable, cuffless blood pressure measuring devices[10,11], and ISO 81060-3[13]. A concise review of the primary components of these standards and our position with respect to each is provided in Supplementary Table 1. While we do not claim full compliance, our work prioritizes directions aligned with these standards, in contrast to some studies that performed extensive measurements not evaluated under the current criteria[12].

Estimation systems

Noninvasive estimation systems typically measure a combination of bodily properties with varying degrees of accuracy and fidelity. These properties serve as the building blocks for estimation models. Some are straightforward to obtain, others are readily accessible through existing technologies, while some remain challenging to measure.

Straightforward

1. Elevation of measurement site with relation to the heart.
2. Patient information - sex, age, BMI, ethnicity etc.
3. Body temperature (The blood`s speed of sound depends on its temperature[14])

Readily accessible

4. PAT - pulse arrival time. The time that passes from the electrical activity that induced systole at the heart until the pressure wave reaches a selected point at some extremity. Sometimes referred to as PTT (pulse transient time).
5. PWV - pulse wave velocity. The speed at which the pressure waves travel through the arteries over a given path.
6. ECG - electrocardiogram signal. The recording of the electrical activity of the heart.
7. PPG waveform features - morphological indices such as amplitude, rise time, dicrotic notch, reflection index, and augmentation index.
8. F_{EXT} - contact force at the measurement site.
9. F_{INT} - force exerted outwards through the skin by an artery.

Challenging

10. D - arterial diameter as a function of time in a selected measurement site. Often, only a corelative quantity is measured, for a lack of an appropriate modality.

It is important to note that the most challenging property to measure, the arterial diameter, is also the most critical for an accurate estimation model. In contrast, the easier properties are inexpensive and widely accessible. Force (internal or external) can be measured with resistive, capacitive, piezoelectric, optical and MEMS based sensors. ECG signals can be measured with conventional systems, and PAT/PTT/PWV are primarily measured via PPG sensors. Diameter estimation, however, requires modalities such as photoacoustics or pulse-echo ultrasound. The difficulty of measuring arterial diameter is reflected in the prevalence of studies that either omit it in favor of PWV, or measure correlated proxies such as PPG or bioimpedance[15,16]. While some of these systems employ machine learning and report reasonable accuracy, none fully adhere to relevant standards, limiting objective evaluation. It is generally advantageous to incorporate multiple properties, motivating systems that combine auxiliary modalities with a primary measurement. Consistently, we collect synchronized ECG signals to calculate PAT. Although our current estimation model does not use these signals; they are recorded for potential future integration (Supplementary Figs. 37 and 38).

Estimation models

Due to the physiological complexity of arterial pulsation, numerous models exist for evaluating blood pressure from measurable parameters. We focus on several widely used approaches, while noting that many other variations are also available[17,18]. Recently, several of the following models were combined and served as leaves in a decision tree[15], improving accuracy despite relying on simple modalities, a trend which might grow in popularity in light of recent advancements in ML[19].

Hayashi empirical exponential pressure-diameter relation

This phenomenological equation[20–22] assigns an artery a stiffness index β and corelates it with its transmural pressure and diameter.

$$\beta = \frac{\ln(p_{\mathrm{sys}}/p_{\mathrm{dia}})}{\left(D_{\mathrm{sys}}/D_{\mathrm{dia}}\right)^2 - 1} \rightarrow p(t) = p_{\mathrm{dia}} \cdot \exp\left[\beta\left(\frac{D^2(t)}{D_{\mathrm{dia}}^2} - 1\right)\right] \quad ,(\mathrm{S1})$$

This model does not rely on PWV nor PAT, and is based on a few assumptions, including constant blood viscosity, thin arterial walls, a circular cross-section, and minimal, constant F_{EXT}. Historically, wearable measurements of arterial diameter were not feasible, limiting the model's applicability. Recent advances in ultrasound technology now enable diameter estimation, revitalizing interest in this approach. Most contemporary ultrasound-based systems employ this model, including the most extensively validated system to date[23]. A key limitation is calibration: arterial stiffness is fixed during calibration, and changes in stiffness directly affect pressure readings. This model is our chosen approach.

Bramwell-Hill: Hughes & M-K

The Hughes equation[24] (S2) describes the dependency of the young modulus of a flexible-walled tube E with the pressure p inside it.

$$E = E_0 \cdot e^{\alpha p} \quad ,(\mathrm{S2})$$

The Moens-korteweg equation (S3) is physically grounded and relates the PWV with the tube's wall thickness h, young modulus E, liquid density ρ, and diameter D. It relies on assumptions that are not fully valid for arteries, *e.g.,* a linear response of the artery wall during the entire cardiac cycle.

$$PWV = \sqrt{hE/\rho D} \quad ,(\mathrm{S3})$$

Combining S2 with S3 gives the Bramwell-Hill equation:

$$PWV = \sqrt{\frac{hE_0 e^{\alpha p}}{\rho D}} \quad ,(\mathrm{S4})$$

From which the pressure can be isolated:

$$p = \frac{2}{\alpha}\ln(PWV) + \frac{1}{\alpha}\ln\left(\frac{\rho D}{hE_0}\right) \quad ,(\mathrm{S5})$$

Equation S5 is used in several ways. Often, it is approximated and simplified. Distinct parts of it are assumed to be constant and calibrated for, resulting in structures such as:

$$p = A \cdot \ln(PWV) + B \quad ,(\mathrm{S6}).$$

Fung: Laplace law & M-K

The law of Laplace:

$$E =_{h \ll D} \frac{D^2}{h} \frac{\Delta p}{\Delta D} \quad ,(S7)$$

Can be combined with the Moens-korteweg equation (S3) to give:

$$\Delta p = \rho \cdot p \cdot PWV^2 \frac{\Delta D}{D} \quad ,(S8)$$

Which can then be integrated over a cardiac cycle to give the following relation, that was also found experimentally by Fung[18]:

$$p = p_0 + \rho \cdot PWV^2 \cdot \ln\left(\frac{D}{D_0}\right) \quad ,(S9)$$

These PWV models were used with some success in several systems.

Supplementary Note 4 – Limitations of PPG based systems

PPG sensors are inexpensive, miniature, easy and safe to use in a variety of settings. A PPG sensor operates by transmitting light into the tissue and measuring the intensity of the reflected light. The presence of an artery within the illuminated region modulates the amount of light that is reflected. As the artery expands following systole, it absorbs more light, resulting in a reduction of the reflected signal. By convention, reflectance PPG (rPPG) is expressed in voltage such that a decrease in measured light corresponds to an increase in signal amplitude, thereby aligning its behavior with that of transmission PPG.

This principle results in a signal $r(t)$ that is correlative to the local arterial diameter. PPG sensors can be used for BP estimation in four main ways: (**1**) $r(t)$ is taken as the diameter of the vessel and put into use in different models where the diameter is called for.[16,25] (**2**) A pair or more of PPG sensors are used to give a local PWV estimation[26,27]. (**3**) When measured alongside an electrocardiogram signal $ECG(t)$, the PAT / PTT can be calculated[28]. (**4**) In combination with other modalities[29–32].

Considering the limitations of relying solely on PWV or PAT for blood pressure estimation, systems that incorporate PPG sensors often attempt to use the signal as a surrogate for arterial diameter. However, this approach is flawed - PPG is inherently inaccurate as an estimator of arterial diameter. Numerous factors introduce variability in the amplitude of the PPG signal, affecting both its mean and peak-to-peak values. Yet, information directly related to arterial diameter is contained mostly within these specific components of the signal, highlighting the unreliability of using PPG for this purpose.

To illustrate some of these limitations, we conducted measurements using a commercially available PPG device (SON1303, DFROBOT, China) as shown in Supplementary Fig. 3. The device operates at a single wavelength (532 nm) and is held in position with an adjustable elastic band. Measurement sites included the finger, wrist (above the radial artery), forearm, and elbow. Raw PPG signals were recorded simultaneously with an ECG signal. Analysis of these measurements revealed several phenomena, including:

The shape of the PPG waveform changed significantly for different measurement sites and different contact forces. It was also affected by muscles being activated near the test site. When the test site was the wrist, the position of the hand and fingers was also impactful. Results are in Supplementary Fig. 4.

The delay between the R-wave of the ECG and the peak of the PPG (PAT) depends on how distal the measurement site is. This is expected and it supports the validity of our acquisition method. BP increase was reflected in a reduction in PAT and an increase in PPG amplitude. Results are in Supplementary Fig. 5.

The amplitude was fluctuating. When the test was performed while resting, and the BP level are known to be relatively constant (fluctuating with a standard deviation of 4 mmHg [33]) the amplitude of the PPG signal at the radial artery near the wrist showed significant changes when the PPG sensor was reapplied to the same measurement site, or shifted (±5 mm) transversely to the artery. Any movement of the fingers or wrist had the same unwanted effect.

Supplementary Note 5 – Pulse-echo ultrasound diameter estimation

To illustrate the positional dependency of pulse-echo ultrasound systems based on 1D arrays, we employed the ArtUs USS-2H system with the L12-5N40-A4 transducer (Telemed, Lithuania), as shown in Supplementary Fig. 6. This platform provides full control over transmission patterns (including excitation parameters such as frequency, number of cycles, and drive current) and unrestricted access to raw RF signals (sampled with 16 bits at 40 MS s^{-1}). The transducer is a linear array comprised of 128 elements with a 300 µm pitch (250 µm width and 50 µm kerf), transverse aperture of 4 mm, elevation focus of 20 mm, and a transmission/reception (Tx/Rx) frequency range of 5 – 12 MHz at -6 dB, resulting in a resolution of 100 µm. Supplementary Fig. 7 shows the radial artery imaged with different yaw and pitch values, demonstrating how non-ideal orientation leads to improper evaluation of the artery diameter (if attempted via B-mode imaging). In the following, we analyze the signals from the radial artery when either single elements are separately used, or when all the elements are used together in plane-wave configuration.

Tx/Rx both using individual elements

To illustrate the limitations of PE-US based BP estimation, we emulated the specification of the ultrasound patches described in Ref.[23,34,35] with the Telemed transducer (Supplementary Fig. 8). In these studies, the wearable patch incorporated a 1D array of ultrasound elements, but only a single element is selected to monitor arterial diameter. Accurate diameter estimation requires that the transducer is positioned directly above, square, and parallel to the artery. For smaller arteries, the element spacing must be sufficiently dense, as the optimal measurement point may lie between elements. Even conformal devices, which align naturally with the skin, are not necessarily parallel to the underlying artery, introducing estimation errors of unknown magnitude. While our transducer is a commercial product, rather than custom-made, its pitch and frequency range are comparable to those in the referenced devices. Ref.[23] had a 500 µm pitch (400 µm width and 100 µm kerf), 3 mm transverse aperture, and a Tx/Rx frequency range of 6 – 9 MHz at -6 dB, resulting in a resolution of 231 µm. As in Ref.[23], the elements in our transducer operated sequentially, with each transmitting a single pulse and recording the returning echo. No beamforming, reconstruction, or additional signal processing was applied by the Telemed system. The Tx/Rx bandwidth was set to match that reported in Ref. [23], namely 5.5 - 9.5 MHz at -6 dB. The system`s frame rate was 100 Hz, with each frame containing 64 individual Tx/Rx cycles from the central 64 elements, captured over 0.8 ms, effectively facing the same target. The Telemed platform interleaves a configurable number of B-mode frames between the custom frames to assist with the acquisition.

Supplementary Fig. 8a shows a typical raw B-mode-like image for the radial artery close to the wrist, without beam forming, where each column represents an A-mode signal obtained by a single-element pulse-echo measurement. The figure shows that signals from the artery wall could be identified by elements inside a span of approximately 2 mm. Supplementary Figs. 8b-c show the raw and post-processed M-mode images for elements of different offsets, and Supplementary Fig. 8d shows the corresponding diameter waveforms. Supplementary Fig. 8e also shows the 1D raw pressure signal obtained from a sensor optimally positioned relative to the artery and its post-processed version. Post-processing entailed envelope detection via the Hilbert transform, which was inverted for the posterior artery wall to produce a bi-polar signal, visually comparable to optoacoustic signals. The estimated diameter was then calculated by the difference between the maximum and minimum of the post-processed signal.

The resulting estimates as a function of sensor position (Supplementary Figs. 8f-g) highlight a potential limitation of pulse-echo ultrasound. Even with our probe's smaller pitch of 300 µm, deviation of only one element from the center led to an estimation error close to 200 µm, an order of magnitude larger than clinically acceptable values (Supplementary Note 12). Accordingly, it is possible that translating the transducer by half the pitch would lead to unacceptable estimation errors, even if the central element is used. This error could increase for ultrasound patches which have a pitch of 500 µm, designed for larger arteries.

Planewave Tx / individual element Rx

We study the case in which the transmission is performed with a planewave, while the reception remains with a single element. In this mode of operation, illustrated in Supplementary Figs. 46a-b, the mechanism behind the offset-diameter dependency changes. Supplementary Fig. 46c shows that due to geometrical reasons, when considering echoes only from the blood-vessel wall, the delay between the signals from the two sides of the artery depends on the position of the detector. This result is supported by a k-wave simulation (Supplementary Fig. 46d), and is a known result in scattering theory also studied in optics[36].

An *in vivo* measurement was performed in this mode with the described Telemed platform, using the same frequency settings used in the previous section. Ideally, in this mode, all the transmission elements would need to be excited simultaneously, and all the reception elements would need to measure the resulting echoes in parallel. In practice, the Telemed platform could not operate all the elements in such a fashion at a sufficient frame rate, and this mode was emulated by performing four measurements with a partial coverage of the array, as illustrated in Supplementary Fig. 47c. In each measurement, 24 transmission elements were excited simultaneously and a smaller set of 16 elements measured the echoes in parallel. The four measurements required 0.8 ms for their completion, sufficiently short to neglect tissue motion during this time. The resulting dataset was combined into a single full M-mode frame and 50 such frames were acquired per second, enabling the visualization of the artery pulsation for each reception element. Supplementary Fig. 46e shows 21 concatenated 2-second Raw M-mode snippets from the central 21 elements, spanning ±3 mm around the artery. The figure shows that the delay between the anterior and posterior echoes changes with the offset, in agreement with the simulation.

Tx/Rx both using planewaves

Here, we study the case in which both the transmission and reception operate in plane-wave mode, which may, in principle, be produced by a large single-element transducer. In that case, from a geometrical point of view, the delay between the optoacoustic signals from the anterior and posterior parts of the artery does not depend on the location of the transducer, if it overlaps with the artery (Supplementary Fig. 47a). However, our simulations show that in this case, which lacks any acoustic focusing, the contribution of undesired echoes from the tissue may obfuscate the artery signal.

An *in vivo* measurement of this mode was performed with the described Telemed platform, using the same frequency settings and the same configuration used for Supplementary Fig. 46, but with the exception that signal summation was applied to emulate a larger transducer length. The effect of undesired echoes on the signal quality was studied by emulating single-element transducers with different lengths, ranging from 0.3 to 19.2 mm. The figure shows that as the transducer length increased, which corresponds to poorer beam focusing, the signal was accompanied by progressively stronger echoes from the surrounding tissue, ultimately obscuring the desired artery signal.

Supplementary Note 6 – Optoacoustic patches

Supplementary Table 2 compares the optoacoustic patches developed by X. Gao *et al.* (Ref.[37]), H. Jin *et al.* (Ref.[38]), and K. Djordjev (Ref.[39,40]) to the optoacoustic sensor developed in this work. Several key differences are apparent between our work and previous publications:

In Ref.[37], the Rx acoustic frequencies were in the range of 1.6 - 2.8 MHz at -6 dB, resulting in a relatively poor resolution of 1.1 mm. In addition, signal averaging, required due to the VCSEL low pulse energy level, led to a low framerate of 1 Hz.

In Ref.[38], the Rx frequency range was 3.5 - 10.5 MHz at -6 dB, resulting in a 200 µm resolution. A frame rate of 10 Hz was achieved by using illumination at the wavelength of 532 nm, in which the optical absorption of blood is high, leading to stronger optoacoustic signals. However, because of this high absorption, such a wavelength typically limits imaging to a superficial depth of few millimeters[41].

In Ref.[39,40], the Rx acoustic frequencies spanned 1.25 - 3 MHz at -6 dB, corresponding to a resolution of 1 mm. Despite using VCSEL excitation with performance similar to that of Ref. [37], Ref.[39,40] reported a higher frame rate of 25 Hz, albeit with a thicker, non-flexible device.

Supplementary Note 7 – The optoacoustic signal of a uniform cylinder

The optoacoustic effect (Supplementary Fig. 10a) involves illuminating an optically absorbing object with modulated light, most often laser pulses. When the illuminated object is biological tissue, laser pulses diffuse through the medium until it is fully absorbed. This results in a spatial distribution of energy deposition, referred to as the heating function $H(\mathbf{r})$, which induces a rapid increase in temperature, which is generally on the scale of 1 mK[42]. The rapid temperature-increase causes thermal expansion, which results in an emanating pressure wave. The pressure wave propagates through the medium and can be measured by an acoustic detector positioned on the tissue's surface. The relation between the heating function and the pressure field $p(\mathbf{r}, t)$ is given by the optoacoustic equation[42]:

$$\left(\frac{\partial^2}{\partial t^2} - c^2\nabla^2\right) p(\mathbf{r}, t) = \Gamma \frac{\partial}{\partial t} \delta(t) H(\mathbf{r}) \quad , (\mathrm{S10})$$

Where c is the speed of sound in the medium, and Γ is the Grüneisen coefficient which expresses the efficiency of energy conversion for each material composing the medium. In this form, the laser pulse is assumed to be sufficiently short to be approximated by a delta function in time, $\delta(t)$.

Theoretical optoacoustic signal of a uniform cylindrical source

For the case of an ideal cylindrical source, in which $H(\boldsymbol{r})$ is constant inside the cylinder and is equal to zero outside the cylinder, no simple analytical solution exists. Khan *et al.*[43] derived an expression involving compound integral equations in the frequency domain, whereas Diebold *et al.*[44]*,* found the following integral expression:

$$P(\hat{\tau}) \propto \begin{cases} 0 & \hat{\tau} < 0 \\ \displaystyle\int_{-\hat{\tau}}^{0} \frac{(\eta + 1)}{\sqrt{\hat{\tau} + \eta}\sqrt{1 - (\eta + 1)^2}} d\eta & 0 < \hat{\tau} < 2 \\ \displaystyle\int_{-2}^{0} \frac{(\eta + 1)}{\sqrt{\hat{\tau} + \eta}\sqrt{1 - (\eta + 1)^2}} d\eta & \hat{\tau} > 2 \end{cases} \quad , (\mathrm{S11})$$

In equation S11, $\hat{\tau}$ is normalized time, and the cylinder is assumed to have a diameter of 2. By scaling and shifting of $\hat{\tau}$, any diameter can be described for any sensor distance. In Supplementary Fig. 10b, an evaluation of this expression is shown. The diameter of the uniform cylinder is thus easy to extract from the time-resolved pressure signal based on its extremum points.

Supplementary Note 8 – Optoacoustic simulations

To support system design and validate key aspects of its performance, we developed a comprehensive optoacoustic simulation environment. The simulation pipeline proceeds as follows: (**1**) The anatomical configuration of the vessels is defined, including dynamic properties such as arterial pulsation and potential artery-vein interactions. (**2**) The illumination profile is specified, together with the excitation wavelength and all wavelength-dependent optical parameters of the tissue, including oxygen saturation and melanin concentration. The MCmatlab[45] engine is then used to compute the spatially resolved heating function. (**3**) The acoustic detection parameters are defined, including bandwidth, acceptance angle, element size, position, and orientation, and the k-Wave[2] package is used to model the propagation of the resulting acoustic waves. Signal-to-noise ratio (SNR) can be adjusted by incorporating sources of noise, including shot noise, electronic noise, and stochastic absorption noise arising from hemoglobin presence in the tissue. Acoustic heterogeneities (variations in speed of sound, density, and frequency-dependent attenuation) are incorporated through parameterized models to ensure realistic simulations. Supplementary Table 3 summarizes the acoustic properties used in the k-wave simulation.

Diffuse optics simulations

A 3D diffuse-optics Monte-Carlo simulation was performed using the MCmatlab[45] engine for the illumination geometry of the optoacoustic detector. The diffusive medium included a blood vessel with depth and diameter that are set as needed. The wavelength-dependent optical parameters $(\mu_\mathrm{a}, \mu_\mathrm{s}, g)$ of all tissue types (dermis, epidermis, vessel wall and blood) were used for the wavelength of 800 nm[46]. Blood oxygen saturation was considered, as well as blood and melanin presence at the epidermis. The effective absorption coefficient for arterial blood (98% O2 saturation) at 800 nm is $\mu_\mathrm{eff} = 1.376\ \mathrm{mm}^{-1}$[47,48], indicating that the posterior end of a vessel is only excited by photons that had scattered back, and not by photons that travel through the whole vessel. The resulting heating function is presented in Supplementary Fig. 11, showing that the blood vessel is well illuminated in this configuration.

Phantom mimicking simulations with SPADE

A series of simulations were conducted to replicate the conditions of the phantom experiments performed with SPADE. At a depth of 4 mm, five cylindrical absorbers with diameters matching those of the physical phantoms (0.75 - 3.125 mm) were simulated. The corresponding heating functions were derived from simulations of the exact illumination scheme used in the experiments. The sensor geometry and bandwidth of SPADE were faithfully modelled, including the effective bandwidth reduction associated with non-normal incidence angles (pitch > 0°). The correct acoustic properties of the heterogeneous medium (Supplementary Table 3) were included in the simulation. Simulations were performed for yaw angles ranging from 0° to 30° and with lateral offsets of up to ±10 mm. Noise was excluded to isolate systematic effects. Diameter estimation was carried out using the same algorithm employed for *in vivo* data analysis (Methods). When results were averaged across all diameters, offsets, and orientations, the standard deviation of the diameter estimation error was 3 µm. Particularly, the choice of yaw angle induced STD changes smaller than 0.5 µm throughout.

Supplementary Note 9 – Safety

Our study involving human participants was approved by the Technion Helsinki committee. Safety considerations addressed both ultrasound exposure limits and laser safety requirements. The use of a Class IV excitation laser necessitated strict adherence to safety protocols; both the participant and the operator wore certified protective goggles (PROTECT Laserschutz, Germany). Future iterations of our system are expected to relax this requirement by incorporating an interlock mechanism that activates the excitation laser only when proper contact with the skin is detected. Under these conditions, when the fiber bundle is in direct contact with the skin, the need for protective eyewear is eliminated.

The illumination bundle was designed, simulated, and experimentally characterized to comply with the requirements of the ANSI Z136.1 laser safety standard[49]. This standard specifies multiple maximum permissible exposure (MPE) limits, all of which were carefully evaluated and enforced. The most restrictive limit for a laser operating at 100 Hz is on the fluence rate: MPE = 316.8 mW cm^{-2}. Since the illumination area was 1.56 cm^2, the MPE corresponded to a total laser power of 494 mW. In our system, as in most optoacoustic imaging (OAI) systems, the signal-to-noise ratio (SNR) scales with pulse energy. Consequently, the system was intentionally designed to operate at approximately 99 % of the maximum permissible exposure (MPE) specified by ANSI Z136.1. This compliance was verified through an independent assessment by a certified laser safety inspector. At the skin surface, the delivered energy was measured (PM100USB and S425C, Thorlabs, USA) to be 4.9 mJ per pulse, corresponding to an average power of 490 mW. It is worth noting that ANSI Z136.1 incorporates a substantial safety margin: for slight erythema (local skin redness) to occur, the MPE must typically be exceeded by a factor of ×20 for light skin and ×10 for dark skin[50–52].

Supplementary Note 10 – SPADE characterization (NEP, BW, PSF)

The signal to noise ratio (SNR) of the received optoacoustic signal is determined by several factors:

$$SNR \propto \frac{P_{\mathrm{laser}}\sqrt{N_{\mathrm{avg}}}}{NEP} \quad ,(\mathrm{S12})$$

where P_{laser} is the excitation laser power, N_{avg} is the number of averages, and NEP is the noise-equivalent pressure of the sensor. In our system, laser power is constrained by safety standards, and no averaging ($N_{\mathrm{avg}} = 1$) is used to maintain a 100 Hz pulse repetition rate. This rate allows up to 33 BP estimates per cardiac cycle at heart rate of 180 BPM. Consequently, NEP becomes the dominant factor governing sensor performance.

Noise Equivalent Pressure (NEP)

The NEP of SPADE was evaluated using the system shown in Supplementary Fig. 13a, in which an ultrasound transducer (0.5", 5 MHz central frequency, Evident Scientific, Japan), pre-calibrated in a hydrophone measurement (NH1000, Precision Acoustics, UK), created an acoustic beam with a peak-to-peak pressure of $P_{\mathrm{PTP}} = 1.1\ \mathrm{kPa}$ at the position of the SPADE. The NEP over the entire bandwidth was then calculated from the measurement using the following equation:

$$NEP_{[\mathrm{Pa}]} = \frac{P_{\mathrm{PTP}}}{SNR} \quad ,(\mathrm{S13})$$

Where SNR is the linear signal to noise ratio of the SPADE in the measurement. To quantify the spectral density of the NEP, the following equation was used:

$$NEP_{[\mathrm{Pa\ Hz^{-1/2}}]} = \frac{NEP_{[\mathrm{Pa}]}}{\sqrt{BW}} \quad ,(\mathrm{S14})$$

Where BW is the -6 dB bandwidth measurement. Since SPADE is generally governed by optical shot noise[53], the NEP depends on the optical power of the interrogation laser. Supplementary Fig. 13c shows that for interrogation laser power of 14 mW, a NEP of 2 mPa $\mathrm{Hz}^{-1/2}$ is reached.

Bandwidth (BW)

BW evaluation was performed using the methodology of Ref.[53]. Briefly, a wideband optoacoustic point source was generated by focusing a 1 ns FHWM laser pulse (WAVEGUARD-D, Optogama, Lithuania) to a spot 5 μm in diameter on the surface of a glass slide coated with thin layer of ink (model 750, Edding, Germany). The SPADE was positioned 0.9 mm above the coated surface to avoid the attenuation of the signal's high frequencies in the water. The beam was scanned on the surface to find the position with minimum delay, which corresponds to an optoacoustic source the coincides with the center of the SPADE.

Point Spread Function (PSF)

To measure the PSF of SPADE, the optical beam was scanned over an area of 5 × 5 mm on the coated surface with a step size of 4 μm, where for each position, the resulting acoustic signal was recorded by the SPADE (Supplementary Fig. 13b). The universal back-projection algorithm[41] was applied on the measured signals to produce a reconstruction of the optoacoustic point source shown in Fig. 3e. Since the source had a diameter of merely 5 μm, smaller than the resolution of the system, it may be considered as a point source, and its reconstruction effectively represents the PSF of the system.

Supplementary Note 11 – Phantom experiments with SPADE

Phantoms fabrication

The phantoms (Supplementary Fig. 14) were designed to mimic the radial artery in both size and depth, with optical and acoustic properties chosen to approximate biological tissue. Agar (Merck Millipore, Germany) served as the surrounding medium, with added cream[54–56] to achieve physiologically relevant optical scattering. For every 100 ml of 2% agar solution, 6 ml of 38% fat cream were added, to theoretically give $\mu_s' \cong 2\ \text{mm}^{-1}$ [57]. Notably, the Agar`s frequency dependent acoustic attenuation resembled that of tissue, as shown in Supplementary Table 3. The vessels were modelled using flexible, transparent PVC tubing (medical catheters, Stericom, UK) with inner and outer diameters corresponding to the radial artery. The tubes were filled with donated human blood and sealed at both ends with hot-melt adhesive, then positioned in 3D-printed mounts before embedding them in the agar mixture, which was allowed to solidify. The blood was chilled prior to use, ensuring near-complete oxygen saturation[58] and thus reproducing the optical properties of arterial blood. The phantom set included tubes with inner diameters of 0.85, 0.98, 1.23, 1.87, 2.46, and 3.05 mm. It is important to note that while the tubes shape was indeed cylindrical, small variations in their inner and outer diameters along their length are possible, which may affect the optoacoustic signal generated in them.

Phantoms diameter estimation with SPADE

In the phantom experiment, the SPADE was vertically integrated with the fiber-bundle in a hand-held configuration to simplify handling (Supplementary Fig. 14a). This constructed optoacoustic probe was held by a servo-controlled stage (Physik Instrumente, Germany) and submerged in deionized water together with the phantom. For each phantom size, the device was positioned at near contact with the agar, parallel, centered, and true to the vessel. The stage moved the sensor across the surface with high precision. 200 positions were captured with a pitch of 100 μm. Supplementary Fig. 15 shows sinograms and their reconstructions, alongside 1D optoacoustic signals measured by the SPADE-based optoacoustic probe and by a piezoelectric probe for comparison (Supplementary Fig. 19b). The increase in performance and signal fidelity compared to piezoelectric sensors is apparent and the pressure waveform is in excellent agreement with the simulated waveform.

In addition to scanning in the optimal orientation, a pitch of 10°, and yaws of 10°, 20°, and 30° were used (Supplementary Fig. 17). For diameter estimation, the acquired signals underwent minimal processing, including interpolation, high-pass filtering at 100 kHz, median profile subtraction, and Savitzky-Golay[59] filtering for edge-preserving noise suppression. Diameter estimation was then performed using the inflection-point method (Methods, Supplementary Fig. 40). The resulting mean STD of approximately 10 μm, averaged across all diameters, offsets, and orientations, demonstrates excellent accuracy and confirms that the measurement is fundamentally independent of sensor offset, vessel diameter, sensor distance, and system bandwidth. The operational range was limited to ±10 mm primarily because the illumination source moved with the sensor, causing the illumination field to drift. With broader illumination, the sensor's acceptance angle would permit an even larger operational range. Results are presented in Supplementary Fig. 17, demonstrating that the yaw and pitch angles did not have a meaningful effect on the consistency of the diameter estimation within the linear scan.

Supplementary Note 12 – Error propagation

In the following, we analyze how errors in the measured diameter are propagated to errors in the estimated pressure, based on Hayashi's model (Supplementary Note 3):

$$p(t) = p_{\mathrm{dia}} \cdot \exp\left[\beta\left(\frac{D(t)^2}{D_{\mathrm{dia}}^2} - 1\right)\right] \quad ,(\mathrm{S15})$$

where the calibration parameter β is given by:

$$\beta = \frac{\ln\left(\frac{p_{\mathrm{sys}}}{p_{\mathrm{dia}}}\right)}{\left(\frac{D_{\mathrm{sys}}}{D_{\mathrm{dia}}}\right)^2 - 1} \quad ,(\mathrm{S16})$$

To convert errors in the diameter, D, to pressure, p, the following derivative is calculated:

$$\frac{dp}{dD} = p_{\mathrm{dia}} \cdot \exp\left[\beta\left(\frac{D^2}{D_{\mathrm{dia}}^2} - 1\right)\right] \cdot \beta \frac{2D}{D_{\mathrm{dia}}^2} = \frac{2p\beta D}{D_{\mathrm{dia}}^2} [\mathrm{mmHg\ mm^{-1}}] \quad ,(S17)$$

This leads to the following relationship between the standard deviations of D and p:

$$\sigma_p = \frac{2p\beta D}{D_{\mathrm{dia}}^2} \sigma_D \quad ,(\mathrm{S18})$$

To obtain specific values for σ_p, the following parameters were substituted:

$$p_{\mathrm{dia}} = 80\ \mathrm{mmHg}, p_{\mathrm{sys}} = 130\ \mathrm{mmHg}, D_{\mathrm{dia}} = 1.5\ \mathrm{mm}, D_{\mathrm{sys}} = 1.7\ \mathrm{mm}.$$

These parameters lead to a β value of:

$$\beta = \frac{\ln(130/80)}{\left(\frac{1.7}{1.5}\right)^2 - 1} = 1.7069 \quad ,(\mathrm{S19})$$

and an estimation model of:

$$p(D) = 80 \cdot \exp\left[1.7069 \cdot \left(\left(\frac{D}{1.5}\right)^2 - 1\right)\right] \quad ,(\mathrm{S20})$$

with an STD relationship of:

$$\sigma_{\mathrm{SBP}}[\mathrm{mmHg}] \cong \frac{\sigma_D[\mu\mathrm{m}]}{3} \quad ,(\mathrm{S21})$$

These results are further summarized in Supplementary Fig. 18.

Supplementary Note 13 – Piezoelectric optoacoustic imager

To compare the performance of our SPADE-based optoacoustic sensor to a comparable device based on piezoelectric technology for ultrasound detection, a custom-made handheld imaging transducer was used[60] (Supplementary Fig. 19), originally designed for imaging superficial blood vessels at depths up to 1 cm. The optoacoustic transducer employed a bundle-based (CeramOptec, Germany) illumination scheme with a linear array of 64 piezoelectric elements (IMASONIC, France), connected to the same OPO laser used in the SPADE-based optoacoustic sensor, enabling real-time (100 Hz) imaging of the cross section of blood vessels.

The piezoelectric elements had a pitch of 200 μm (160 μm width and 40 μm kerf), element length of 5 mm, no acoustic lens, and a Rx frequency range of 1.5 - 6.5 MHz at -6 dB, resulting in a resolution of 278 μm. The data acquisition was performed with 12 bits at 40 MS s^{-1} by the Legion ADC (PhotoSound, USA), which includes low-noise amplifiers to maximize SNR. The NEP of the piezoelectric transducer was measured to be 0.3 mPa $Hz^{-1/2}$, in general agreement with other electro-mechanical detectors[61–63] which achieved NEPs in the range of 0.5 - 2 mPa $Hz^{-1/2}$. While the piezoelectric detector used in this work had a superior NEP, when calculating the NEP × area, its performance is close to two orders of magnitude worse than those of the SPADE used in our work, as shown in Supplementary Table 4.

In vivo imaging with the piezoelectric transducer

Supplementary Fig. 20 shows *in vivo* imaging of the radial artery performed for several non-ideal pitch and yaw angles. The figure shows that changing the orientation of the transducer deforms the reconstructed image and hinders the capability of estimating the artery diameter.

Phantom imaging with the piezoelectric transducer

Supplementary Fig. 15 shows the raw signals obtained by the piezoelectric transducer when scanned above the tissue mimicking phantom (Supplementary Note 11) and the resulting reconstruction. The results are compared to those produced by the SPADE based optoacoustic sensor, which obtained a sharper reconstruction and a wider detection span.

Phantoms diameter estimation using the piezoelectric transducer

The described set of phantoms was measured with the piezoelectric transducer. Offset control was done by selecting an element from the 1D array. The results are shown in Supplementary Fig. 22. Diameter estimation was only accurate for pitch < 5°, yaw < 5° and offset < ± 5 mm. This range reduction is due to the width and length of the PE elements and was received despite the somewhat better illumination profile. Withing this reduced range, the average STD across the set was 20 μm.

Supplementary Note 14 – Inducing BP changes

Evaluating a cuffless BP estimation system in accordance with IEEE 1708-2019 poses significant challenges, particularly in achieving the required coverage across a wide range of BP values. The standard does not prescribe how to safely induce such BP changes, and conventional clinical interventions (e.g., pharmacological vasopressors or vasodilators) are considered unsafe or unethical in clinical BP studies, as the subject stands nothing to gain from the administration of such medications.

Physical exercise is frequently used to elevate BP but was deemed unsuitable for our protocol. Exercise introduces variability in the magnitude and temporal decay rate of BP changes across subjects and would require repeated re-application of the NOVA finger cuff, ECG electrodes, and optoacoustic probe, potentially compromising measurement reproducibility. Moreover, the standard mandates reference measurements with a conventional BP cuff, which can only be taken intermittently due to vascular occlusion, introducing potential systematic error relative to continuous device readings (Supplementary Fig. 44).

Inducing reductions in BP is even more difficult. As recommended by measurement guidelines[2], subjects begin in a calm, resting state, leaving little room for further reduction. Through experimentation, we have found that techniques such as paced breathing, relaxation exercises, or the Valsalva maneuver did not yield consistently sustained BP decreases over the minutes required by the IEEE 1708 standard.

Consequently, we restricted our approach to two simple, conservative techniques that satisfy three key criteria: (**1**) induce changes that are stable over several minutes, (**2**) allow measurement gear to remain in place, and (**3**) are feasible for all subjects. These techniques are described below.

Isometric "Seated-Core-Hold" position

Standard BP measurement guidelines[2] recommend that subjects sit with back support, feet flat on the floor, legs uncrossed, and remain relaxed for at least 5 minutes before measurement. We observed that systematically violating these conditions by adopting an isometric "seated-core-hold" position produces a reproducible BP elevation. In this position, the subject leans forward to remove back support, extends and crosses the legs, and suspends them off the floor, thereby engaging core and leg muscles. The resulting BP increase is stable if the position is maintained (Supplementary Fig. 44c).

Naturally variable subjects

Day-to-day variability in resting BP can be leveraged to expand the dynamic range of reference measurements. This variability is known to increase with age, body mass index (BMI), and comorbidities, particularly renal disease, which is associated with pronounced spontaneous BP fluctuations. For such subjects, repeated measurements on separate days yield distinct baseline BP values without the need for external intervention. This approach will bear more weight in an upcoming, more extensive, clinical study.

Supplementary Note 15 – Grating couplers

The selection of fiber-to-chip coupling strategy is important for minimizing insertion loss and ensuring robustness against external forces, which is essential for wearable applications. In our design, we chose to use grating couplers, rather than edge couplers, for several reasons: Grating couplers enable bonding the fibers directly on the chip, leading to a simpler procedure with fewer degrees of freedom and to a more robust structure. If edge couplers were to be chosen, both the fibers and chip would need to be bonded on the glass substrate, which is less rigid than the silicon chip, making the entire structure potentially more susceptible to bending. Grating couplers also offer the advantage of rapid inspection of all the structures without any processing steps, whereas edge couplers require that the chip is cut and side polished to enable inspection.

The structure for the grating couplers used in this work was taken from the Ligentec (Switzerland) development kit. These grating couplers were produced in a single etching step with a feature size of 300 nm and without any additional structures. Standard uniform grating couplers in platforms are limited by two primary loss mechanisms: suboptimal directionality (power radiated toward the substrate) and poor mode-overlap. In the following, several advanced strategies for mitigating these issues are discussed.

Bottom mirrors

In a conventional grating coupler, approximately 40% of the coupled optical power is lost to substrate radiation. This directionality penalty can be largely circumvented by introducing a highly reflective layer, such as a deposited metal mirror or a multi-layer Distributed Bragg Reflector beneath the waveguide. This reflects the downward-diffracted optical field, allowing it to constructively interfere with the upward-propagating mode. The primary limitation of this method is its fundamental incompatibility with standard CMOS-compatible foundry flows. Typical insertion losses of 1.2 dB per coupler were demontrated[64] in Si_3N_4.

Two-level etch

To address the poor mode-overlap inherent to single-etch periodic gratings, a two-level etch can be implemented to create an asymmetric profile. This localized structural tuning shapes the diffracted beam into a near-ideal Gaussian profile, enhancing the mode-overlap integral. The fabrication of two-level, apodized gratings require multiple lithography and dry-etching cycles. The resulting optical performance is sensitive to misalignment between the consecutive etch steps. Furthermore, the localized effective index is highly dependent on precise etch depths, making uniform fabrication technically demanding. Insertion losses as low as 0.45 dB per coupler were demontrated[65] in Si_3N_4.

Inverse design

Inverse design employs advanced computational algorithms to synthesize device architectures based entirely on target performance metrics. By defining the input waveguide mode and the desired output fiber mode, the algorithm iteratively explores the physical parameter space to generate free-form, aperiodic structures. This approach can simultaneously optimize maximum mode overlap, high directionality, and expanded operational bandwidth, frequently achieving insertion losses below 1 dB without requiring substrate modification. The primary drawbacks are the significant computational overhead required and the fabrication fragility of the resulting devices. Typical insertion losses of 1.82 dB per coupler were demontrated[66] in Si_3N_4.

Supplementary Tables

Supplementary Table 1 – Comparing cuffless blood pressure estimation standards with this work

<table>
<tr><th colspan="2">Parameter</th><th colspan="4">IEEE 1708-2019</th><th colspan="2">ISO 81060-3</th><th>This work</th></tr>
<tr><td colspan="2">Reference method</td><td colspan="4">Two observers are needed as part of the validation team. Validation is done against two mercury-based manual sphygmomanometers. The average of the two is taken, given they are no more than 4 mmHg apart.</td><td colspan="2">Invasive A-line only.</td><td>A Finapres NOVA device that is calibrated with an automated brachial cuff and gives continuous finger and brachial BP waveforms, from which we generate the intermediate radial waveform.</td></tr>
<tr><td colspan="2">Reference timing</td><td colspan="4">Perform simultaneous reading if possible. For successive readings, wait 30 - 60 seconds after the reference cuff due to vascular occlusion.</td><td colspan="2">Simultaneous only. Occlusion not considered.</td><td>A finger cuff is used simultaneously without occlusion.</td></tr>
<tr><td colspan="2">Beat-to-beat</td><td colspan="4">Take a 20 second average and compare SBP & DBP.</td><td colspan="2">Average SBP & DBP over the system-specific segment duration, which may result in no averaging at all.</td><td>Compare SBP & DBP from both the individual cardiac cycles, and the averages of 10 seconds segments.</td></tr>
<tr><td colspan="2">Full waveform</td><td colspan="6">Not addressing.</td><td>Qualitative comparison.</td></tr>
<tr><td colspan="2">Time after calibration</td><td colspan="6">Test as much as claimed is possible.</td><td>3 months</td></tr>
<tr><td colspan="2">Number of subjects</td><td colspan="4">20 in phase 1
65 in phase 2</td><td colspan="2">30 – 278, depending on set correlation.</td><td>2 subjects</td></tr>
<tr><td rowspan="3">BP changes from calibration</td><td>Min %</td><td>13.6</td><td>34.1</td><td>34.1</td><td>13.6</td><td>30</td><td>30</td><td rowspan="3">Supplementary Fig. 45.</td></tr>
<tr><td>$SBP_{[mmHg]}$</td><td>(-30,-15)</td><td>(-15,0)</td><td>(0,+15)</td><td>(+15,+30)</td><td>< -15</td><td>> +15</td></tr>
<tr><td>$DBP_{[mmHg]}$</td><td>(-20,-10)</td><td>(-10,0)</td><td>(0,+10)</td><td>(+10,+30)</td><td>< -10</td><td>> +10</td></tr>
<tr><td colspan="2">Metrics</td><td colspan="4">MAD (Mean Absolute Difference)</td><td colspan="2">MD (Mean of Difference)
SD_{corr} (corrected experimental standard deviation)</td><td>MAD</td></tr>
<tr><td colspan="2">Position</td><td colspan="4">The default testing position is sitting upright; if the device is intended for supine, prone, or standing use, each position must be validated separately with the full subject distribution and BP range, and positions cannot be mixed in one validation set.</td><td colspan="2">Check if different positions affect performance. If they do, use a single position throughout (supine / sitting).</td><td>Sitting and seated-core-hold.</td></tr>
<tr><td colspan="2">Grading</td><td colspan="4">Grade A device
MAD ≤ 5 mmHg</td><td colspan="2">Pass
$MD \pm SD_{corr} \leq 6 \pm 10$ mmHg</td><td>MAD_{SYS} = 4.7 mmHg
MAD_{DIA} = 2.6 mmHg</td></tr>
<tr><td colspan="2">Motion artifacts and dynamics testing</td><td colspan="4">Motion tests and dynamic tests are discouraged, as reference devices are unsuitable for them.</td><td colspan="2">Not addressing.</td><td>No motion, as it is currently clinically unjustified.</td></tr>
<tr><td colspan="2">Population</td><td colspan="6">Subject population must be following minimal participation percentages with regards to subject age, sex, and blood pressure levels.</td><td>29M – slightly hypertensive
42F – slightly hypotensive</td></tr>
</table>

Supplementary Table 2 – Comparing wearable optoacoustic platforms with this work

Parameter	X. Gao 2022[37]	H. Jin 2023[38]	K. Djordjev 2025[39,40]	This work
**Excitation source**	VCSEL 2D array, Ace Photonics, China	OPO, Opotek, UK	2 VCSELs	OPO, InnoLas, Germany
**Excitation wavelength**	850 nm	532 nm	904 nm	800 nm
**Excitation pulse energy at the skin**	0.192 mJ	1.3 mJ	0.012 mJ †	4.9 mJ
**Repetition rate**	3 kHz	10 Hz	3.2 kHz †	100 Hz
**Excitation pulse FWHM**<br>**Tx* BW limit**<br>**Tx* Resolution limit**	200 ns<br>2.2 MHz<br>630 µm	7 ns<br>63 MHz<br>22 µm	200 ns<br>2.2 MHz<br>630 µm	7 ns<br>63 MHz<br>22 µm
**Light delivery**	On device	25 × 100 µm fibers.<br>NA = 0.22	On device<br>2 lightguides	Ribbon fiber bundle with 8,350 × 50 µm Borosilicate fibers.<br>NA = 0.55
**Light deflection**	-	Polished & silver coated fibers with microlens array	-	Gold coated polished aluminum mirror
**Averaging**	× 3,000	none	× 128 †	none
**FPS**	1	10	25 †	100
**Conformal**	Yes	Yes	No	No
**Rx technology**<br>**-6 dB Rx frequency range**<br>**Rx Resolution limit**	Piezoelectric<br>1.6 – 2.8 MHz<br>1.1 mm	Piezoelectric<br>3.5 – 10.5 MHz<br>200 µm	Piezoelectric<br>1.25 – 3 MHz<br>1 mm	SPADE<br>0 – 125 MHz<br>11 µm
**Tx*/Rx Resolution limit**	1.1 mm	200 µm	1 mm	22 µm
***In vivo* -6 dB BW**	Not reported	Not reported	Not reported	28 MHz
**Rx array**	2D, 13 × 13	2D, 6 × 6	1D, 6 elements	Single sensor
**Acquisition**	Verasonics, USA<br>multi-channel	SonixDAQ, USA<br>multi-channel	Custom<br>multi-channel	PicoScope, UK<br>Single channel

Tx* refers to the properties of the US signal generated in the tissue, by the excitation light pulse, via the optoacoustic effect.

† Values derived from characterization data reported in Ref.[39,40]: pulse energy calculated from 40 mW average optical power at lightguide exit divided by 3.2 kHz repetition rate; averaging count calculated from repetition rate divided by frame rate. The authors report frame rates of 25 - 100 Hz but do not report in vivo values, visual inspection of the diameter waveform suggests 25 FPS.

Supplementary Table 3 – Acoustic properties of simulation and phantom materials

<table>
<tr><th>Parameter</th><th>Blood[14]</th><th>Blood Vessel wall[14]</th><th>(Epi)Dermis[14]</th><th>PVC[67]</th><th>Water[14]</th><th>AGAR 2%[68]</th></tr>
<tr><td>c_0 $[\mathrm{m\ s^{-1}}]$</td><td>1580</td><td>1570</td><td>1540</td><td>1398</td><td>1525</td><td>1564</td></tr>
<tr><td>ρ $[\mathrm{Kg\ m^{-3}}]$</td><td>1060</td><td>1070</td><td>1100</td><td>1370</td><td>993</td><td>1020[69]</td></tr>
<tr><td>α_{coeff} $[\mathrm{dB\ MHz^{-\alpha_{power}}\ cm^{-1}}]$</td><td>0.13</td><td colspan="2">0.75[70]</td><td>0.353</td><td>0.002</td><td>0.8268</td></tr>
<tr><td>α_{power}</td><td colspan="3">1.2</td><td>1.578</td><td>2</td><td>1</td></tr>
</table>

Supplementary Table 4 – Comparing a piezoelectric sensor to SPADE

Parameter	SPADE (this work)	Piezoelectric [60]
**-6 dB Rx frequency range**	0 – 125 MHz	1.5 - 6.5 MHz
**Effective sensor width**	18 µm	160 µm
**Effective sensor length**	83 µm	5 mm
**Acceptance angle**	> 60°	< 20°
**NEP**	2 mPa $Hz^{-1/2}$	0.3 mPa $Hz^{-1/2}$
**NEP × area**	3×10^{-12} Pa m^2 $Hz^{-1/2}$	240×10^{-12} Pa m^2 $Hz^{-1/2}$

Supplementary Table 5 – Gaussian kernel relations

	$\boldsymbol{\sigma_{STD}}$	$\mathbf{BW_{-6dB}}$
$\mathbf{BW_{-6dB}}$	$\sigma_{STD} = \frac{\sqrt{\ln 2}}{\sqrt{2}\pi \cdot BW_{-6dB}} \cong \frac{0.1874}{BW_{-6dB}}$ (S22)	-
$\mathbf{t_{rise-step}}$ *	$\sigma_{STD} = \frac{t_{rise-step}}{2\sqrt{2}\,\mathrm{erfinv}\,0.8} \cong \frac{t_{rise-step}}{2.563}$ (S23)	$BW_{-6dB} = \frac{2\sqrt{\ln 2}\,\mathrm{erfinv}\,0.8}{\pi \cdot t_{rise-step}} \cong \frac{0.48}{t_{rise-step}}$ (S25)
$\mathbf{t_{rise-pulse}}$ *	$\sigma_{STD} = \frac{t_{rise-pulse}}{\sqrt{2\ln 10} - \sqrt{-2\ln 0.9}} \cong \frac{t_{rise-pulse}}{1.6869}$ (S24)	$BW_{-6dB} = \frac{\sqrt{\ln 2}\left(\sqrt{2\ln 10} - \sqrt{-2\ln 0.9}\right)}{\sqrt{2}\pi \cdot t_{rise-pulse}} \cong \frac{0.316}{t_{rise-pulse}}$ (S26)

* The definitions are illustrated in Supplementary Fig. 42b.

Supplementary Figures

Supplementary Figure 1 – Properties of an artery

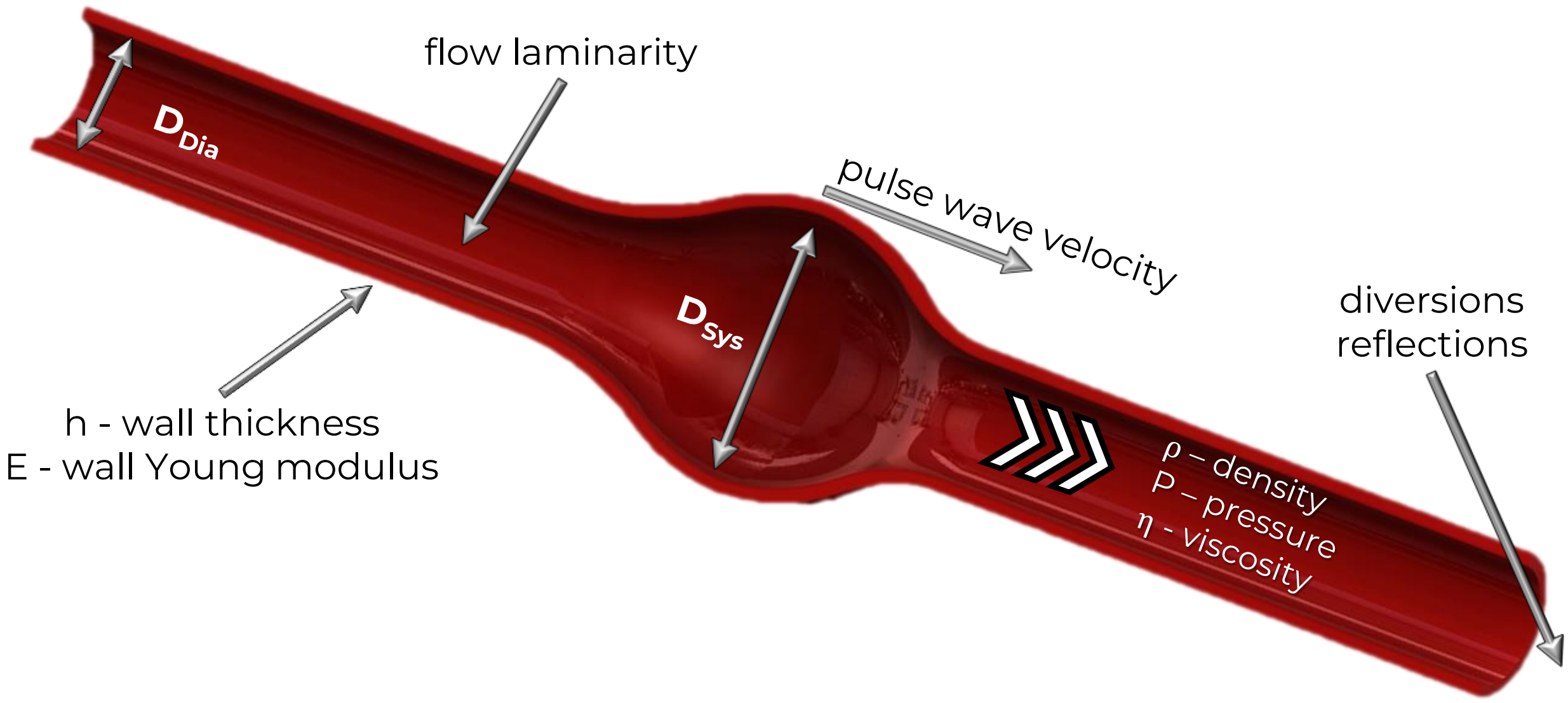

Supplementary Figure 1 – **Properties of an artery**. A partial list of the mechanical properties of an artery section, most of which are relevant to estimation models.

Supplementary Figure 2 – Sphygmomanometery principle of operation

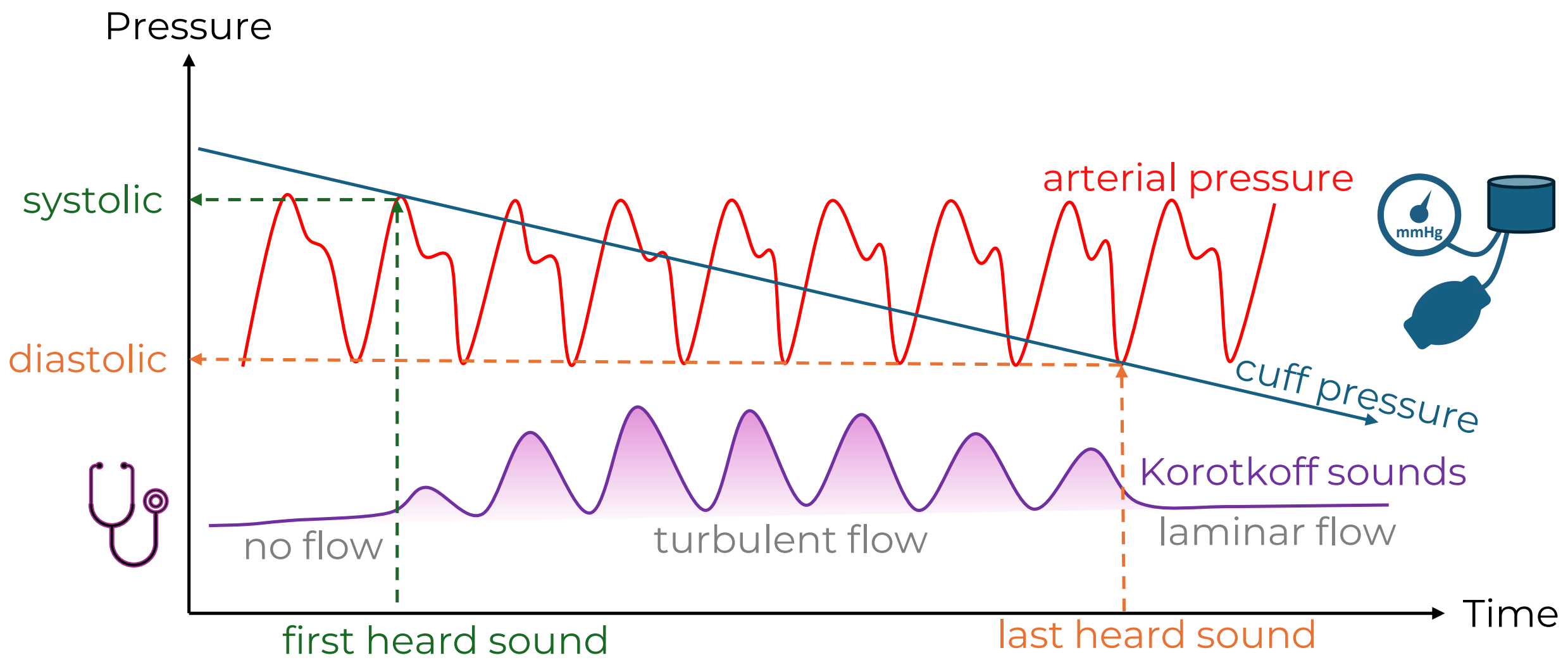

Supplementary Figure 2 – **Sphygmomanometery principle of operation**. The cuff pressure (blue) is gradually released until it falls below the arterial pressure (red), allowing intermittent blood flow that produces audible Korotkoff sounds (purple). The first sound heard corresponds to the systolic pressure, and the last to the diastolic pressure. The transition from turbulent to laminar flow marks the return to uninterrupted blood flow and the end of the measurement.

Supplementary Figure 3 – PPG and PWV

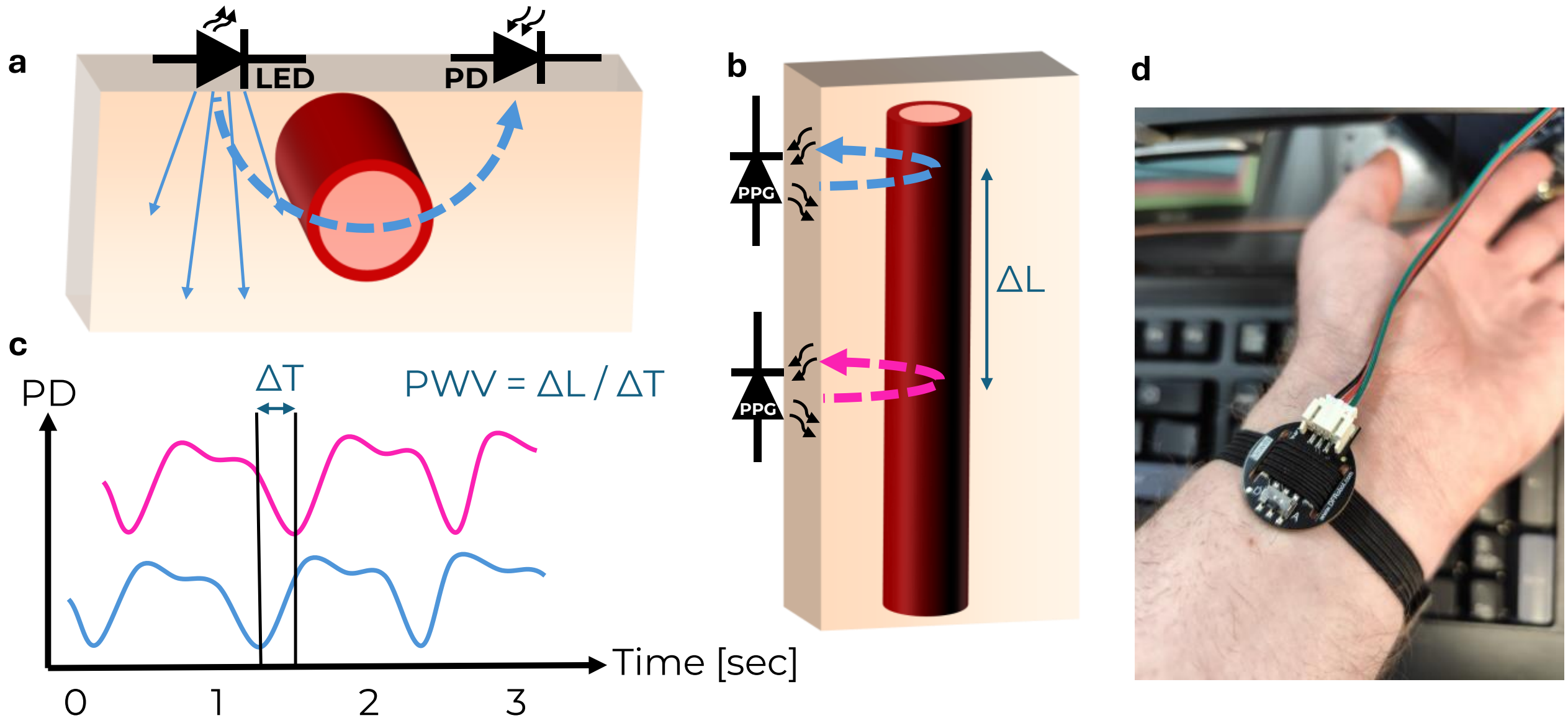

Supplementary Figure 3 - **PPG and PWV**. **a** The operation principle of reflection PPG (photoplethysmography): A considerable portion of the light emitted by the LED (light emitting diode) and detected by PD (Photodiode) passes through the artery, where it undergoes strong absorption due to the high blood concentration. Changes in the local blood volume due to the artery pulsation modulates the detected intensity. **b-c** The method of using two PPG sites to measure local PWV (pulse wave velocity) via the delay in the PPG signals. **d** Our PPG sensor applied to the radial artery of a 29-year-old male to demonstrate its position sensitivity.

Supplementary Figure 4 – PPG` s susceptibility to inert changes

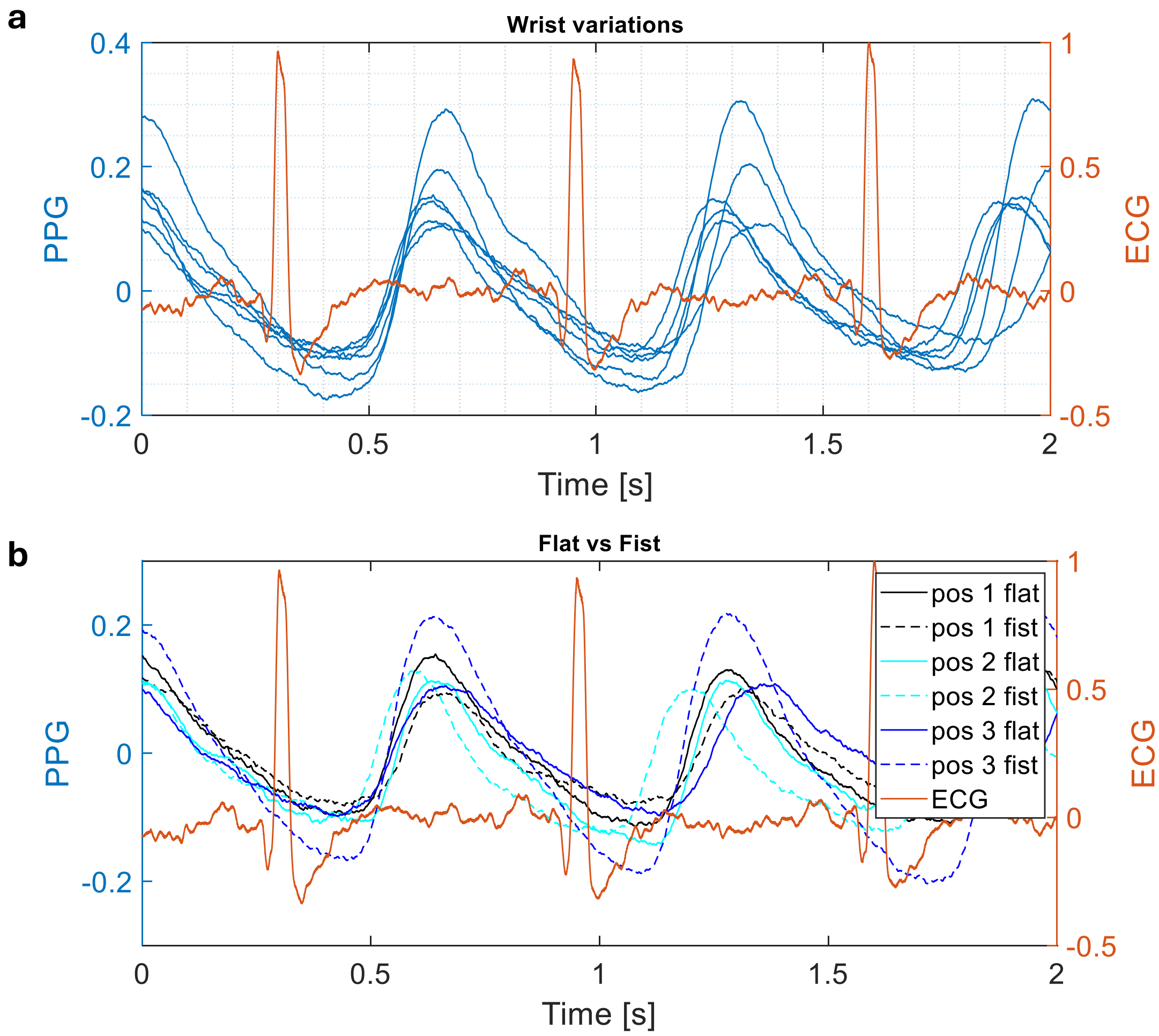

Supplementary Figure 4 - **PPG`s susceptibility to inert changes**. **a** The ECG (electrocardiogram) signal alongside PPG (photoplethysmogram) signals taken at slightly different positions, all near the wrist of a 29-year-old male. Changes in amplitude, shape, and PAT (pulse arrival time) are apparent. **b** The ECG signal alongside PPG signals from three longitudinal positions. For each, the difference between a flat hand and a fist is shown. Changes in amplitude, shape, and PAT are apparent.

Supplementary Figure 5 – PAT from PPG

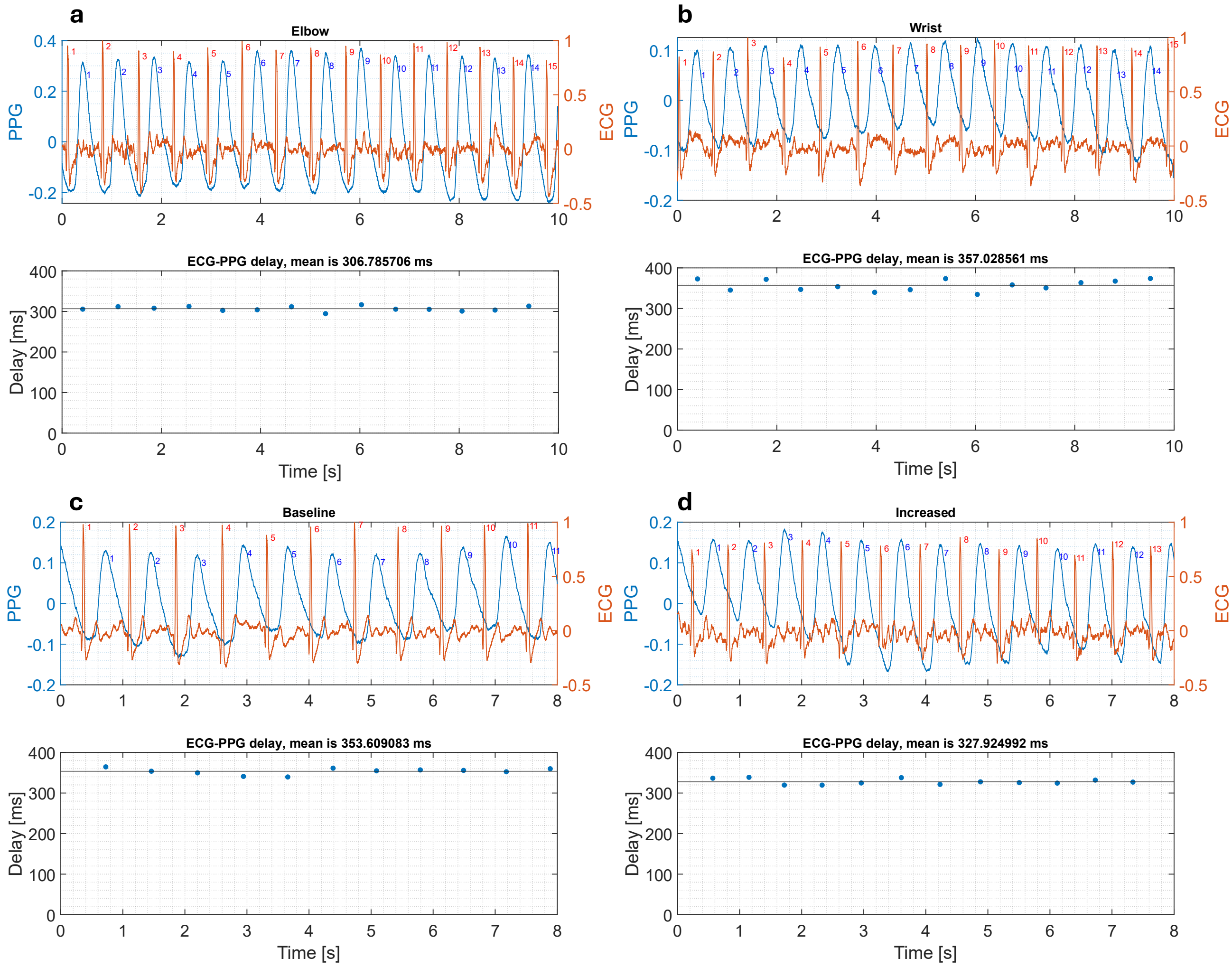

Supplementary Figure 5 - **PAT from PPG**. The PAT (pulse arrival time) measured at the **a** elbow and **b** wrist of a 29-year-old male, showing longer delays at the wrist due to its more distal location from the heart. **c-d** The PAT at the wrist measured at a baseline BP (blood pressure) value and at an increased BP value, showing that higher BP leads to reduced PAT.

Supplementary Figure 6 – The Telemed system

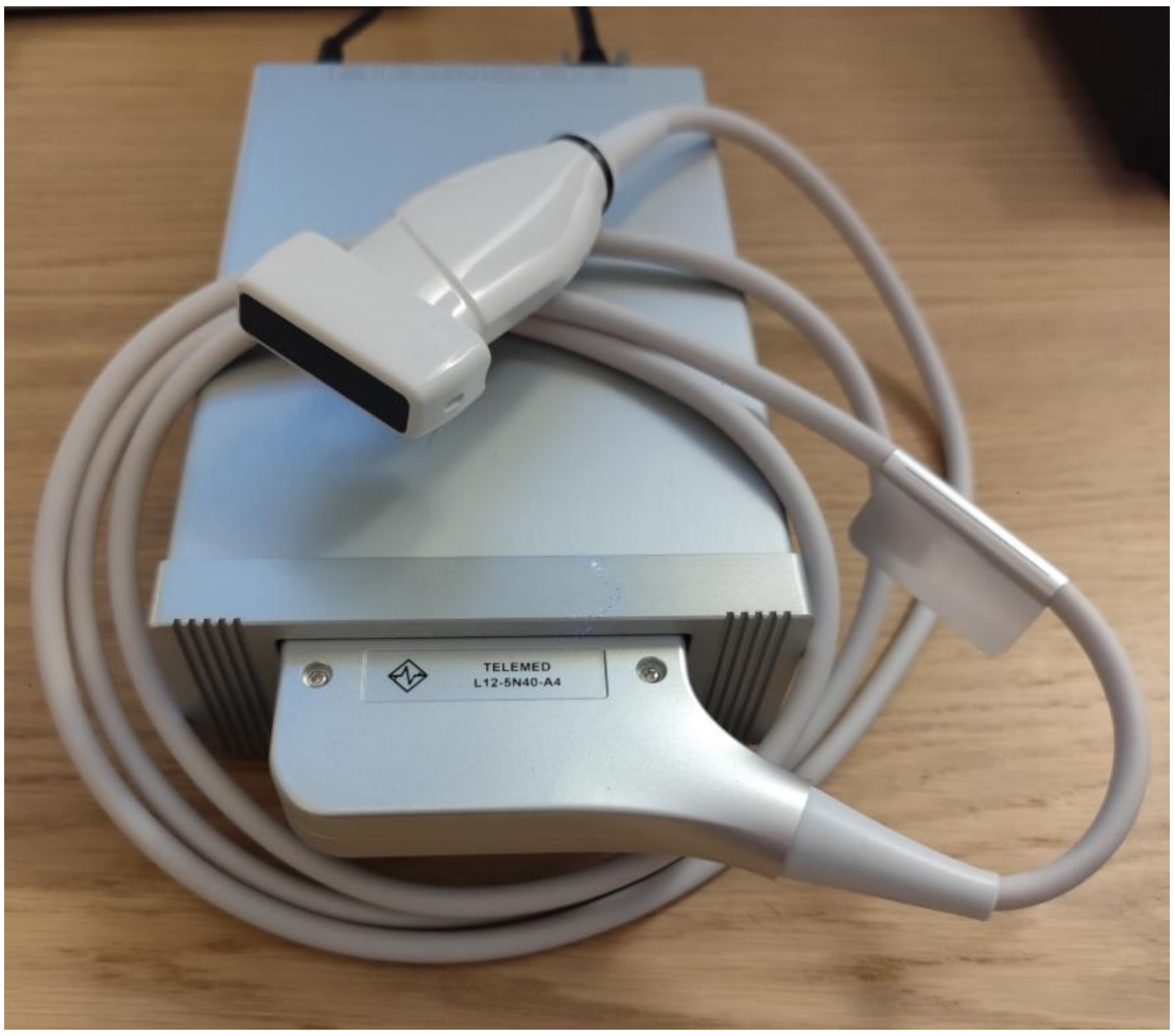

Supplementary Figure 6 – **The Telemed system**. A photograph of the ultrasound system (ArtUs USS-2H, Telemed, Lithuania) used in this work.

Supplementary Figure 7 – Ultrasound imaging of the wrist vessels

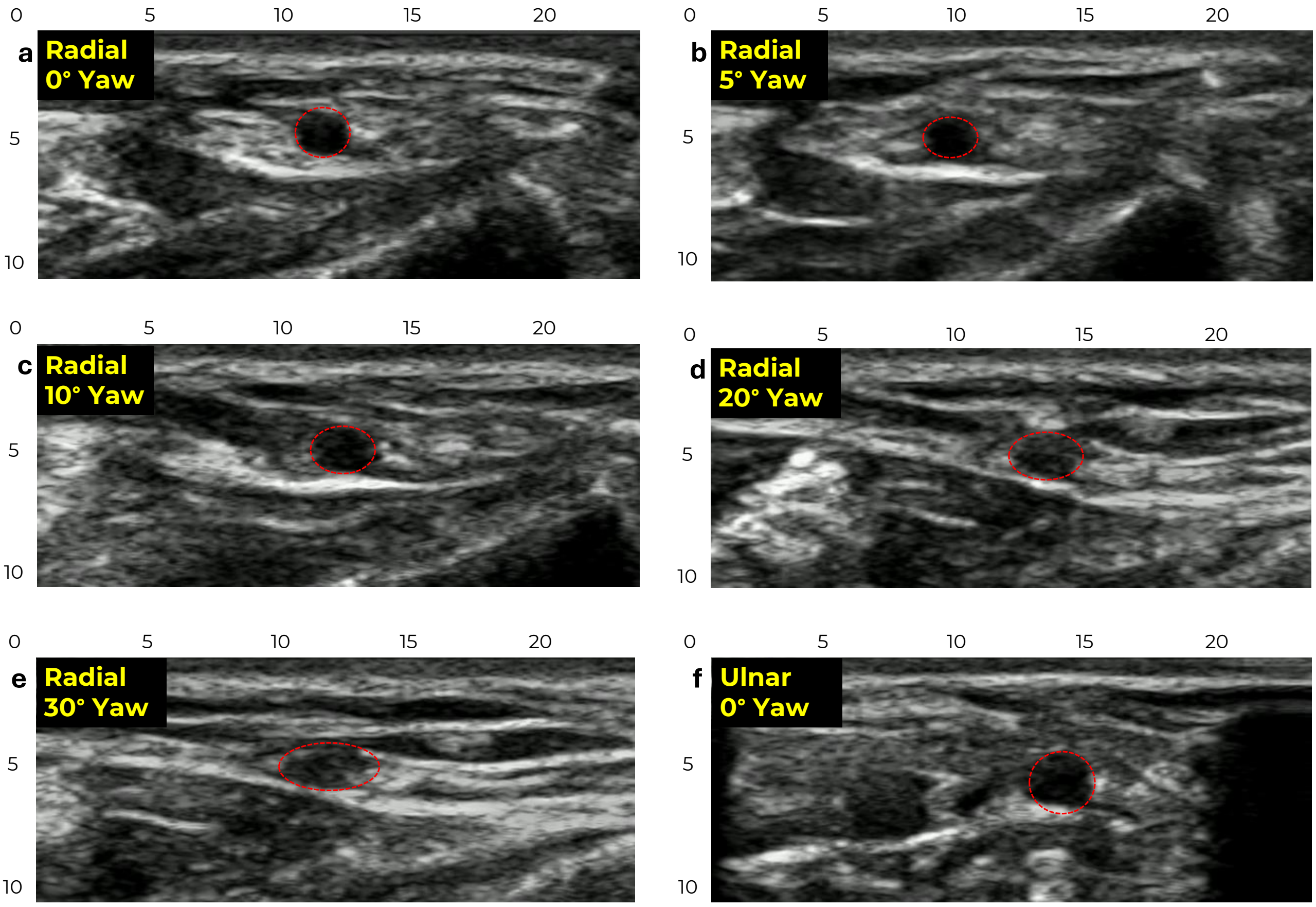

Supplementary Figure 7 – **Ultrasound imaging of the wrist vessels**. B-mode transversal images of a 29-year-old male with the ArtUs USS-2H (Telemed, Lithuania) operated at 12 MHz. **a** The radial artery with yaw of 0°. Below it is a muscle and the radial bone. **b** The radial artery with yaw of 5°, making it appear elliptical as we get an angled cross section of it. **c** The radial artery with yaw of 10°. **d** The radial artery with yaw of 20°. **e** The radial artery with yaw of 30°. **f** The ulnar artery is shown. It is often deeper than the radial for any given point along the forearm, making it an inferior test site. The axes ticks are in millimeters.

Supplementary Figure 8 – Pulse-echo diameter estimation with Tx/Rx using individual elements

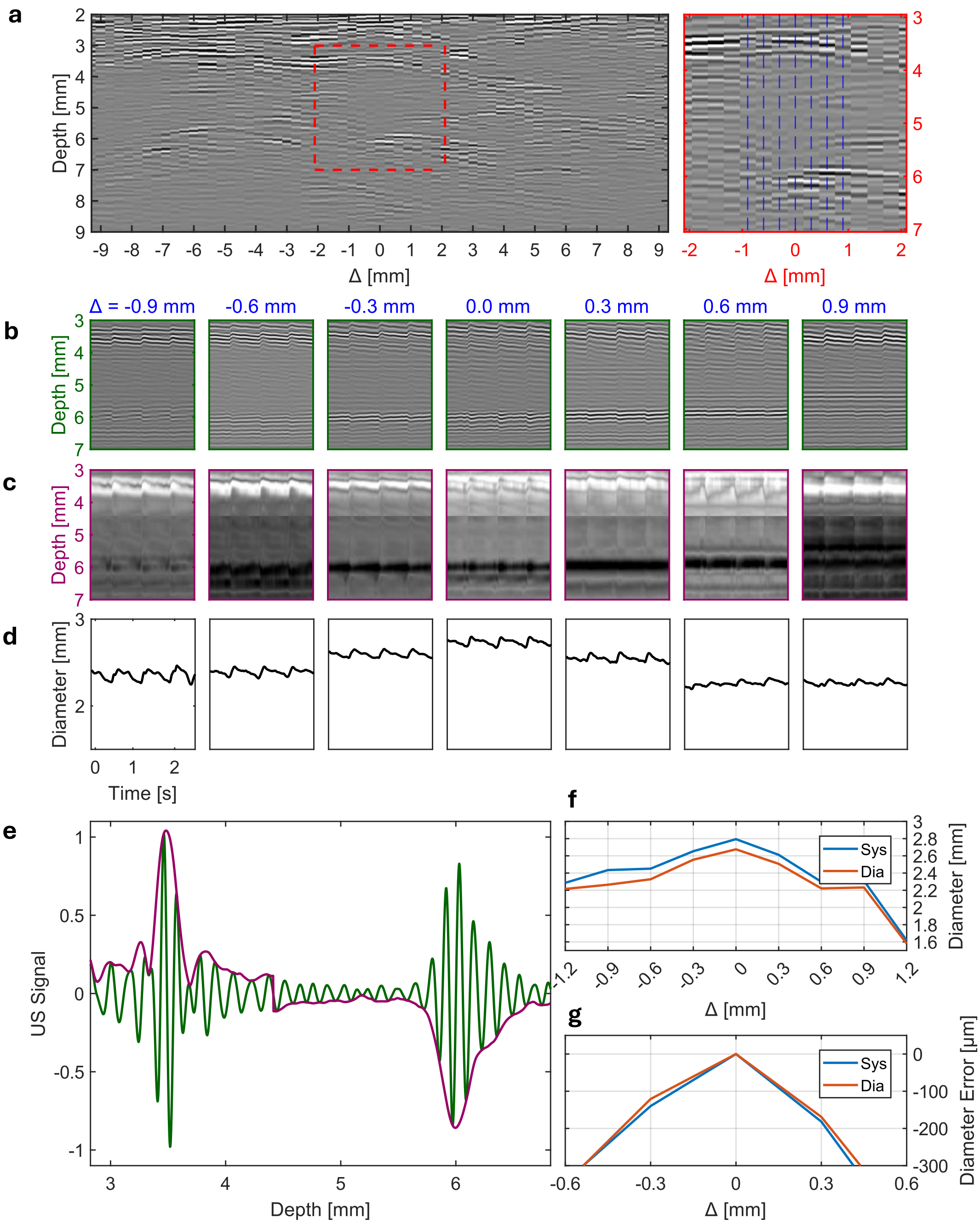

Supplementary Figure 8 – **Pulse-echo diameter estimation with Tx/Rx using individual elements.** Targeting the radial artery at the wrist region of a 29-year-old male using a pulse-echo US (ultrasound) transducer (ArtUs USS-2H, Telemed, Lithuania). **a** The raw signals of the individual elements, operating separately in pulse-echo mode, shown as a B-mode-like image with echoes from the radial artery visible in the ±1 mm range. **b** M-mode images for the elements in the ±1 mm range. **c** Post-processed M-mode images. **d** The diameters obtained from each element. **e** A typical raw US signal from the artery (green) and post-processed signal, obtained by Hilbert-based envelope detection and polarity inversion for the back of the artery wall (maroon). **f** The average estimated diameter as a function of sensor offset. **g** Diameter estimation error as a function of sensor offset.

Supplementary Figure 9 – Time of flight principle in optoacoustics

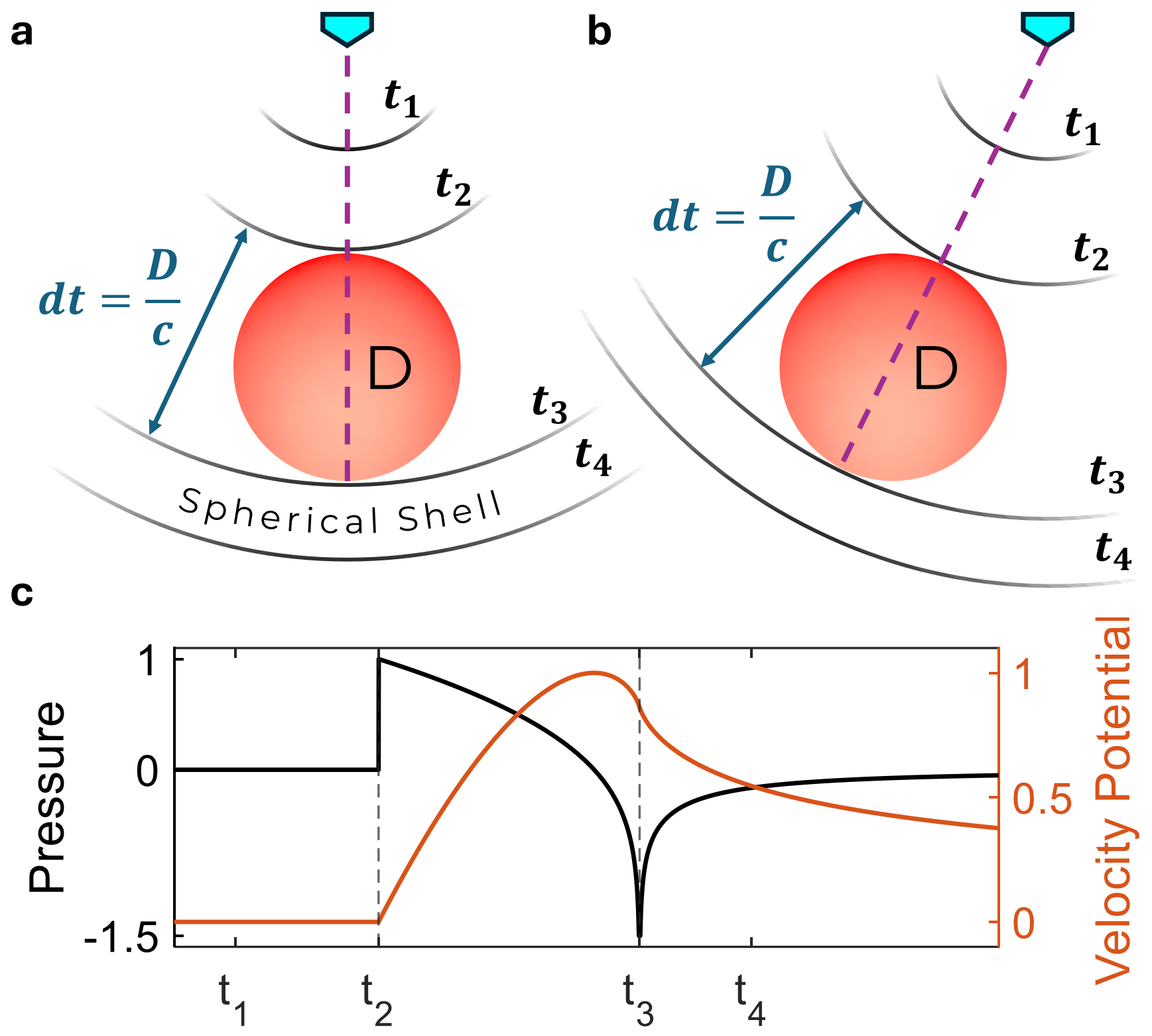

Supplementary Figure 9 - **Time of flight principle in optoacoustics**. **a-b** For each time instant t, the velocity potential is calculated by integrating the over optoacoustic sources within a spherical shell with a radius of ct. Accordingly, the velocity potential remains zero until the spherical shell first intersects with the cylindrical source at t_2. The velocity potential reaches an inflection point at t_3, after which it slowly decays to zero. **c** The Optoacoustic signal is represented by the pressure, which is equal to the temporal derivative of the velocity potential, and accordingly includes two discontinuities at t_2 and t_3. Specifically, at t_2 the signal experiences a step-like rise, whereas at t_3 the signal's derivative is discontinuous. These discontinuities are due to the geometrical overlap between the spherical shells and the boundary of the optoacoustic source, and therefore they also appear when the cylindrical source is not uniform, due to the decay in fluence.

Supplementary Figure 10 – The optoacoustic signal of a cylindrical source

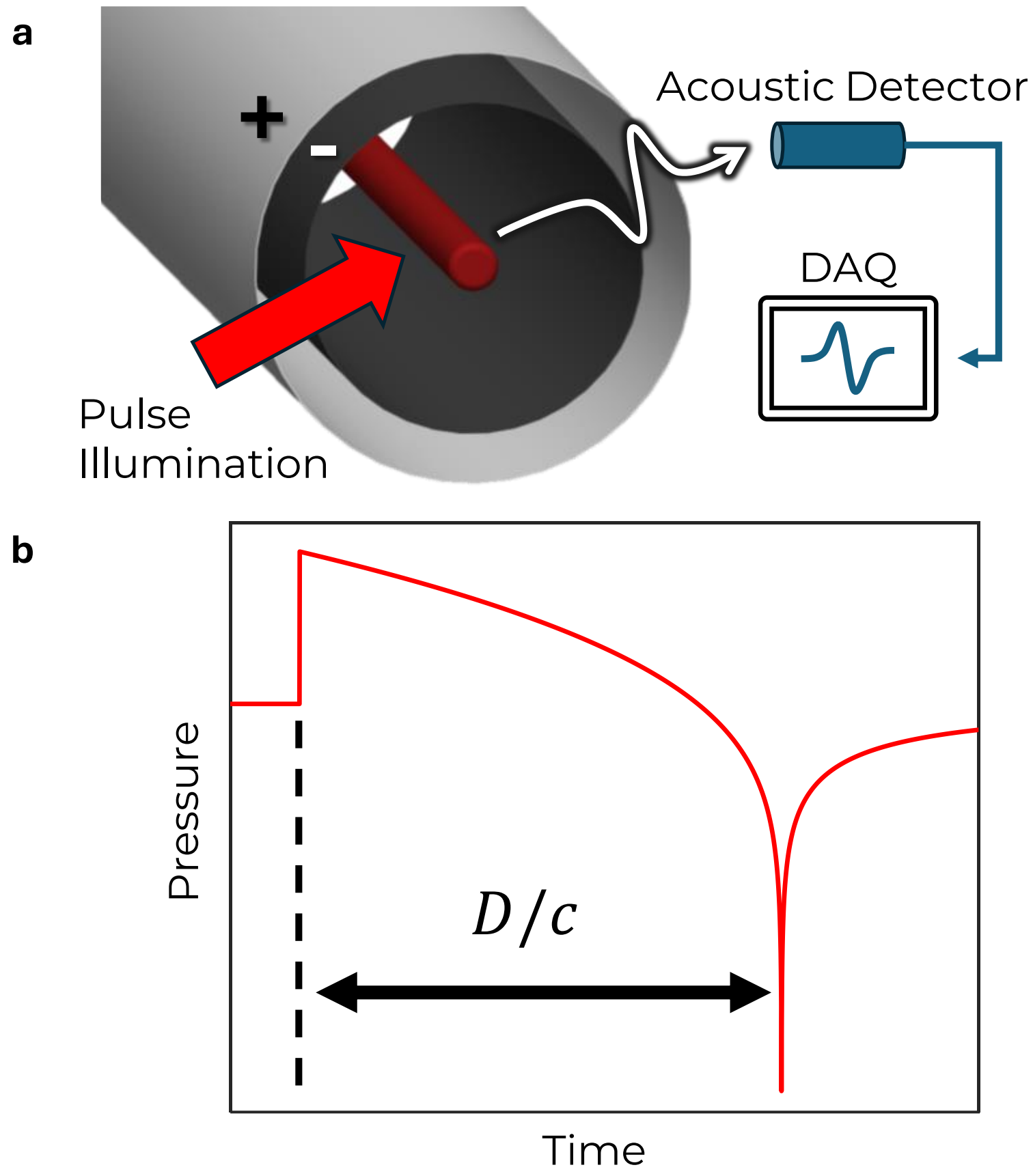

Supplementary Figure 10 - **The optoacoustic signal of a cylindrical source**. **a** The optoacoustic effect induced by a uniform cylindrical source, in which the wavefronts are also cylindrical. **b** Evaluation of the S11 expression for the optoacoustic signal from an infinite and uniform cylindrical source, measured up by a point sensor with high bandwidth in a lossless medium.

Supplementary Figure 11 – Diffuse-optics Monte-Carlo simulation

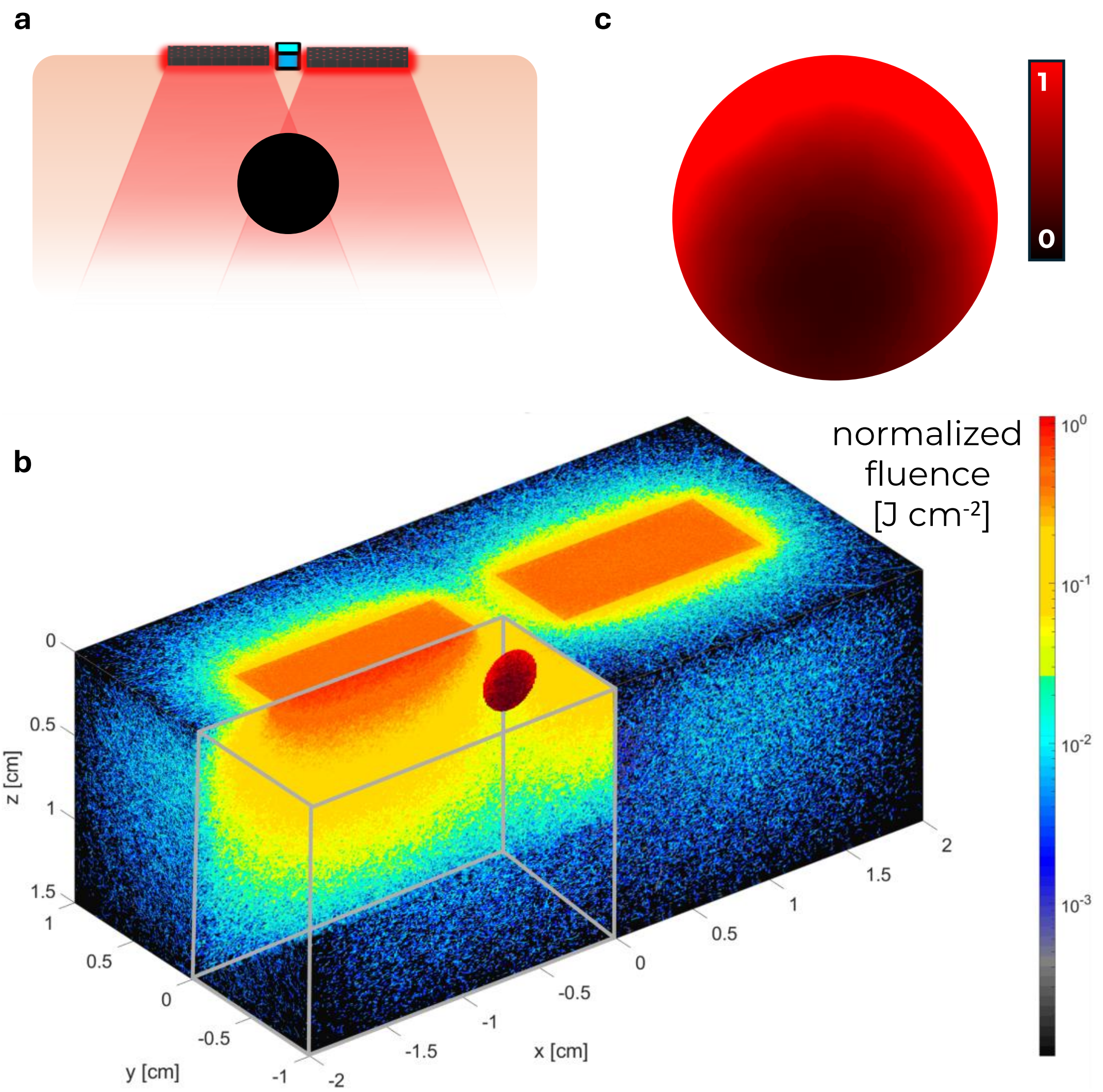

Supplementary Figure 11 – **Diffuse-optics Monte-Carlo simulation**. **a** An illustration of the illumination configuration in the optoacoustic sensor. **b** A 3D photon fluence map, simulated with MCmatlab [45]. **c** Magnified image of the resulting absorption distribution (heating function) in an artery with a diameter of 3 mm at a depth of 4 mm.

Supplementary Figure 12 – Fluence distribution

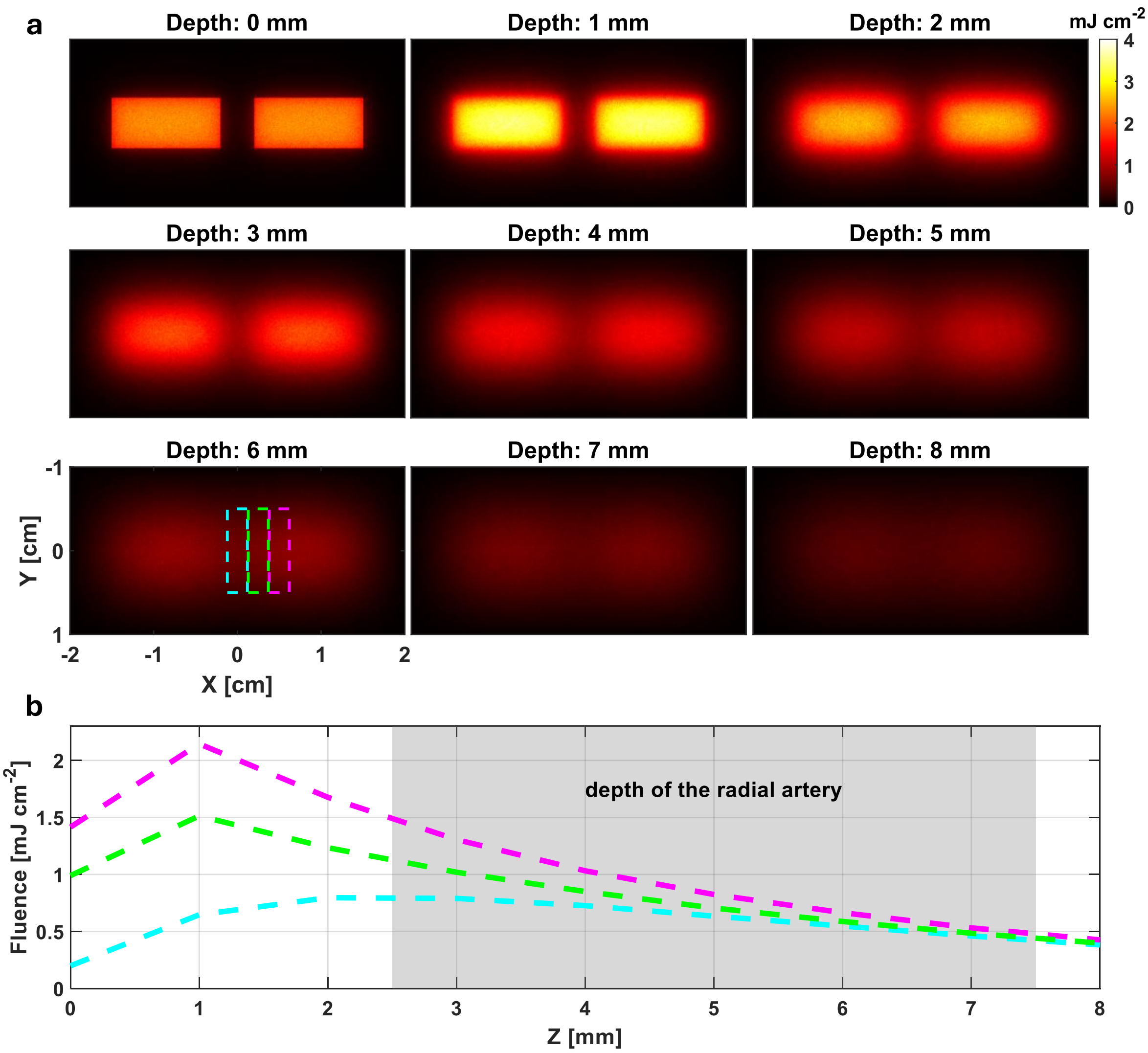

Supplementary Figure 12 – **Fluence distribution**. **a** A progression of fluence maps for different depths, as resulted from the MCmatlab engine for the illumination condition of the optoacoustic probe, simulated without blood vessels. **b** Fluence as a function of depth, averaged over three possible artery offset locations. For the relevant depths where the radial artery may be found, an acceptable variation in the fluence level is found.

Supplementary Figure 13 – NEP, BW, and PSF evaluation systems

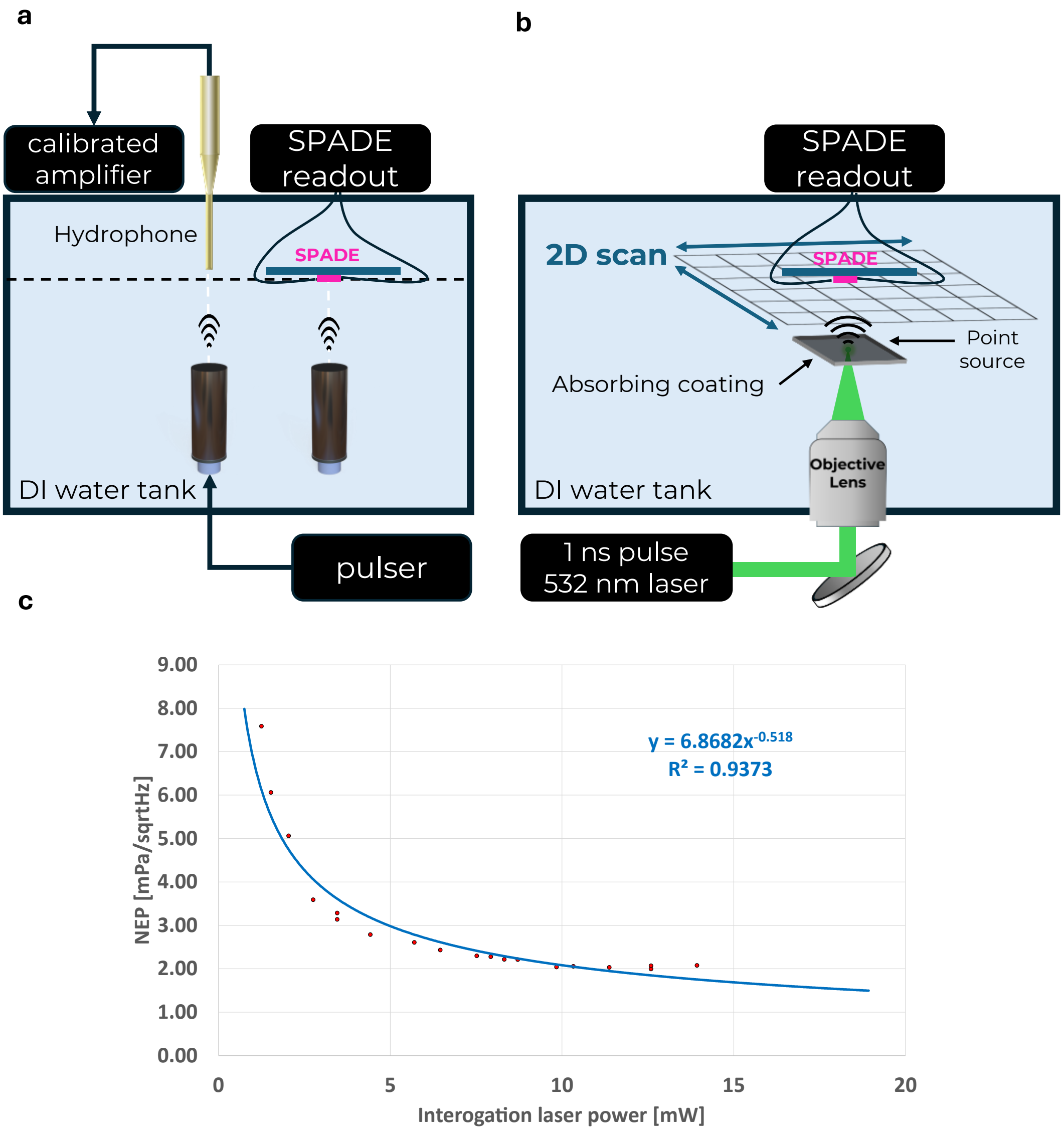

Supplementary Figure 13 – **NEP, BW, and PSF evaluation systems**. **a** An illustration of the NEP (noise equivalent pressure) measurement system. The system used a calibrated hydrophone (NH1000, Precision Acoustics, UK) and a 0.5", 5 MHz central frequency ultrasonic transducer (Evident Scientific, Japan) which transmitted low intensity (1.1 kPa) pulses. **b** An illustration of the system used to measure the PSF (point spread function) & BW (bandwidth) of the SPADE (silicon photonics acoustic detector). The system uses a 532 nm, 1 ns FWHM pulse laser (WAVEGUARD-D, Optogama, Lithuania) focused by an objective to a 5 µm spot on a glass slide coated with a thin layer of absorbing coating. The light induced the optoacoustic effect and resulted in a wideband point-like acoustic source. The SPADE was precisely scanned in plane using servo-operated mechanical stages (Physik Instrumente, Germany) and the acoustic signal was measured for each position. The BW measurement used the position in which the source was positioned in front of the center of SPADE. **c** The dependency on the NEP on the power of the interrogation laser, showing shot-noise behavior in which the NEP is proportional to the square root of the laser power. Processed using Microsoft Excel.

Supplementary Figure 14 – Phantom experiment with SPADE

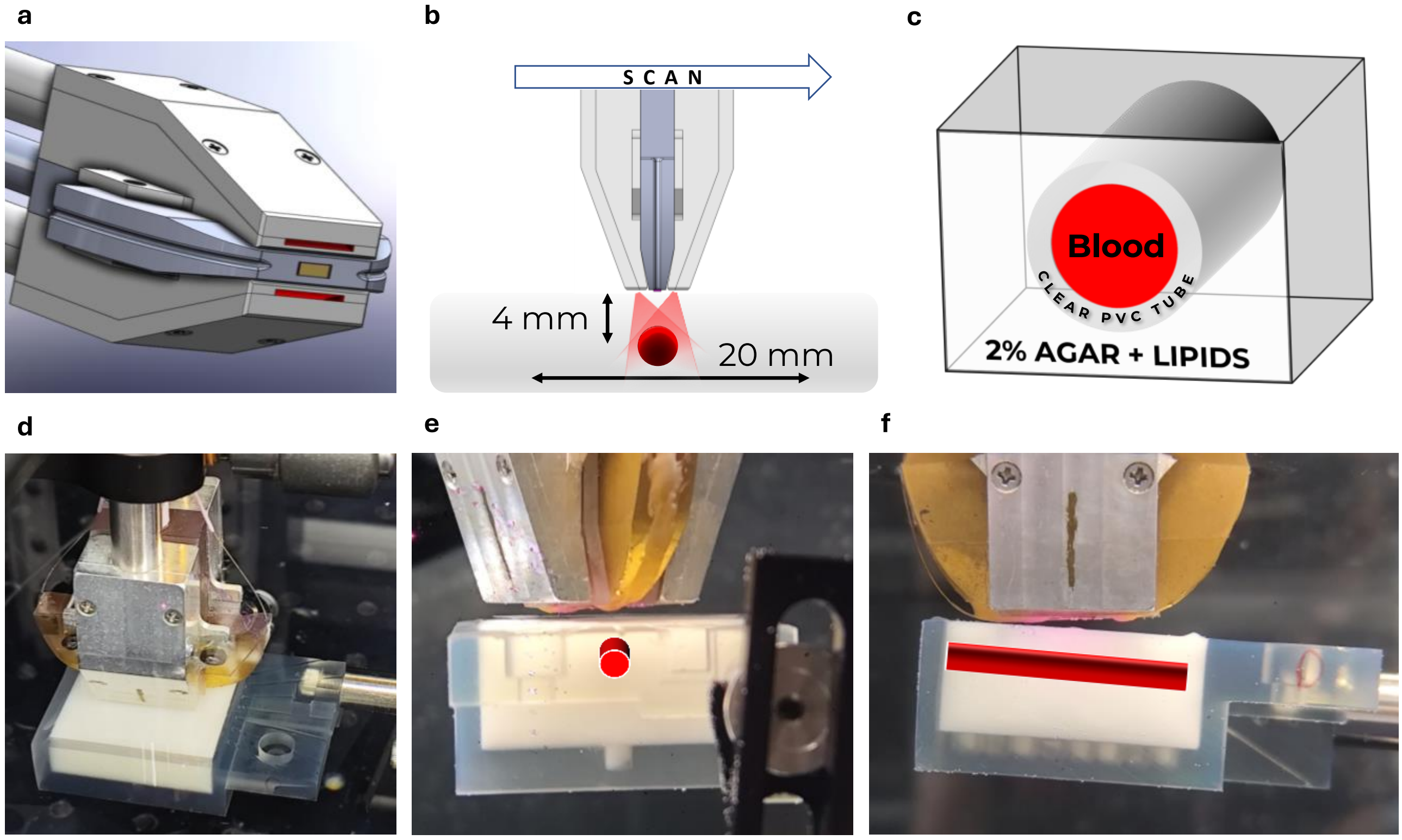

Supplementary Figure 14 - **Phantom experiment with SPADE**. **a** CAD illustration of a phantom-compatible version of the SPADE-based optoacoustic probe, where the SPADE (silicon photonics acoustic detector) probe is mounted on a frame that can be easily moved by a mechanical stage and the illumination bundle is submergible. The CAD file of the fiber bundle was originally made by CeramOptec (Germany), included with permission. **b** The phantoms were scanned over a range of ±10 mm from the center of the cylindrical inclusion. **c** Phantom cross section and composition. **d** A photograph of the experiment, with **e** yaw alteration, and **f** pitch alteration.

Supplementary Figure 15 – Phantoms imaging and 1D signals from SPADE and from piezoelectric sensors

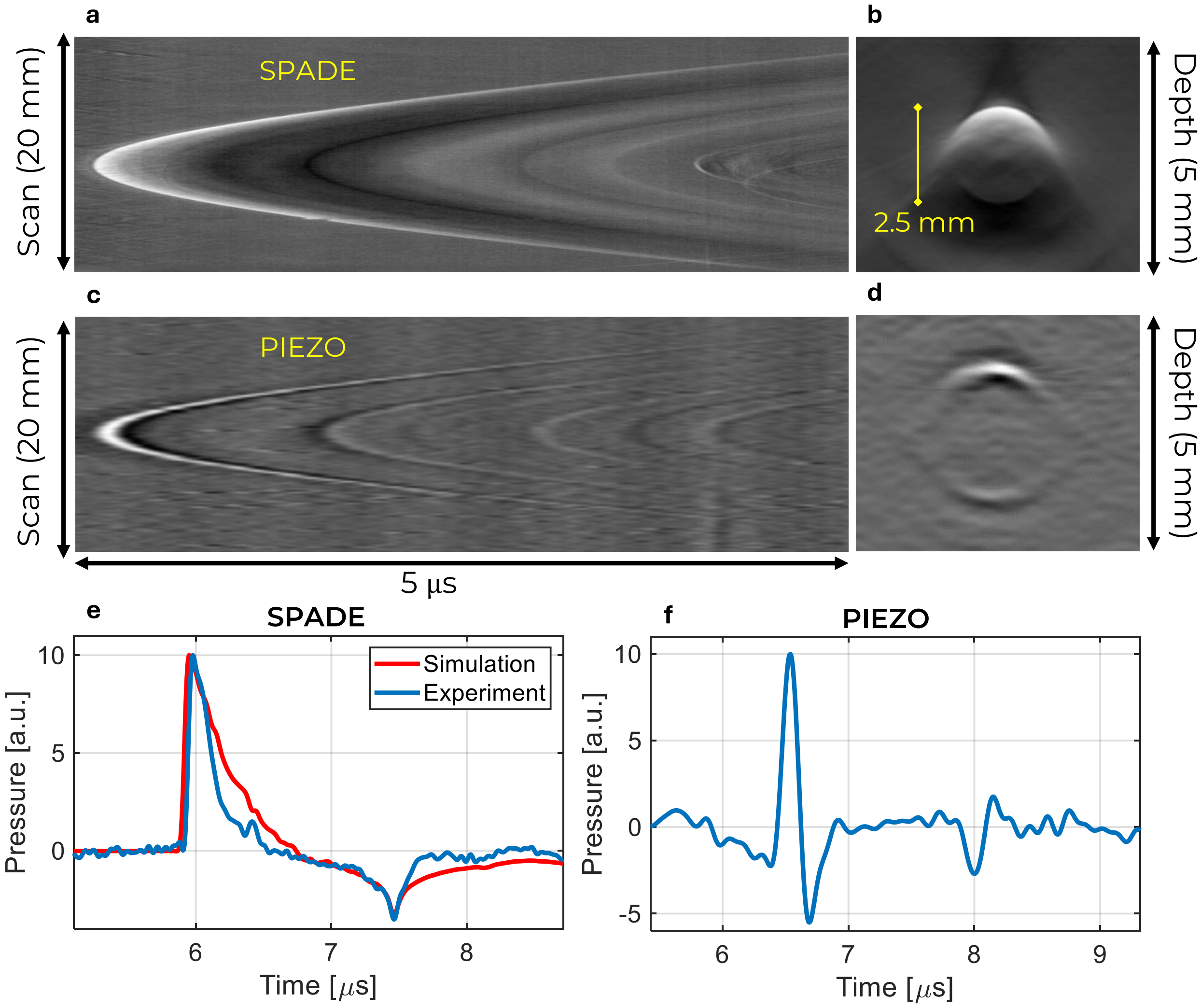

Supplementary Figure 15 - **Phantoms imaging and 1D signals from SPADE and from piezoelectric sensors**. **a** Sinogram from a phantom scanned by a SPADE (silicon photonics acoustic detector) based probe. **b** Back-projection reconstruction of the heating function measured by SPADE. **c** Sinogram from the same phantom scanned by the PE-OA (piezoelectric optoacoustic) imager. **d** Back-projection reconstruction of the heating function measured by the PE-OA imager. The effect of the lower bandwidth and acceptance angle is readily seen in the reconstructed image. **e** The 1D optoacoustic signal from a simulation and from a matching phantom as measured by the SPADE. **f** The 1D optoacoustic signal for the same phantom, measured with the PE-OA probe. The narrower bandwidth of the piezoelectric transducer is particularly apparent in the discontinuity of the leading edge.

Supplementary Figure 16 – Pitch angles of the radial artery

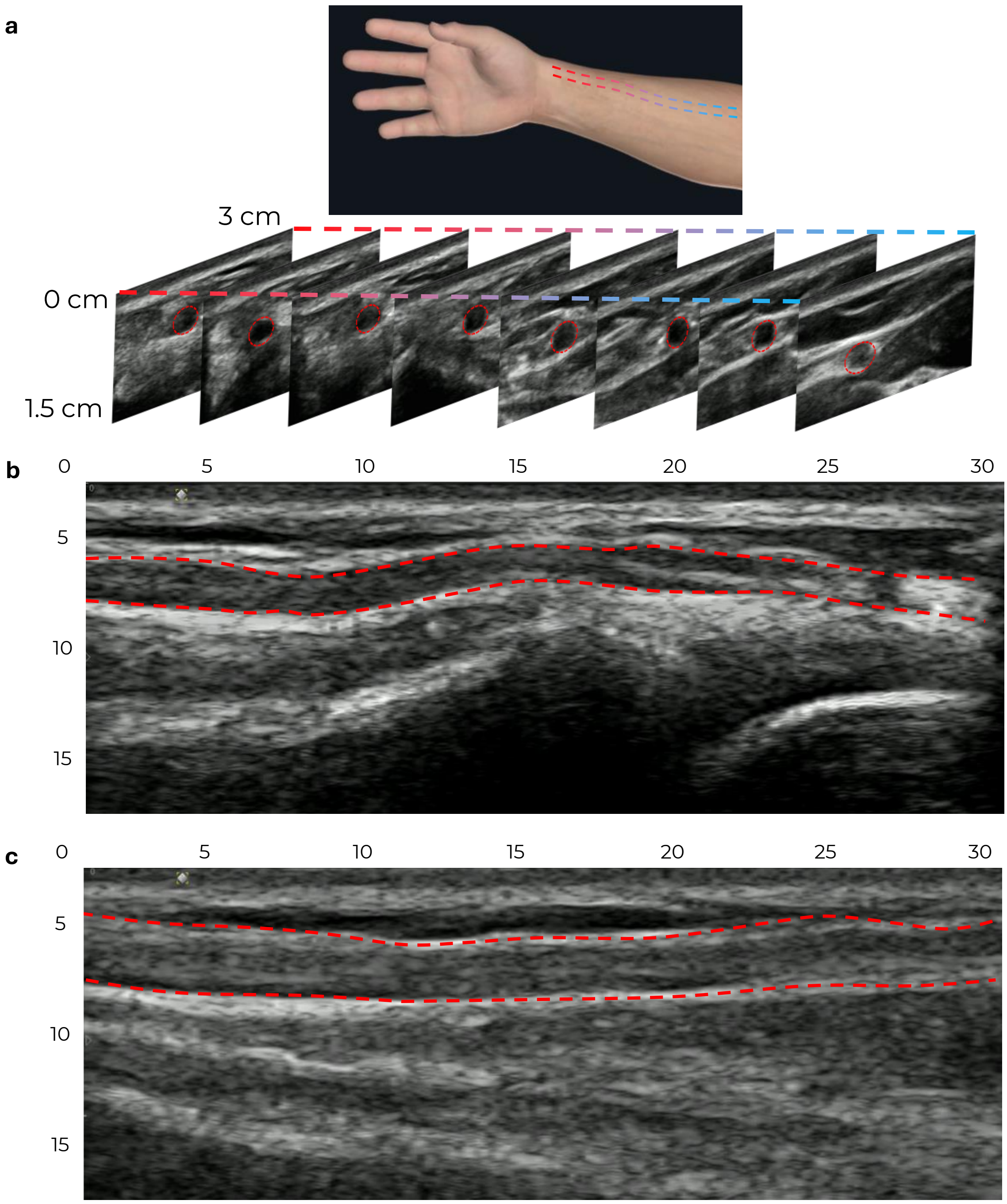

Supplementary Figure 16 – **Pitch angles of the radial artery**. B-mode ultrasound imaging (ArtUs USS-2H, Telemed, Lithuania - operated at 12 MHz) of the radial artery of a 29-year-old male. The artery changes in diameter, pitch and depth along its path. **a** A sequence of transverse view slices of the radial artery along the forearm. **b**-**c** Two longitudinal views of the radial artery from different positions along the forearm. The axes ticks are in millimeters.

Supplementary Figure 17 – Phantoms diameter estimation with SPADE

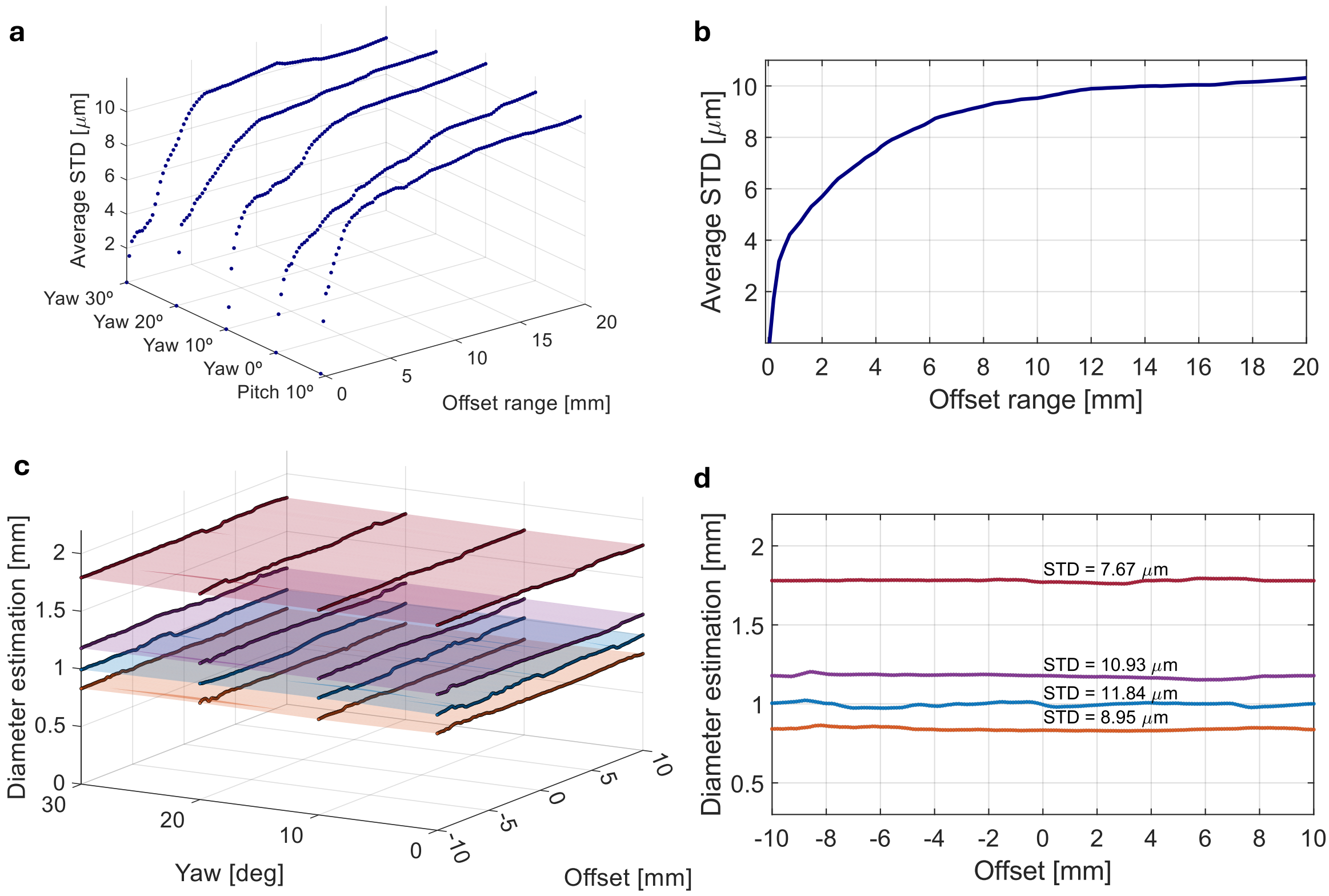

Supplementary Figure 17 - **Phantoms diameter estimation with SPADE**. **a** Diameter estimation STD (standard deviation) as a function of the offset range over which it was measured, calculated for several orientations and averaged across all diameters of the cylindrical inclusions used in the phantoms. **b** Diameter estimation STD as a function of offset range, averaged across all diameters and orientations. **c** Diameter estimation as a function of offset and orientation for selected diameters. **d** Diameter estimation as a function of offset for 0° yaw and pitch, with corresponding STD over the entire range.

Supplementary Figure 18 – Blood pressure model error propagation

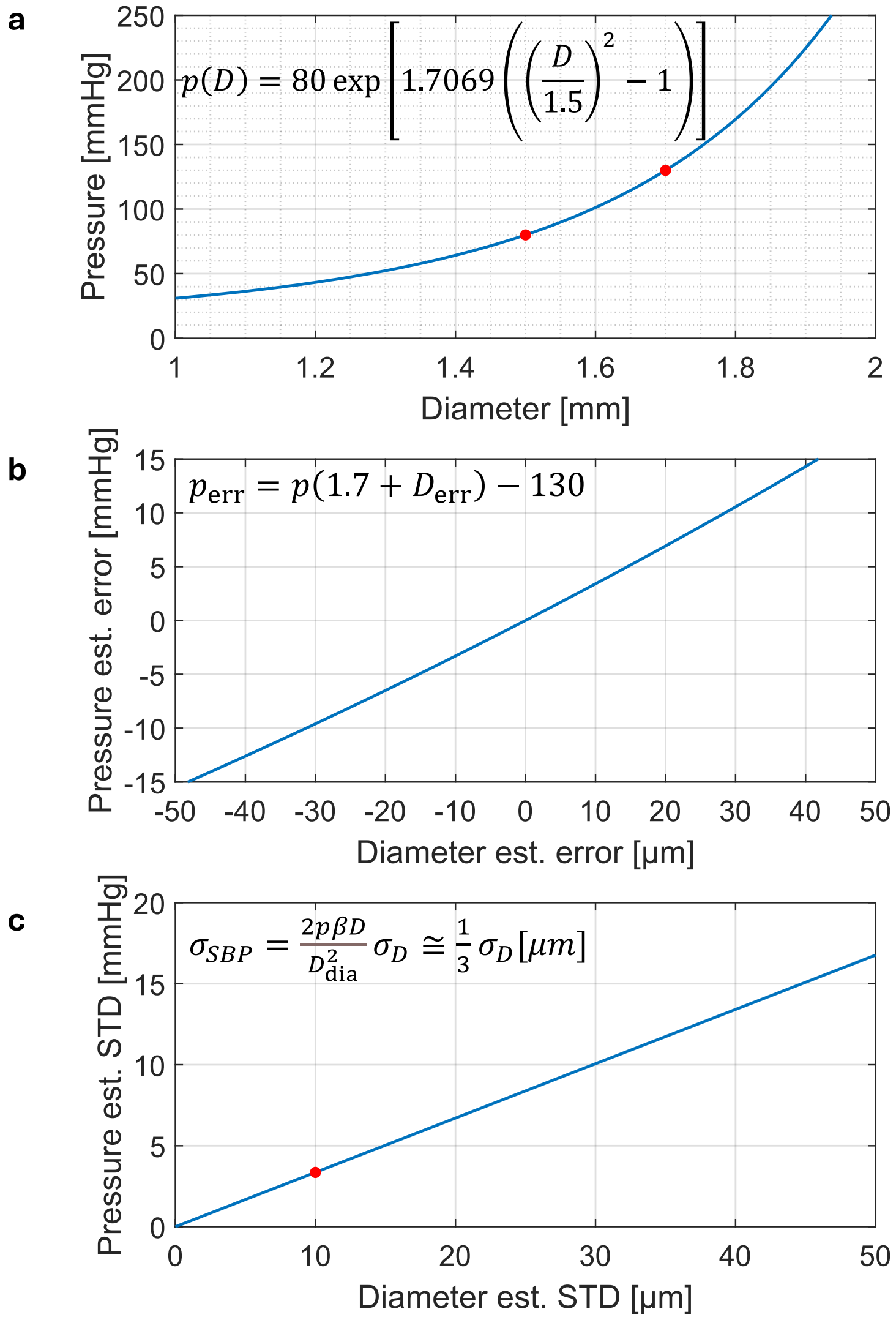

Supplementary Figure 18 – **Blood pressure model error propagation**. **a** The pressure estimation equation after calibration. Setting the systolic calibration diameter gives the exact value. **b** The SBP (systolic blood pressure) estimation error as a function of diameter estimation error. **c** The STD (standard deviation) of the estimated SBP as a function of the STD of the diameter reading.

Supplementary Figure 19 – The piezoelectric optoacoustic imager (PE-OA)

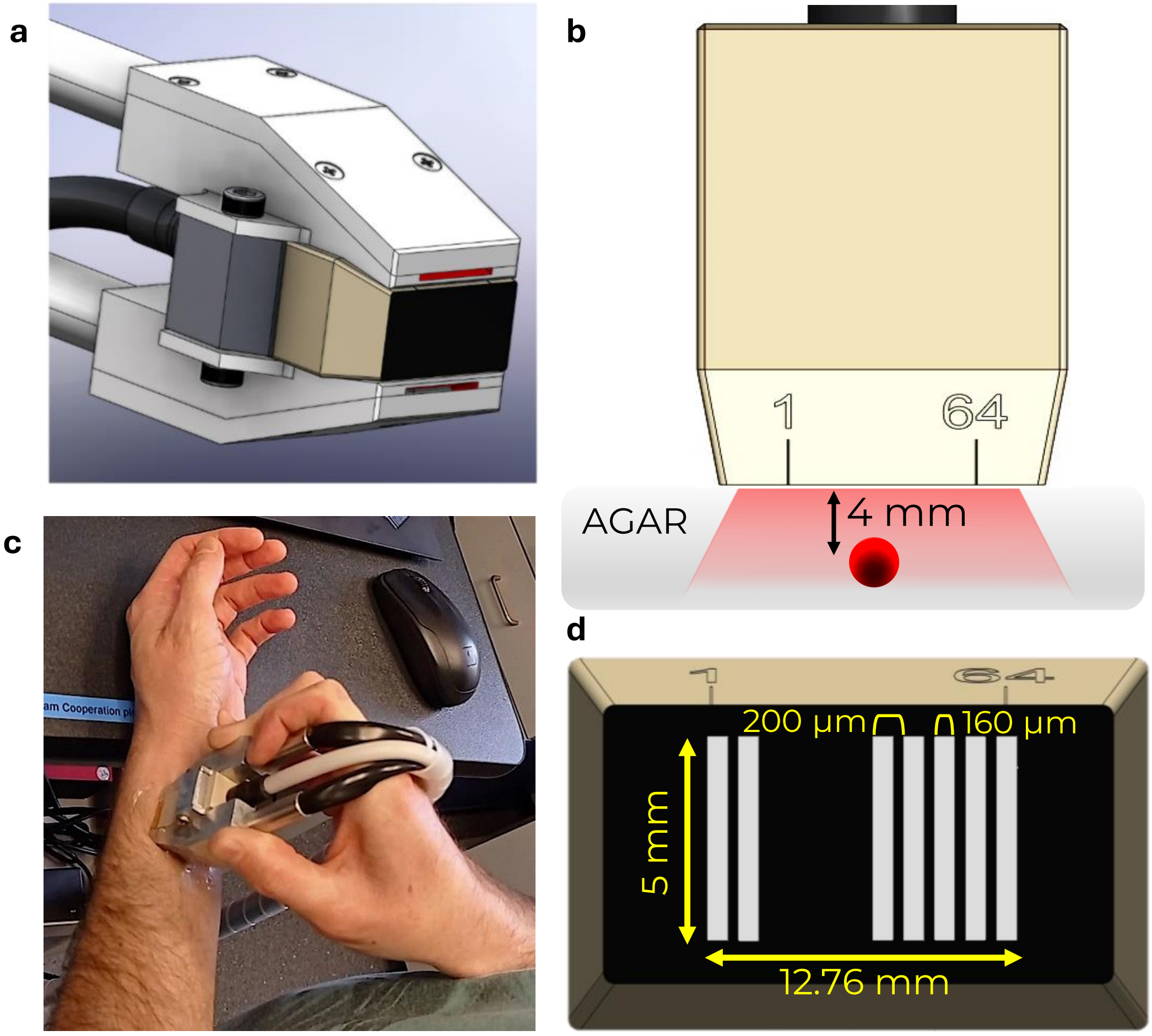

Supplementary Figure 19 - **The piezoelectric optoacoustic imager (PE-OA)**. **a** CAD illustration of the PE-OA probe. The CAD file of the transducer was originally made by IMASONIC (France), included with permission. **b** The 64-element PE transducer used to detect the optoacoustic signal shown in scale to the depth and diameter of the radial artery close to the wrist. **c** A photograph showing how the PE-OA probe was used *in vivo*. **d** Schematics of the 64 sensing elements: the pitch of the array is 200 µm, the transverse aperture is 5 mm, and the span of the array is 12.76 mm.

Supplementary Figure 20 – Piezoelectric optoacoustic *in vivo* imaging

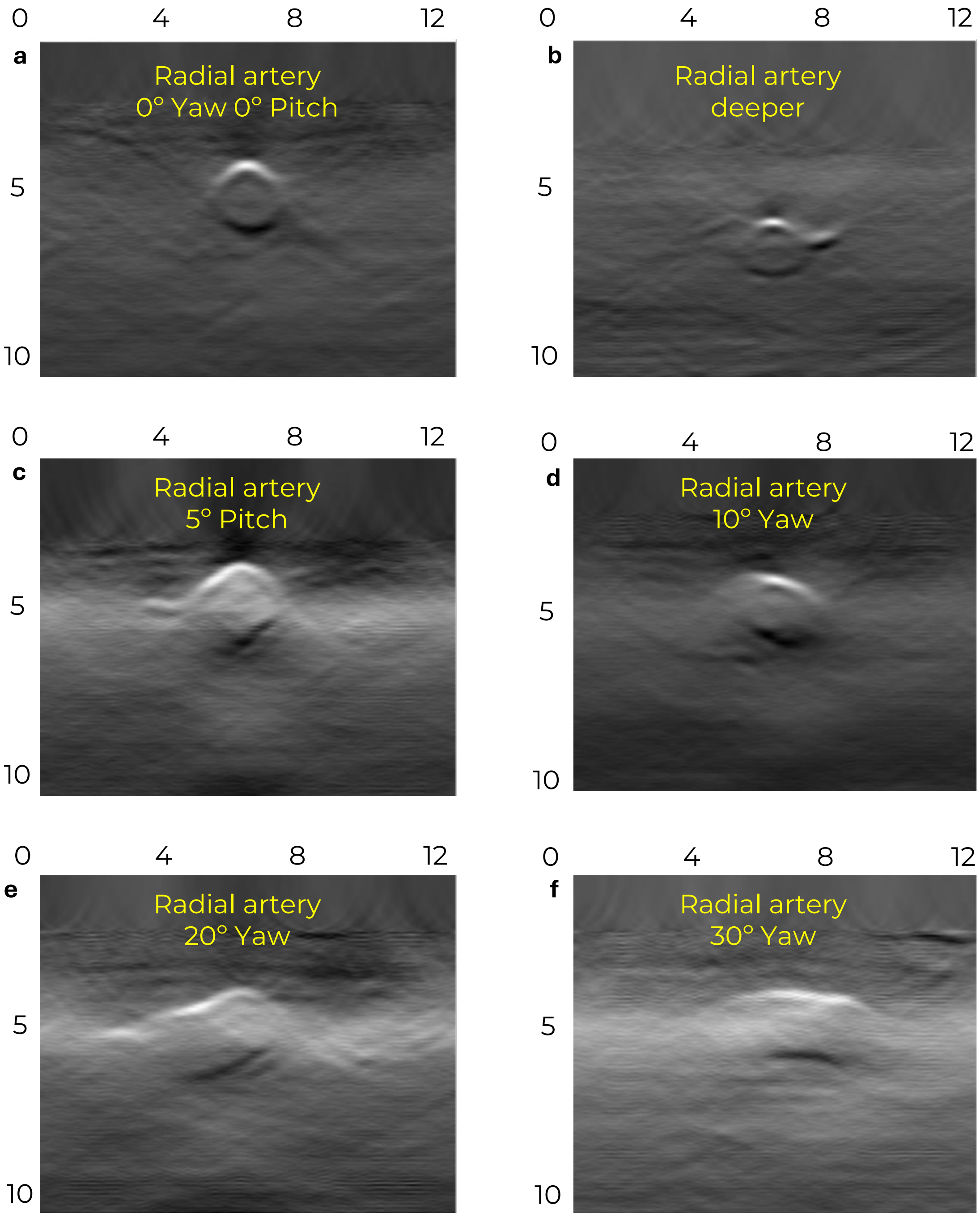

Supplementary Figure 20 – **Piezoelectric optoacoustic *in vivo* imaging**. **a-f** Piezoelectric optoacoustic *in vivo* (29-year-old male) imaging of the radial artery at different orientations. The axes ticks are in millimeters.

Supplementary Figure 21 - Piezoelectric optoacoustics yaw sensitivity

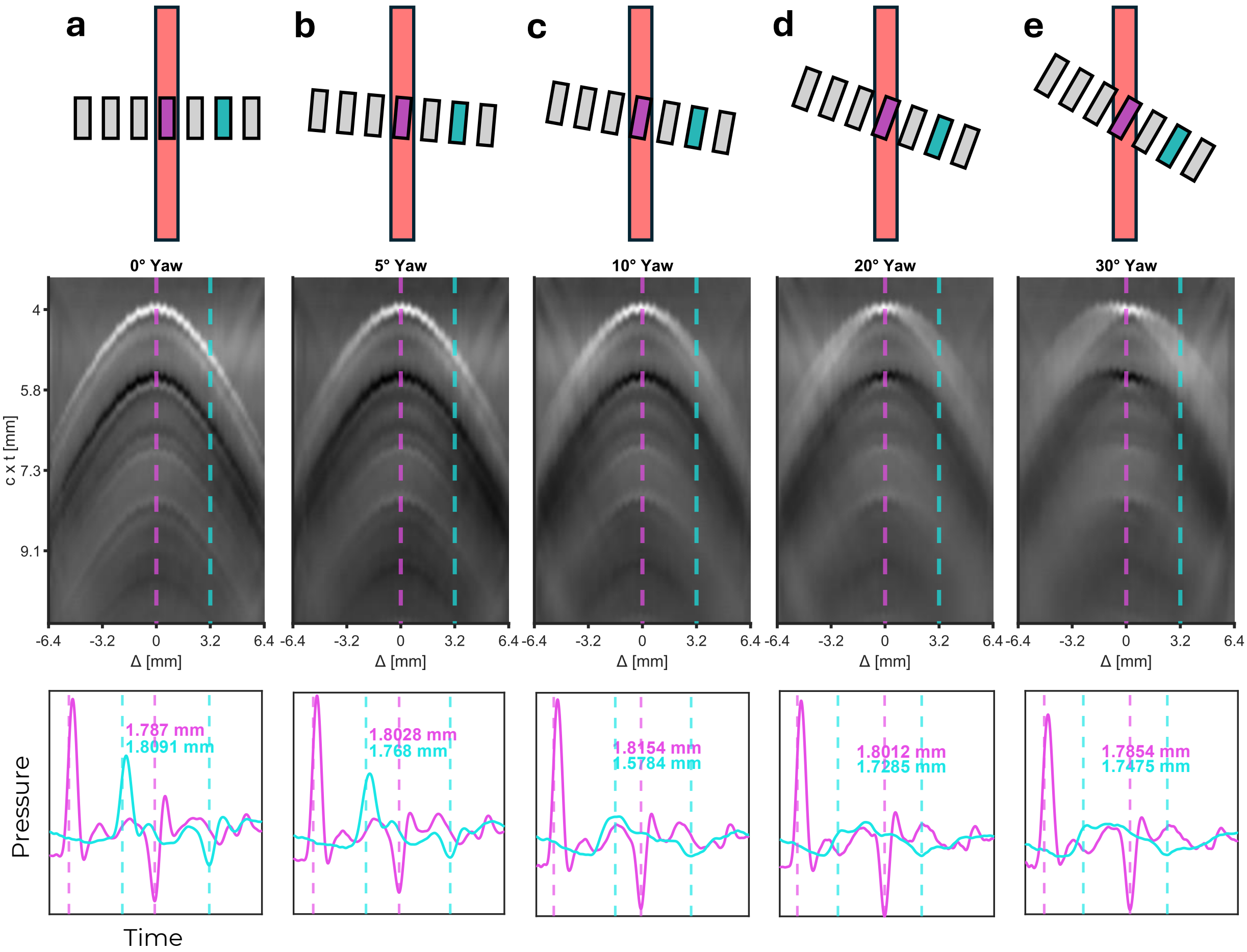

Supplementary Figure 21 - **Piezoelectric optoacoustics yaw sensitivity**. **a**-**e** The results of a phantom measured by the piezoelectric optoacoustic system for increasing yaw angles. As yaw increases beyond 5°, sensors with offset experience significant distortion. Diameter estimation for these sensors is inaccurate and unstable.

Supplementary Figure 22 – Phantoms diameter estimation with piezoelectric optoacoustics

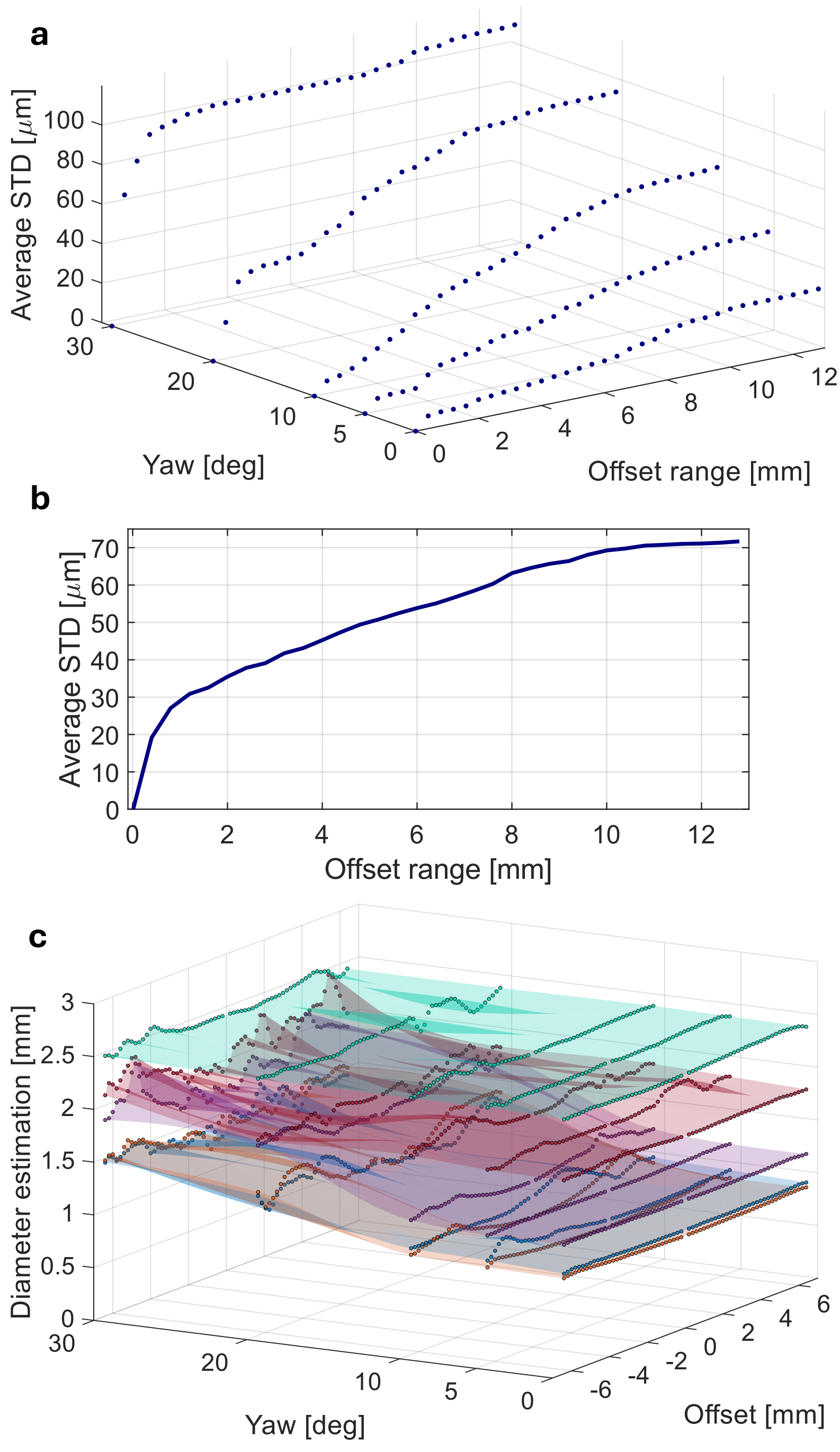

Supplementary Figure 22 - **Phantoms diameter estimation with piezoelectric optoacoustics**. **a** Diameter-estimation STD (standard deviation) as a function of the offset range over which it was measured, calculated for several orientations and averaged across all diameters of the cylindrical inclusions used in the phantoms. **b** Diameter estimation STD as a function of offset range, averaged across all diameters and orientations. **c** Diameter estimation as a function of offset and yaw angle for 5 selected diameters.

Supplementary Figure 23 – |Max/Min| ratio of the optoacoustic signal

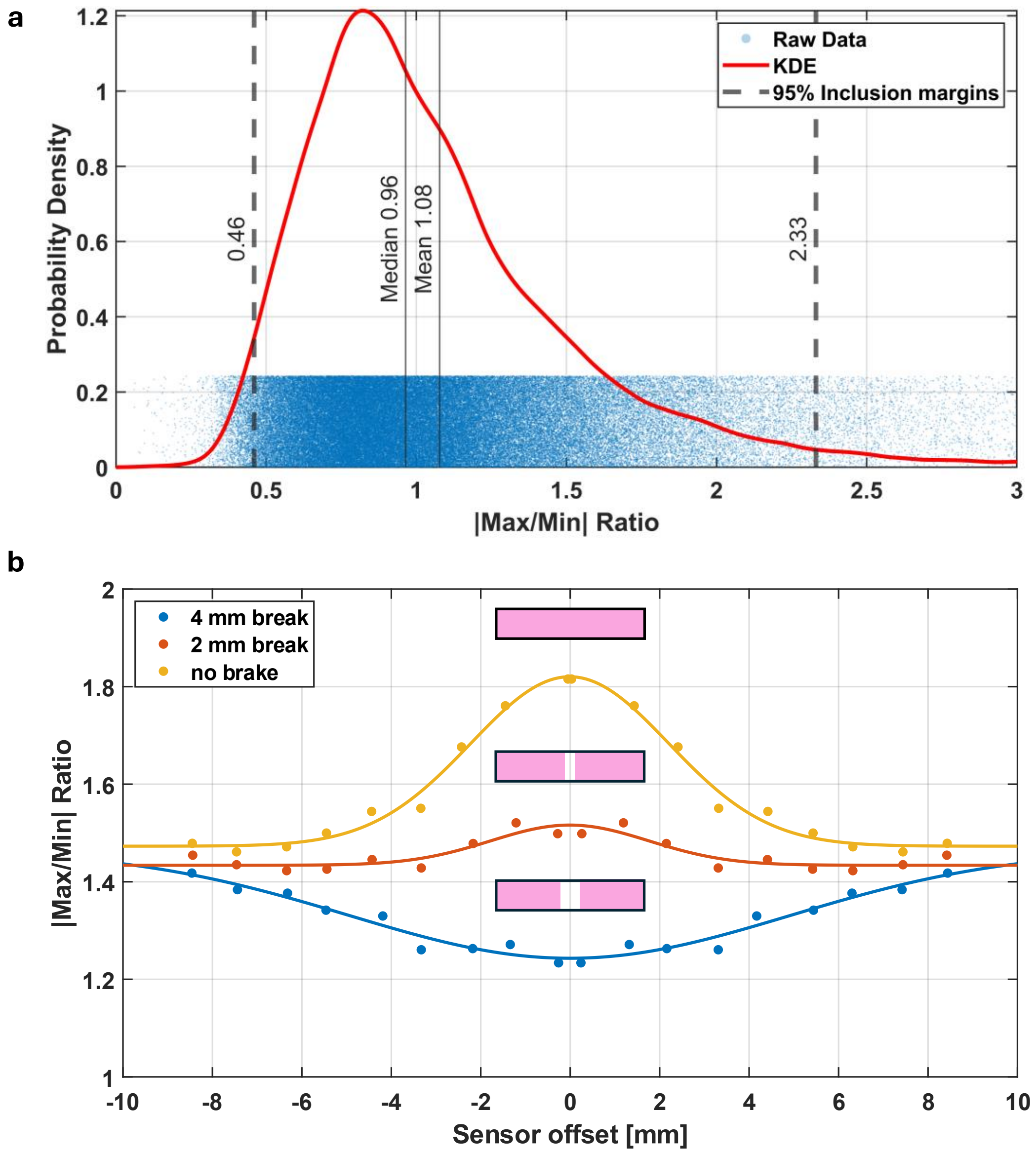

Supplementary Figure 23 – **|Max/Min| ratio of the optoacoustic signal**. **a** A distribution of |max/min| ratios from the *in vivo* study with the SPADE optoacoustic probe. Each count is a single trigger event, and a total of n = 120,000 events are shown from 90 randomly selected measurements (male and female aggregated). Counts below a ratio of 1 can be attributed to the presence of the radial bone, reflecting light towards the posterior end of the artery. **b** |Max/Min| ratio of the simulated optoacoustic signal for three types of illumination schemes, where our dark-field setup with the 4 mm break in the illumination pattern yields favorable results over a smaller break, or no break in the illumination at all. The scattered simulated points are overlaid with a gaussian fit.

Supplementary Figure 24 – *In vivo* diameter estimation at different offsets using a single element of a piezoelectric optoacoustic probe with ideal orientation

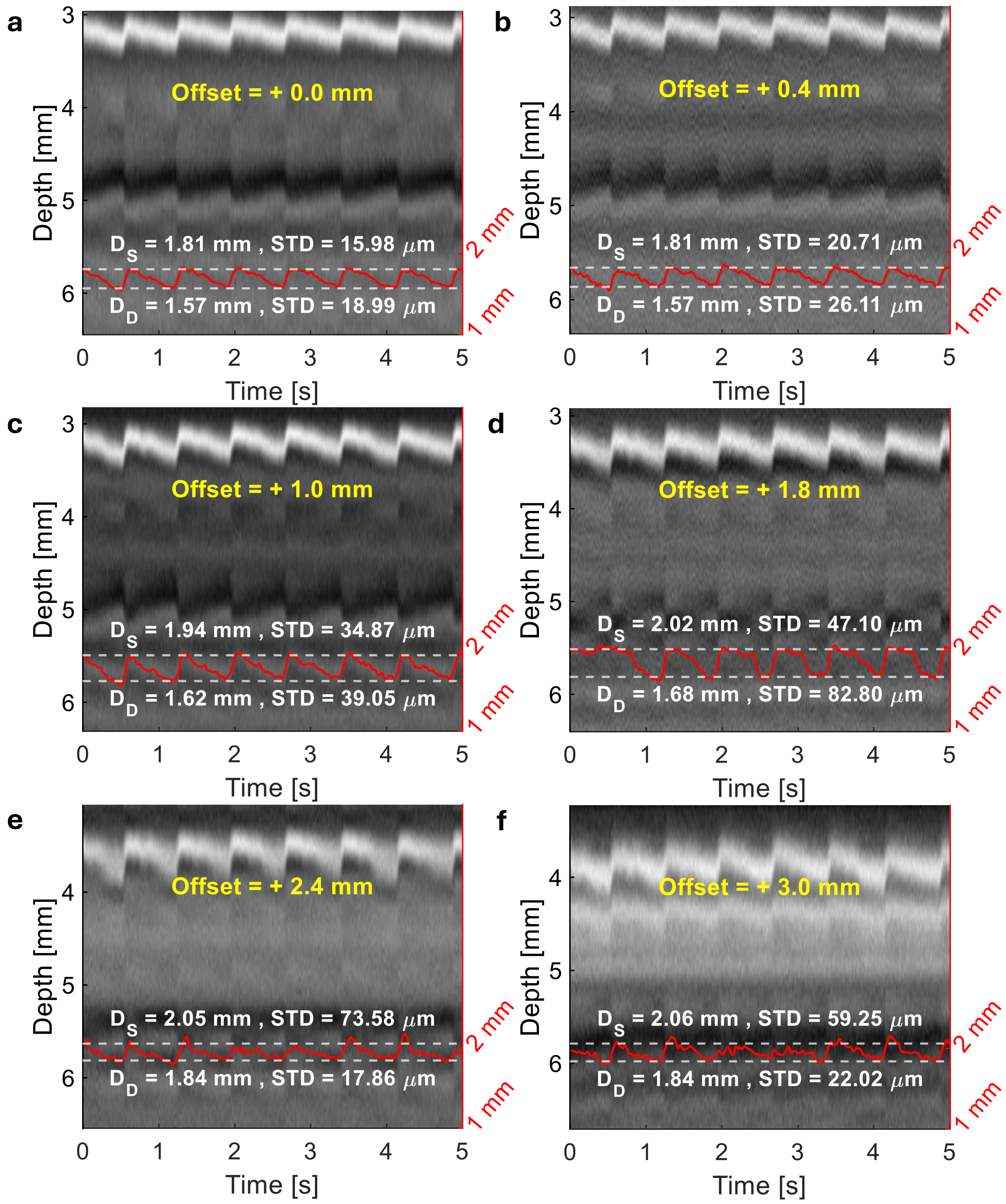

Supplementary Figure 24 – ***In vivo* diameter estimation at different offsets using a single element of a piezoelectric optoacoustic probe with ideal orientation**. The piezoelectric optoacoustic imager was used to estimate the diameter of the radial artery close to the wrist of a 29-year-old male using only one of its elements. Subfigures **a**-**f** show the M-mode images and extracted diameter waveforms for different element offsets from the center of the artery and corresponding diameter waveforms (red trace). As the offset increases, the SNR of the optoacoustic signal diminishes and its shape undergoes distortion, leading to reduced consistency in the diameter estimation and deformation of the diameter waveform. Additionally, the diameter values drift with the offset, resulting in diameter STD (standard deviation) of 115 µm (systolic) and 125 µm (diastolic), calculated over the ±3 mm offset range.

Supplementary Figure 25 – *In vivo* diameter estimation with a piezoelectric transducer in optoacoustic and pulse-echo modes under non-zero offset and yaw angles

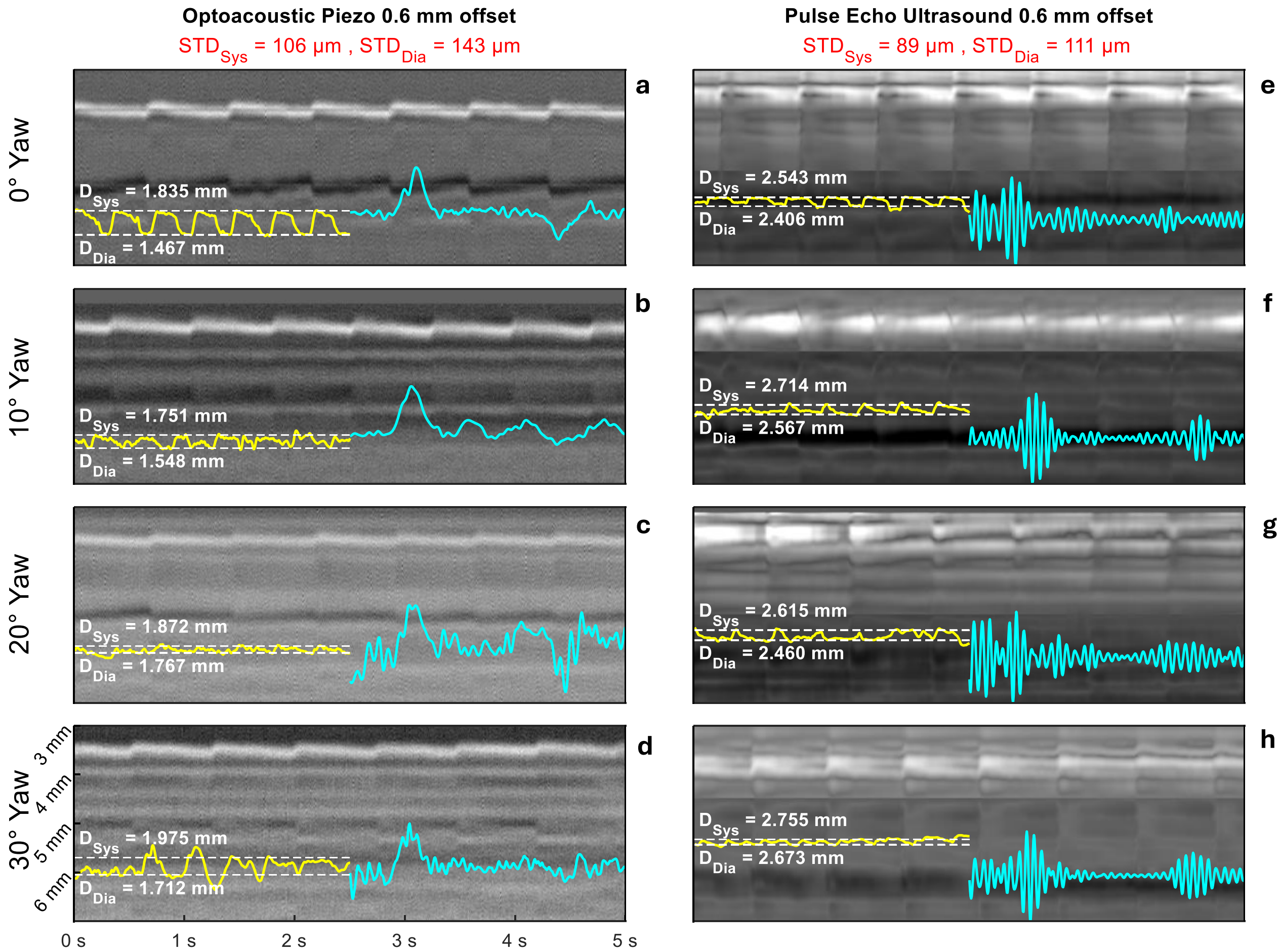

Supplementary Figure 25 – ***In vivo* diameter estimation with a piezoelectric transducer in optoacoustic and pulse-echo modes under non-zero offset and yaw angles**. Diameter estimation of the radial artery in the wrist region of a 29-year-old male obtained using a single piezoelectric element operating in **a-d** optoacoustic and **e-h** pulse-echo modes. The element had a 0.6 mm offset from the center of the radial artery and was rotated with yaw angles up to 30°. Typical raw optoacoustic and pulse-echo signals for each orientation are shown in the blue traces, and the extracted diameter waveforms are shown in the yellow traces.

Supplementary Figure 26 – Photographs of SPADE and the fiber bundle

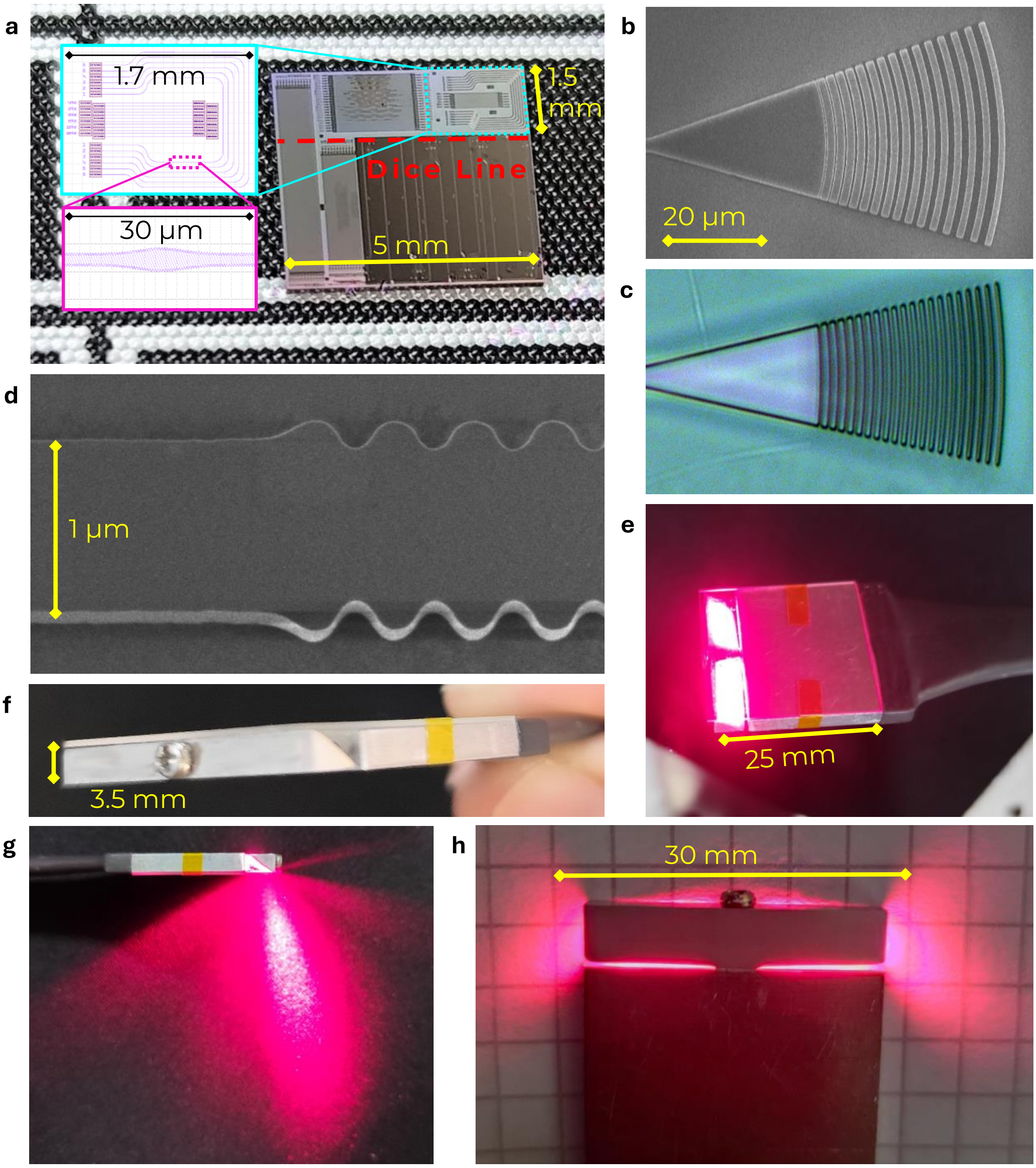

Supplementary Figure 26 – **Photographs of SPADE and the fiber bundle**. **a** The silicon-photonics die used for SPADE (silicon photonics acoustic detector) as received from the MPW (multi-project wafer) fabrication (Ligentec, Switzerland). The dice line and the layout of the sensor region are overlaid on the image. **b** The grating coupler, used for fiber coupling, as seen in a SEM (scanning electron microscopy, S-4700, Hitachi, Japan) and in **c** optical microscopy. **d** The waveguide and its congregations as seen in a SEM. **e** Light emanating from the bundle. **f** A side view of the bundle. **g** The shape of the side-fired beam. **h** The side lobes of the beam extending beyond the device. Note that the light seepage from the bundle-mirror interface is accentuated by the camera, and its intensity is under 50 µJ.

Supplementary Figure 27 – Sensor coupling

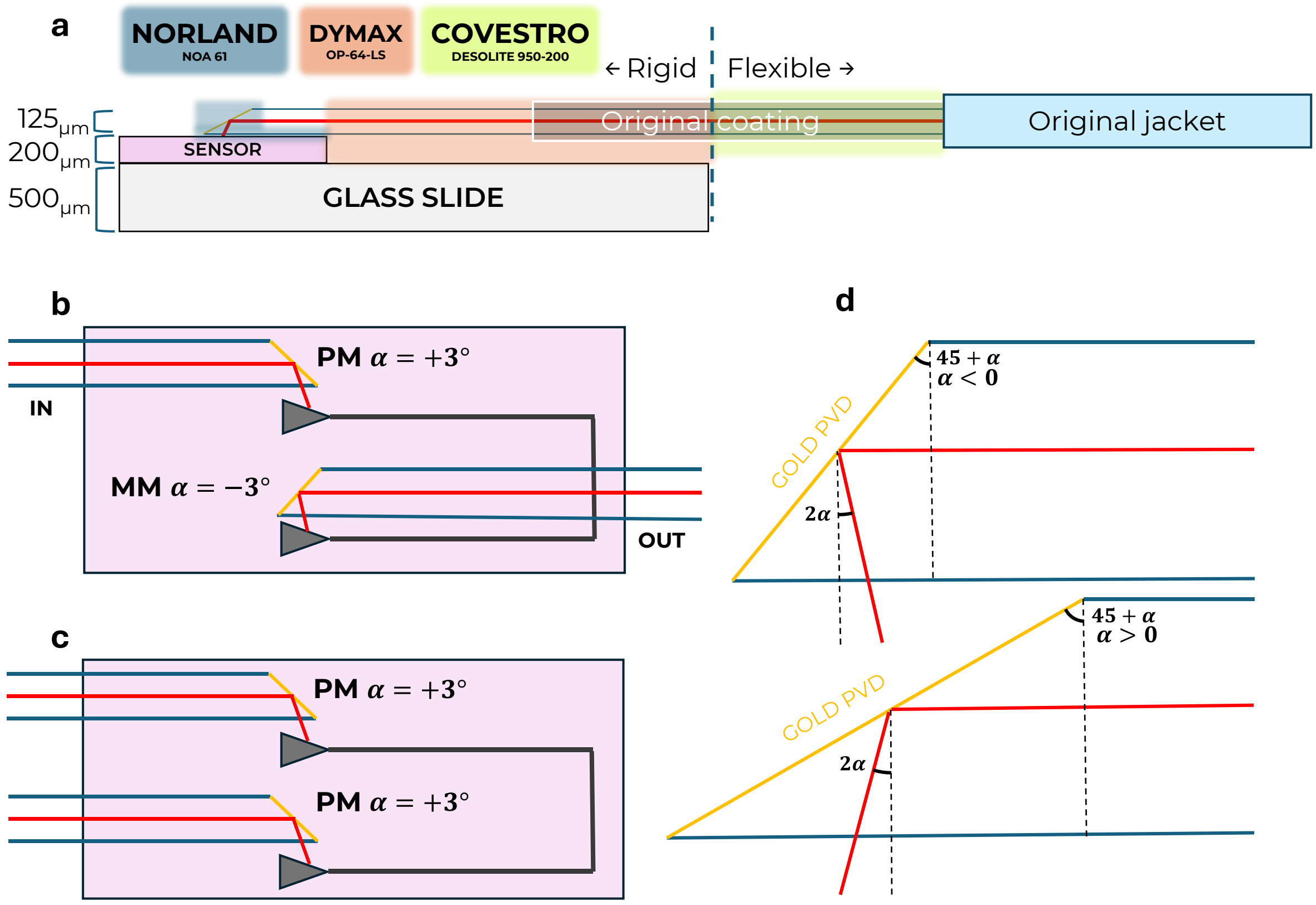

Supplementary Figure 27 – **Sensor coupling**. **a** The mechanical structure of the optoacoustic sensor, showing the different adhesives used to bond its parts. **b** The first step of the coupling, where a MM (multimode) fiber is temporarily used to ease with the positioning of the first PM (polarization maintaining) fiber. **c** The two PM fibers in their final configuration. **d** A schematic of the angles used in polishing the MM and PM fibers.

Supplementary Figure 28 – Photographs of the optoacoustic sensor

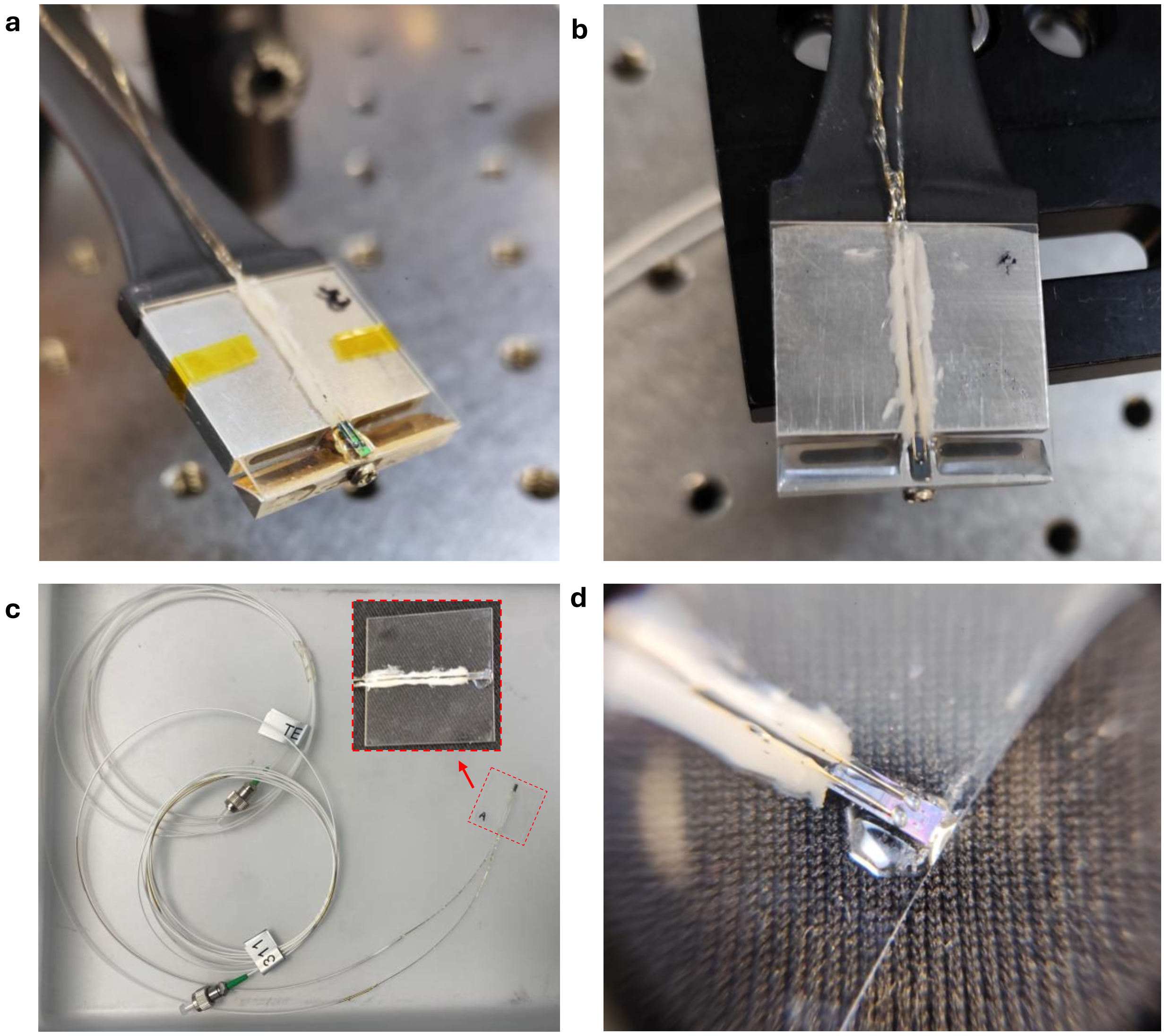

Supplementary Figure 28 - **Photographs of the optoacoustic sensor**. **a** The fully assembled optoacoustic sensor. **b** The optoacoustic sensor without the application of the Kapton tape and before the gold deposition on the mirror. **c** The fiber-coupled SPADE (silicon photonics acoustic detector) before it is attached to the bundle. **d** ×10 magnification of the SPADE, showing the two fibers used for signal readout, and the extra glue protecting their tips.

Supplementary Figure 29 – The SPADE resonator and readout system

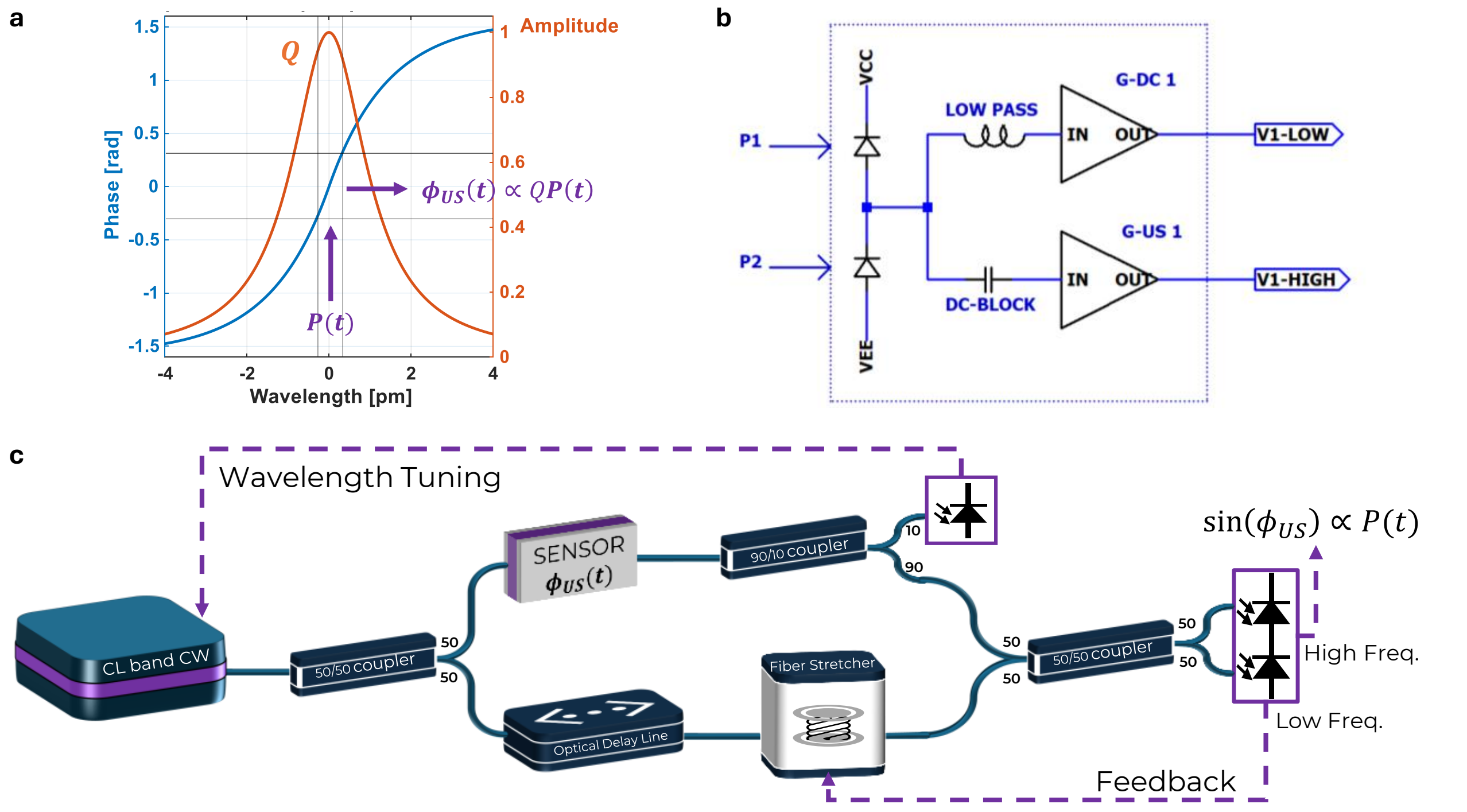

Supplementary Figure 29 – **The SPADE resonator and readout system**. **a** The transmission function of the silicon nitride resonator. The amplitude response is Lorentzian and the phase response`s slope is proportional to the Q-factor. Pressure changes $P(t)$ $[Pa]$ in the vicinity of the sensor induce perturbations that modulate the phase through the sensor $\Phi(t)[rad]$. The modulation efficiency depends on the Q factor and on the acousto-optical properties of the PDMS elastomer cladding. **b** Our bespoke split-band (20 KHz crossover frequency) differential photodetector was designed, manufactured (JLCPCB, China), assembled (Alpha labs, Israel), and tested in house, showing low noise floor and a bandwidth of 250 MHz at -6 dB. It allows for band specific gain values for optimal integration. **c** The SPADE interrogation system: C-L band CW laser (TSL-550, Santec, Japan); PM 1550 Couplers and fibers (AFW Technologies, Australia); Optical delay line (OZ optics, Canada); Fiber stretcher (Paulsson, USA); Photodiode (Thorlabs, USA); The MZI is actively balanced to a quadrature using a feedback circuit driving the fiber stretcher.

Supplementary Figure 30 – Laser stabilization scheme

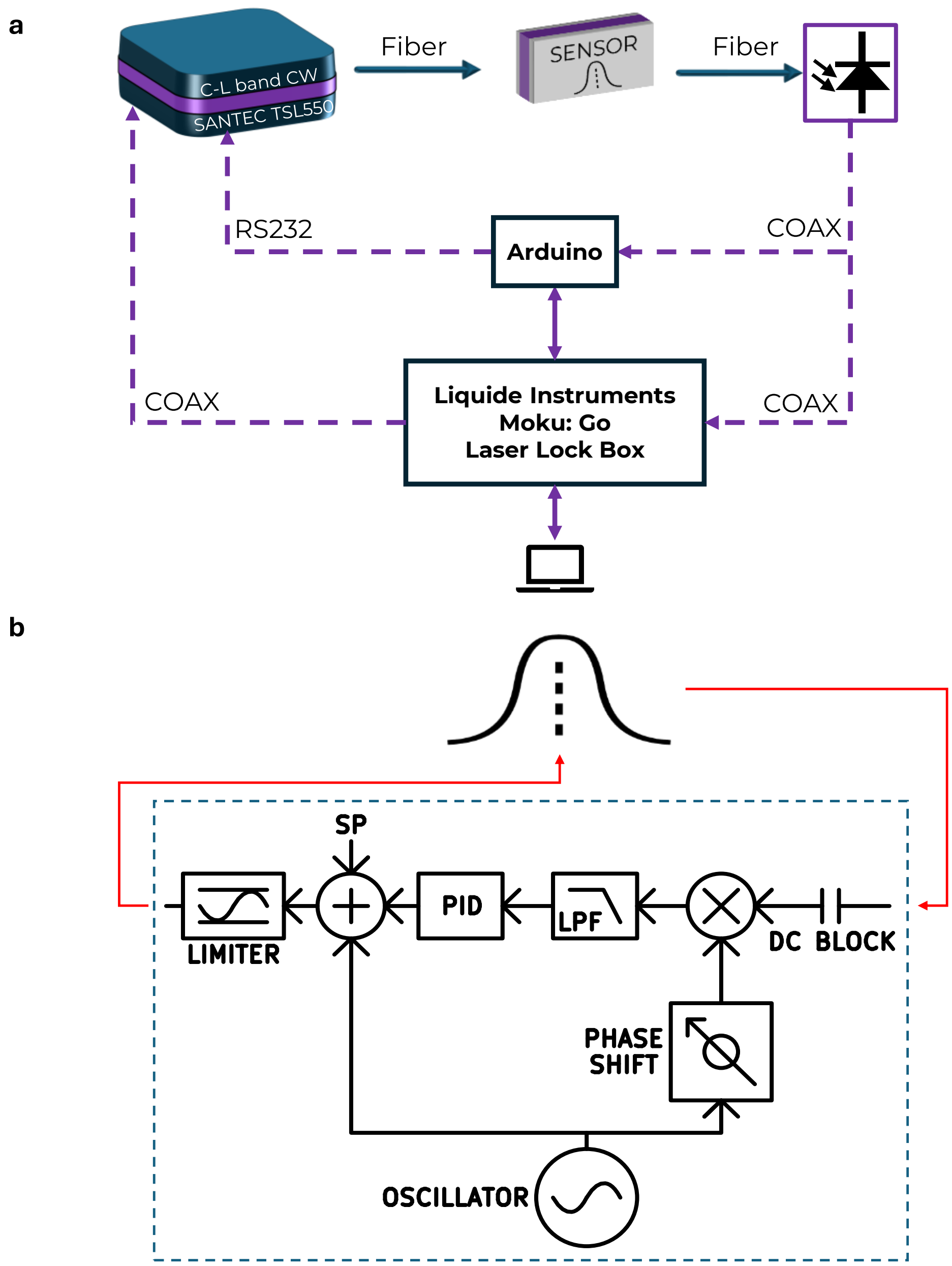

Supplementary Figure 30 – **Laser stabilization scheme**. **a** The stabilization scheme used to the lock the laser to the optical resonance of the SPADE (silicon photonics acoustic detector), including wide-range slow tuning (R4 MINIMA, Arduino, Italy) and narrow-range fast tuning (Moku:Go, Liquide instruments, Australia). **b** A schematic illustration of the laser locking scheme implemented in the Moku:Go, based on the Pound–Drever–Hall (PDH) method. The scheme was implemented in our work with a low frequency modulation of 200 Hz, keeping the sidebands within SPADE's resonance bandwidth, assuring their transmission to the detector.

Supplementary Figure 31 – The Finapres NOVA system

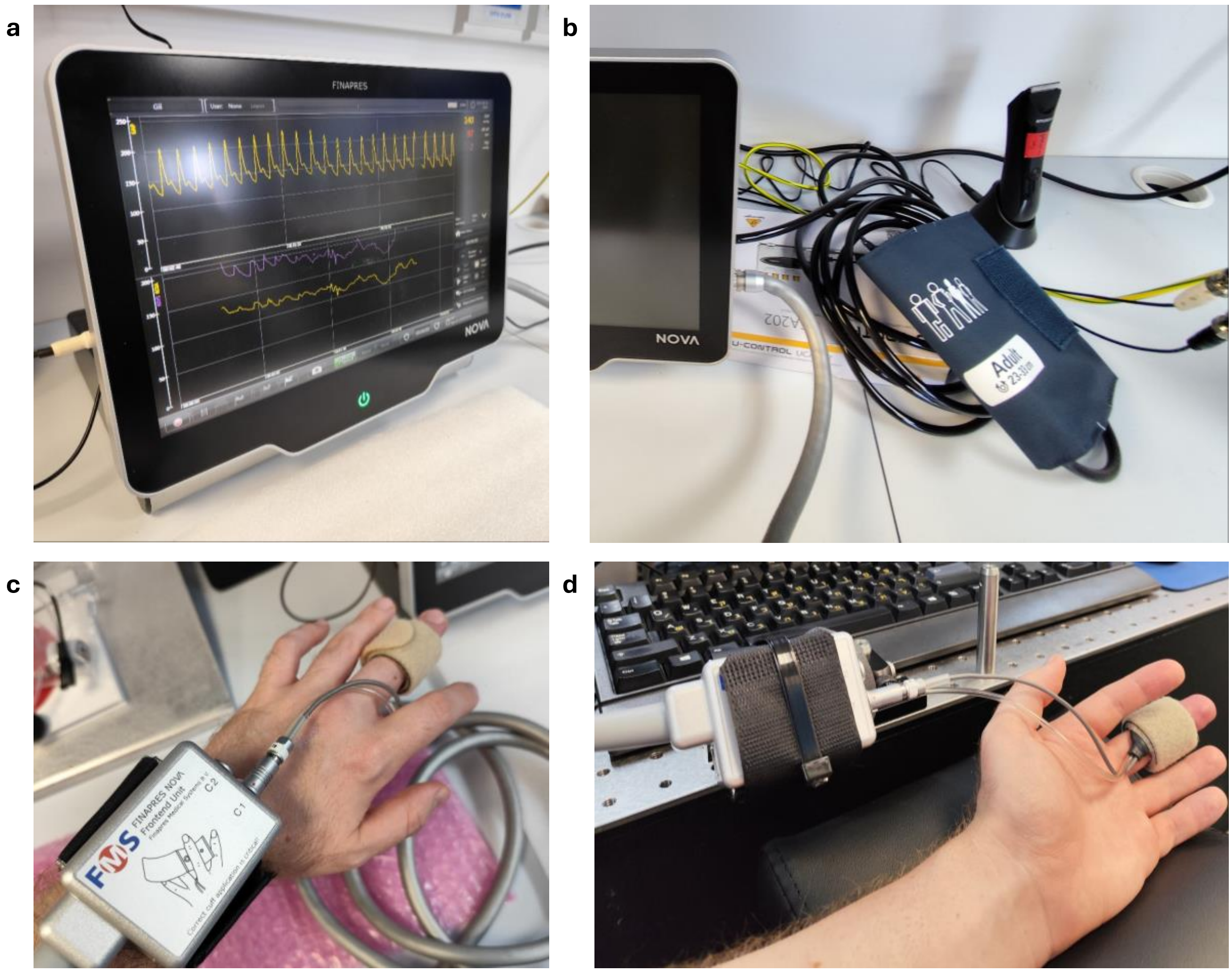

Supplementary Figure 31 - **The Finapres NOVA system**. **a** The system`s main display. **b** The brachial cuff of the NOVA system. **c** The finger cuff of the NOVA system. **d** The finger cuff with the local pump held beside it so as not to block access to the radial artery.

Supplementary Figure 32 – Validation of NOVA brachial BP accuracy

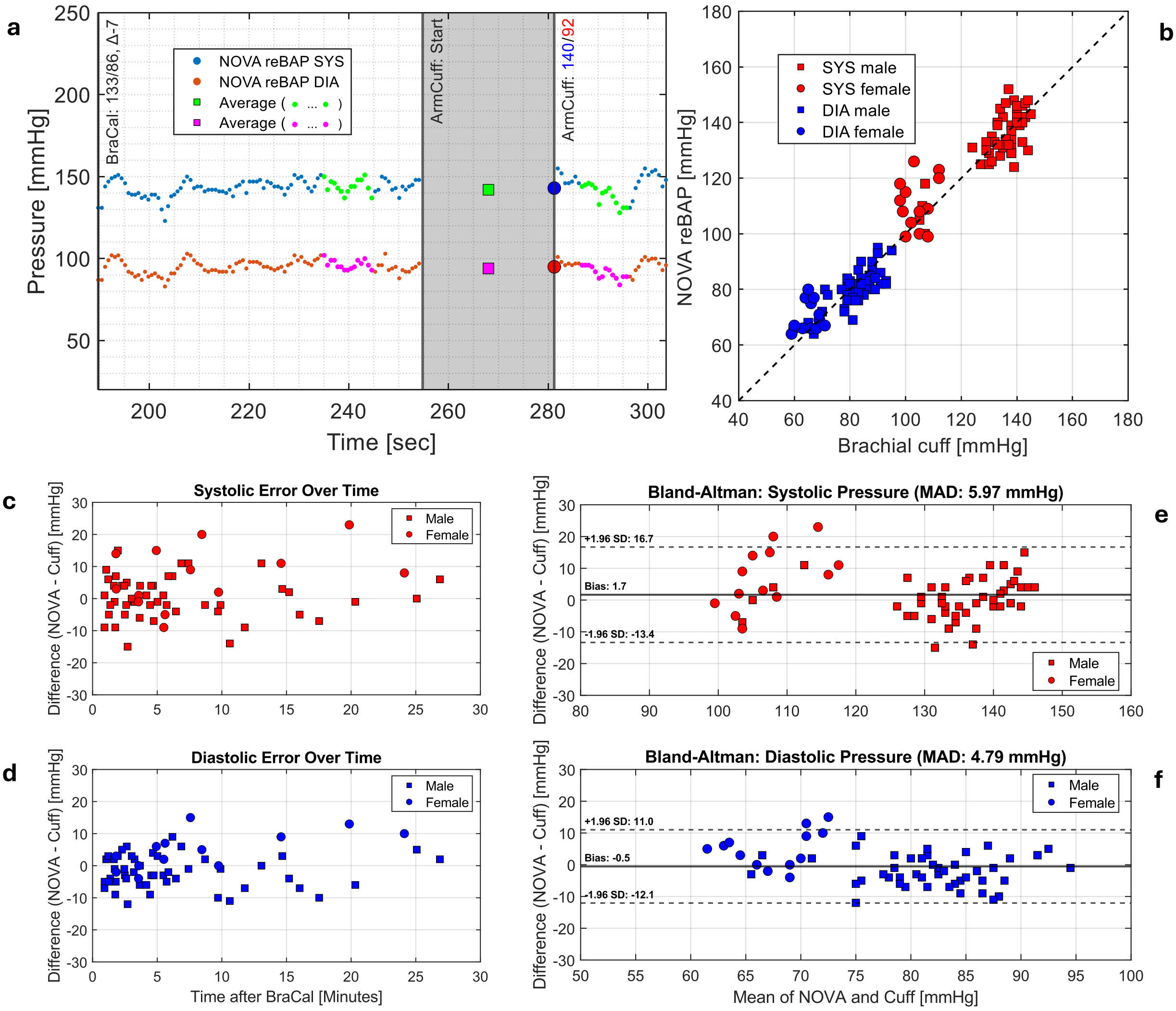

Supplementary Figure 32 – **Validation of NOVA brachial BP accuracy**. **a** Representative evaluation session demonstrating continuous NOVA pressure estimation alongside brachial cuff measurements. Across a one-month period, 16 sessions were conducted with 3 participants (two males, ages 30 and 31; one female, age 22), yielding n = 61 total cuff readings. Sessions began with a brachial cuff calibration (BraCal). Blood pressure variation was induced by alternating between standard seated and seated-core-hold positions. The representative NOVA value for each cuff reading was derived by averaging 20 seconds of continuous data (10 seconds pre-inflation and 10 seconds post-deflation). To mitigate motion artifacts from initiating the reading and the effects of venous congestion, 10-second data buffers immediately preceding inflation and following deflation were excluded, respectively. **b** Correlation scatter plot comparing brachial cuff readings with the NOVA's reconstructed brachial arterial pressure (reBAP). The dashed line represents an ideal 1:1 correlation. **c** Systolic and **d** diastolic estimation errors (NOVA – Cuff) relative to the time elapsed since calibration, demonstrating consistent accuracy over the temporal range utilized in the SPADE clinical study. Bland-Altman plots for **e** systolic and **f** diastolic pressures. The mean absolute difference (MAD) scores were 5.97 mmHg (systolic) and 4.79 mmHg (diastolic), aligning with ISO 81060-2 validation requirements.

Supplementary Figure 33 – Brachial, radial, and finger BP waveforms

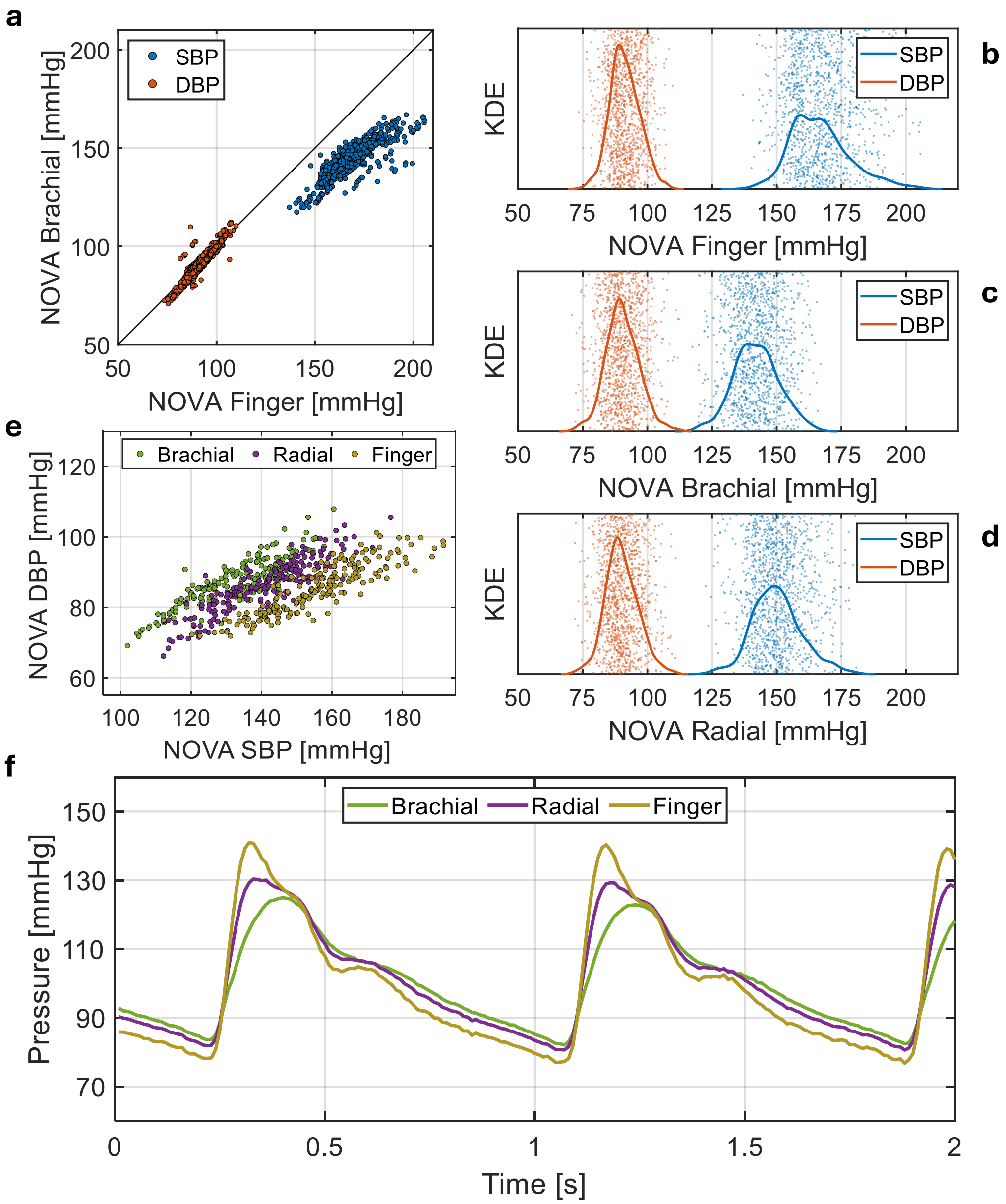

Supplementary Figure 33 – **Brachial, radial, and finger BP waveforms**. **a** A scatter plot showing the relation between the blood pressure (BP) values at the finger and branchial artery, provided by the reBAP function of the NOVA reference system (on a 29-year-old male). Each point (n = 862) is a cardiac cycle. Distributions of the NOVA SBP and DBP values obtained for **b** the finger, **c** brachial artery, and **d** radial artery (generated – Methods). Each point is a cardiac cycle. **e** A scatter plot of the NOVA DBP and SBP values, obtained at the three sites, where each point (n = 186) represents an average over 10 seconds. **f** The BP waveforms at the three sites.

Supplementary Figure 34 – Finger blackout cot

a b c d

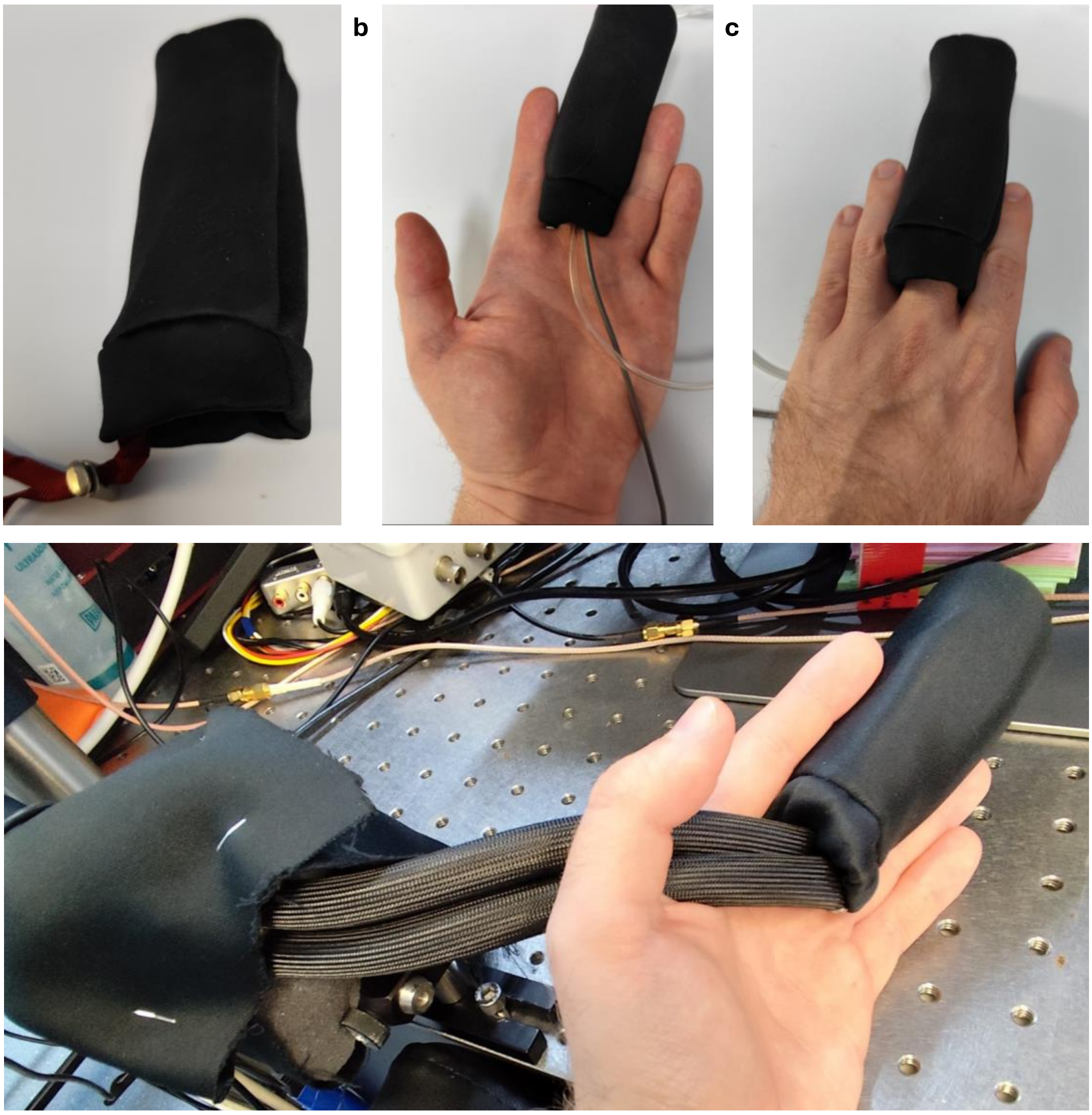

Supplementary Figure 34 – **Finger blackout cot**. **a** The finger cot, used to protect the NOVA`s PPG sensor from stray optoacoustic illumination. **b-c** The finger cot being worn. **d** The optically clear tube and cable connecting the finger cuff are protected by sleeves, and the local pump is covered with laser absorbing cloth (BK5, Thorlabs, USA).

Supplementary Figure 35 – System layout schematic

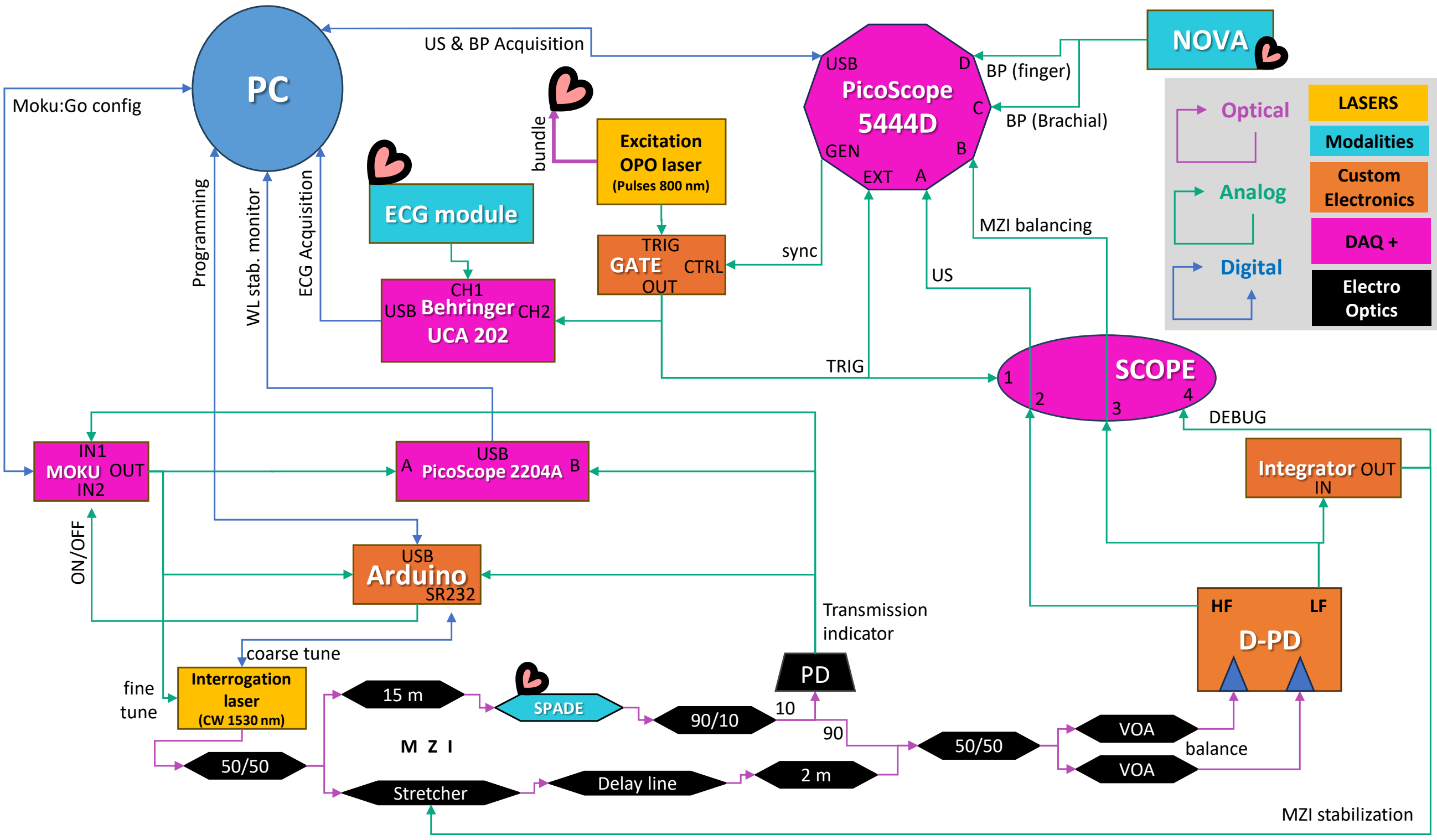

Supplementary Figure 35 – **System layout schematic.** The layout of the system used in the clinical measurements: The ECG module was assembled using the AD8232 (Analog Devices, USA). The bespoke GATE module is a logical AND between its inputs, which is used to synchronize the start of data acquisition across both unsynchronized sampling platforms (PicoScope, UK and Behringer, Germany), ensuring they begin simultaneously only when a measurement control signal is active. The D-PD module is a bespoke split-channel differentiation photodiode transimpedance amplifier. The Integrator module has gain-factor control and is auto-resetting. The VOAs (Optcore, China) are mechanically controlled variable optical attenuators used to equalize the power at the two photodiodes. The scope (DS1104Z+, Rigol, China) is used for monitoring the optoacoustic signal when positioning the device. The excitation OPO laser is the SplitLight EVO I (InnoLas, Germany).

Supplementary Figure 36 – Photographs of the readout system

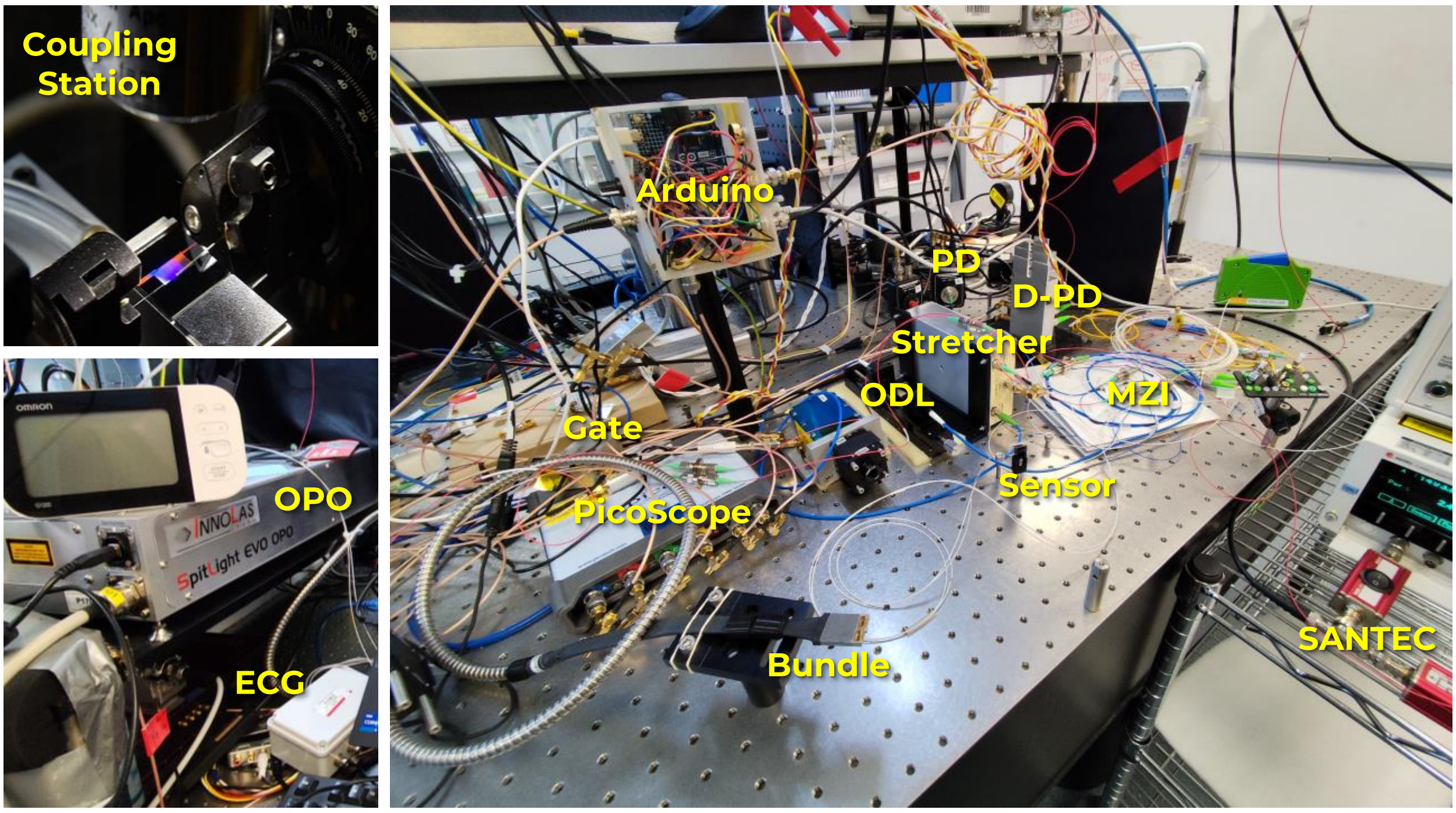

Supplementary Figure 36 – **Photographs of the readout system**. The sensor, excitation & interrogation lasers, and fiber-coupling station are shown.

Supplementary Figure 37 – Data acquisition and synchronization

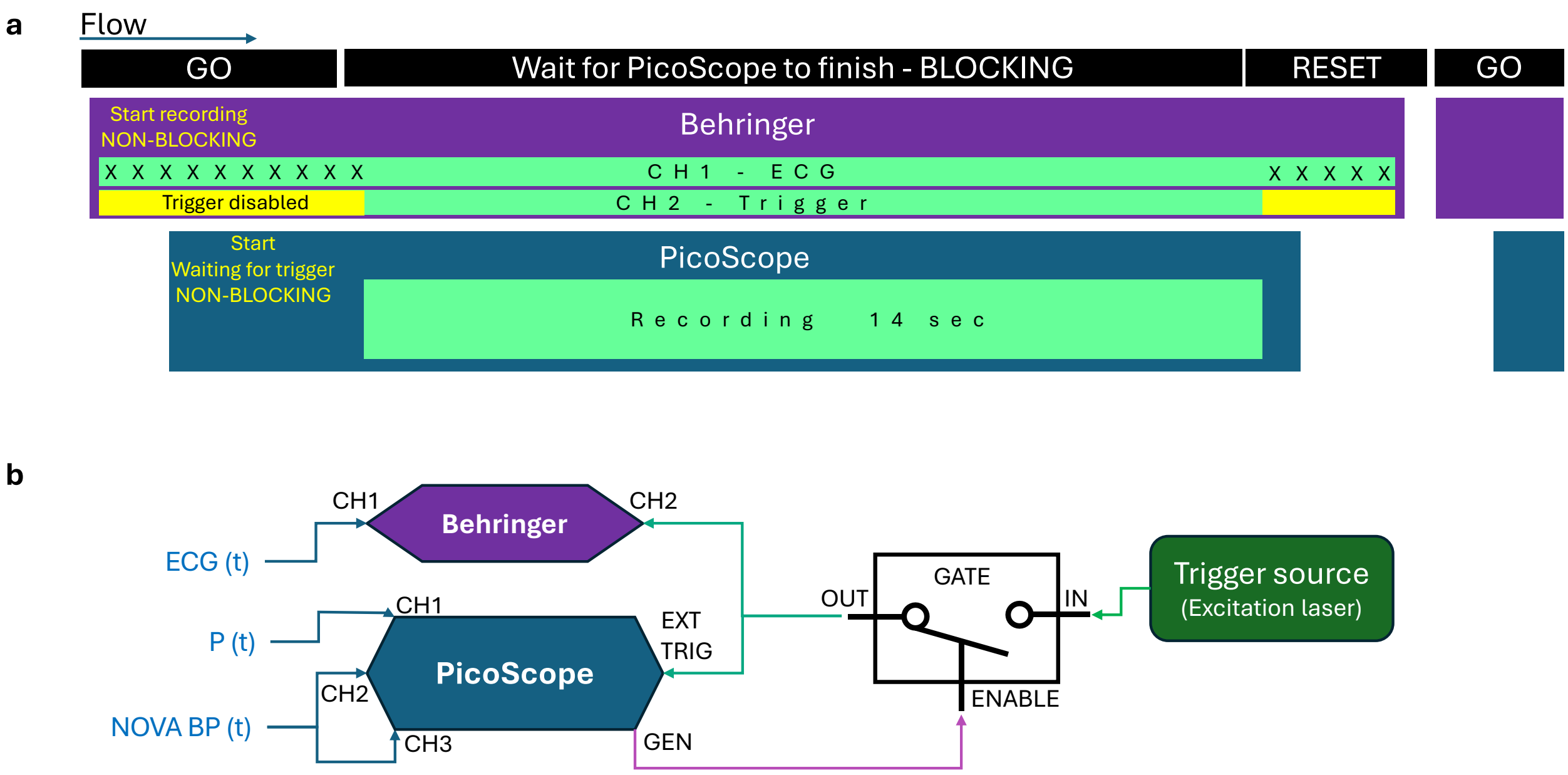

Supplementary Figure 37 – **Data acquisition and synchronization**. **a** The data acquisition and synchronization method of the full experimental setup used in the clinical measurements. The ECG (electrocardiogram) signal is captured by the Behringer (Germany) platform at 192 kHz for improved PAT (pulse arrival time) accuracy, as the sampling of the acoustic signals (PicoScope, UK) operates at the laser repetition rate of 100 Hz. **b** The trigger scheme.

Supplementary Figure 38 – Recording with ECG and extracted PAT

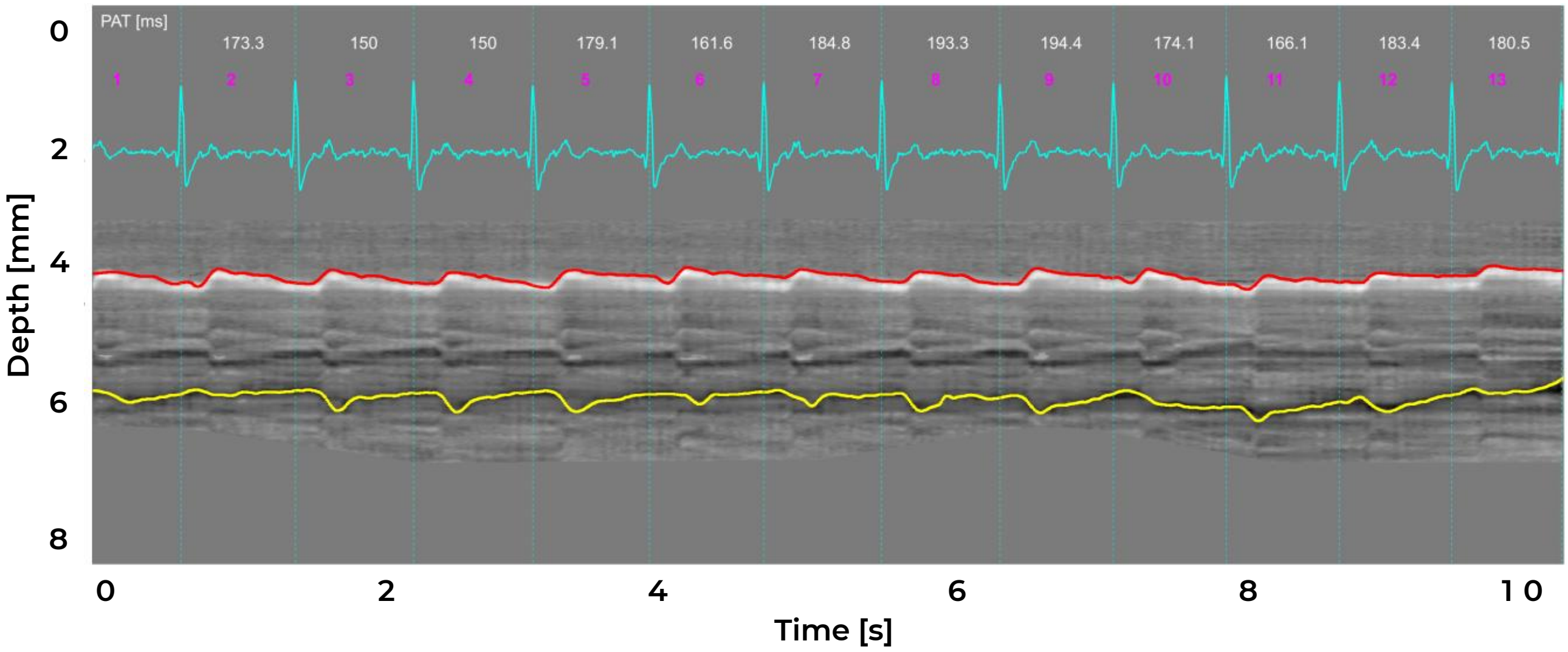

Supplementary Figure 38 - **Recording with ECG and extracted PAT**. Recorded ECG (electrocardiogram) trace overlaid on the M-mode image acquired by the optoacoustic sensor on a 29-year-old male. The time delay between the two signals was used to compute the PAT (pulse arrival time) values, shown in white.

Supplementary Figure 39 – Optoacoustic signal acquisition and processing

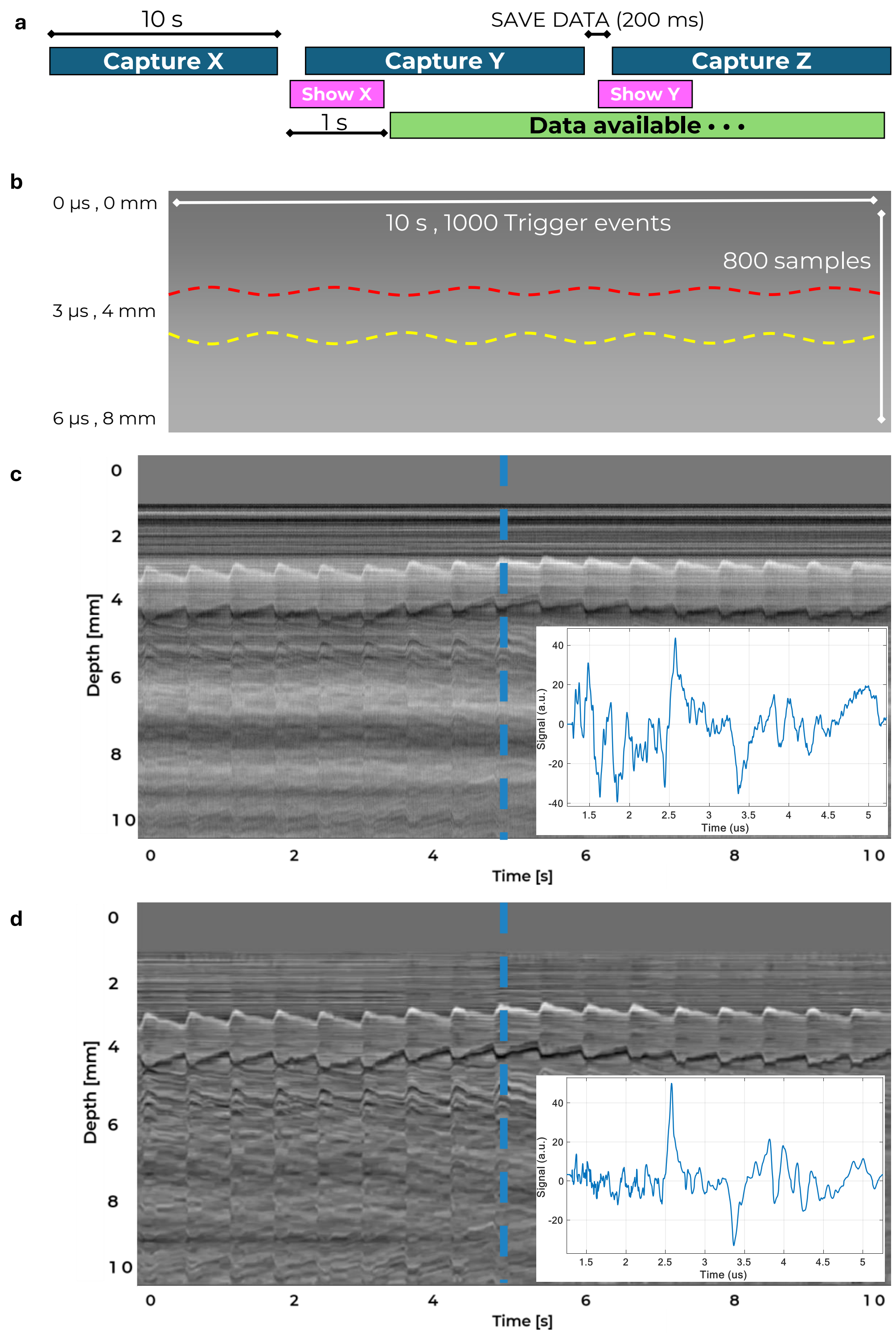

Supplementary Figure 39 - **Optoacoustic signal acquisition and processing**. **a** The data acquisition sequence of the optoacoustic sensor. Blood pressure estimations are put out in blocks of 10 seconds with an intermittent delay after the initial block. **b** The layout of a single data block. **c** M-Mode of SPADE (silicon photonics acoustic detector) signal before post-processing. **d** M-Mode of SPADE signal after post-processing (Methods).

Supplementary Figure 40 – Bandwidth-limited diameter estimation

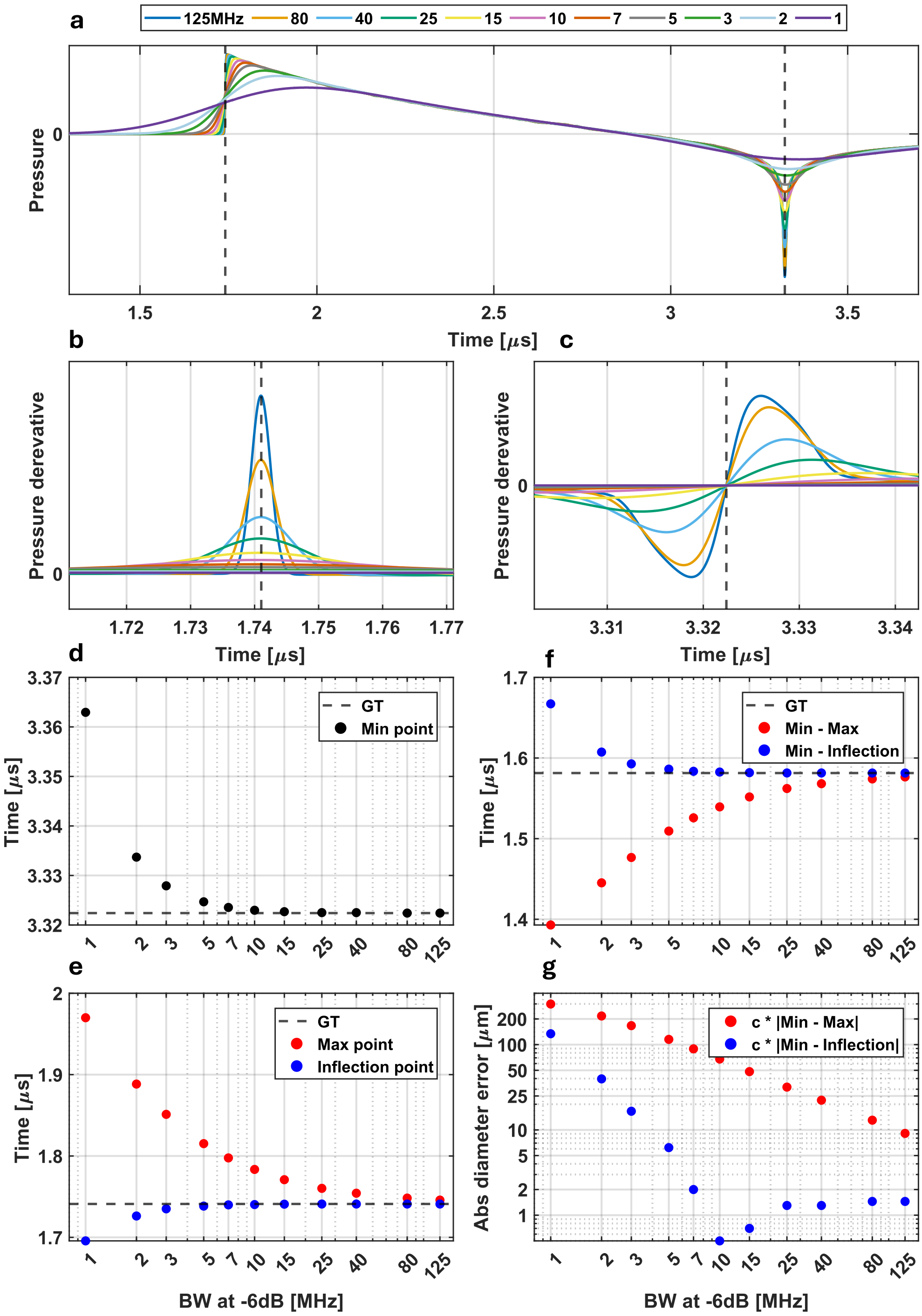

Supplementary Figure 40 – **Bandwidth-limited diameter estimation**. **a** The effect of lowpass filtering on the optoacoustic signal from a non-ideal cylindrical source. The time derivative of the low-passed optoacoustic signal for the **b** anterior and **c** posterior ends. The effect of bandwidth (BW) on the timing of the **d** minimum point and **e** maximum and inflection points. **f** The time difference between the points compared to ground truth (GT) as a function of BW. **g** The diameter estimation error as a function of system BW, showing that the Min-Inflection approach remains accurate to within 3 μm for a system with -6 dB bandwidth over 6 MHz.

Supplementary Figure 41 – Bandwidth changes with offset

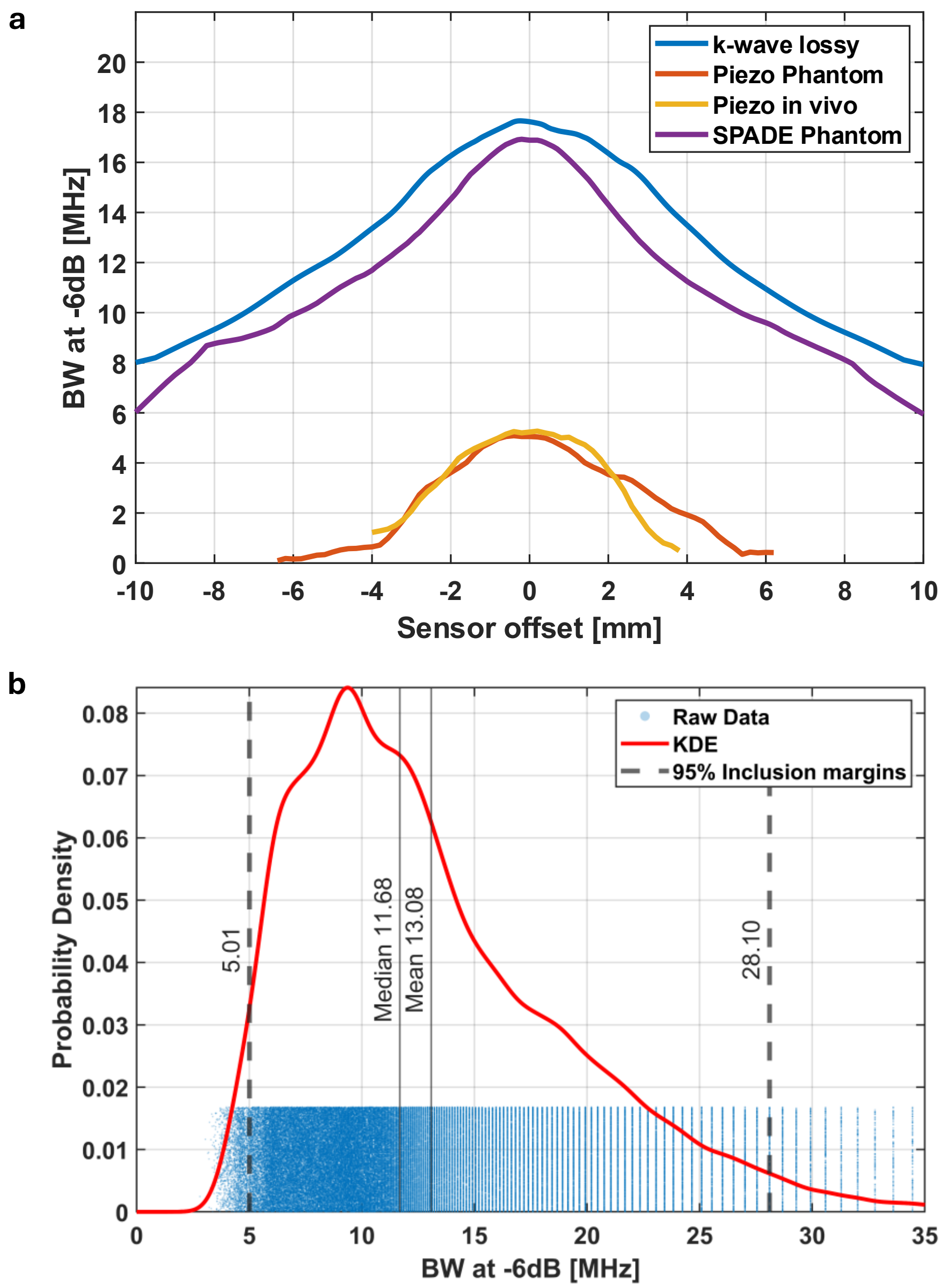

Supplementary Figure 41 – **Bandwidth changes with offset**. **a** The effective bandwidth (BW) of different signals acquired, calculated based on the leading step rise time in the optoacoustic signal. The k-wave plot refers to the simulated optoacoustic signal as was described in Supplementary Note 8 for typical depth and acoustic attenuation in tissue. The two piezo plots refer to the piezoelectric optoacoustic imager described in Supplementary Note 13. The bandwidth decay profile of the piezoelectric transducers is dominated by their limited acceptance angle, rather than the increase in acoustic path. The SPADE phantom plot refers to the experiment described in Supplementary Note 11. In all the plots, the yaw and pitch angles were 0°. **b** A distribution of the bandwidth achieved in the *in vivo* study with the SPADE based optoacoustic probe. Each count is a single trigger event, and a total of n = 114,000 events are shown from 90 randomly selected measurements (male and female aggregated). Counts exceeding 20 MHz can be attributed to times when the artery was particularly shallow and with minimal offset, whereas counts below 6 MHz (7% of the measurements) can be attributed to the opposite scenario.

Supplementary Figure 42 – Bandwidth and resolution of sensors

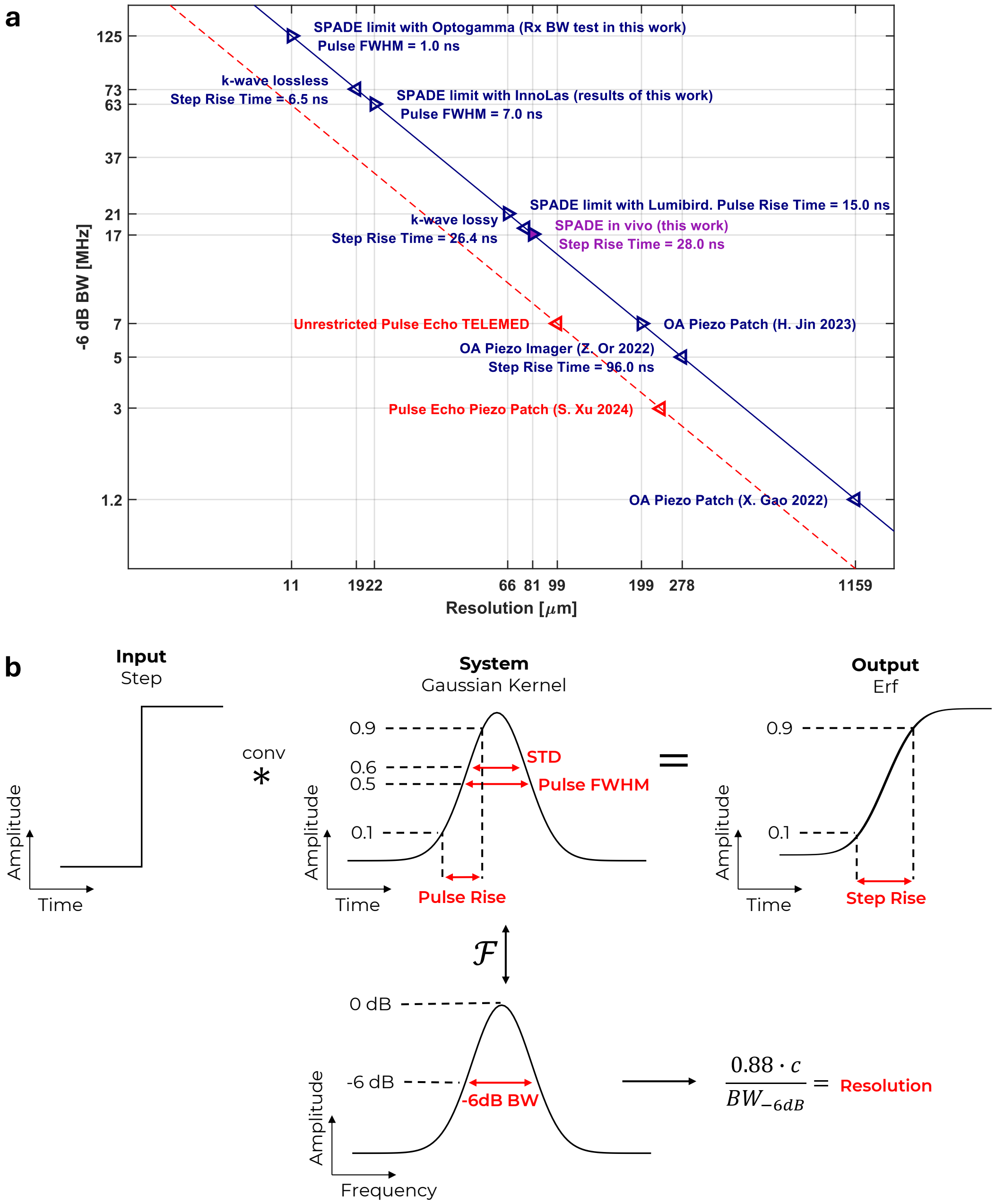

Supplementary Figure 42 – **Bandwidth and resolution of sensors**. **a** Comparison of acoustic sensors from the literature, alongside limitations imposed by the different excitation laser technologies: a Q-switched laser (WAVEGUARD-D, Optogama, Lithuania), an OPO source (SplitLight EVO I , InnoLas, Germany), and laser diodes (QD-Q1910-ILO, Lumibird, France). While the Lumibird laser has a nominal pulse FWHM of 50 ns, its pulse shape is asymmetric; its rise time is only 15 ns, making its effective FWHM equal to 20 ns within the context of optoacoustic excitation. In addition, simulative predictions obtained by k-wave are shown for realistic acoustic attenuation parameters. Systems operating in pulse-echo ultrasound mode lie on the red dashed line, whereas optoacoustic systems lie on the blue solid line. Because pulse-echo ultrasound inherently achieves twice the axial resolution of optoacoustics for a given bandwidth, the two lines are shifted but parallel. The axes are plotted on a log-log scale. **b** Schematic illustration of the temporal and spectral properties used to derive this plot (highlighted in red). These Gaussian-based analytical relationships are detailed in Supplementary Table 5.

Supplementary Figure 43 – Photographs of the optoacoustic sensor as a wearable device

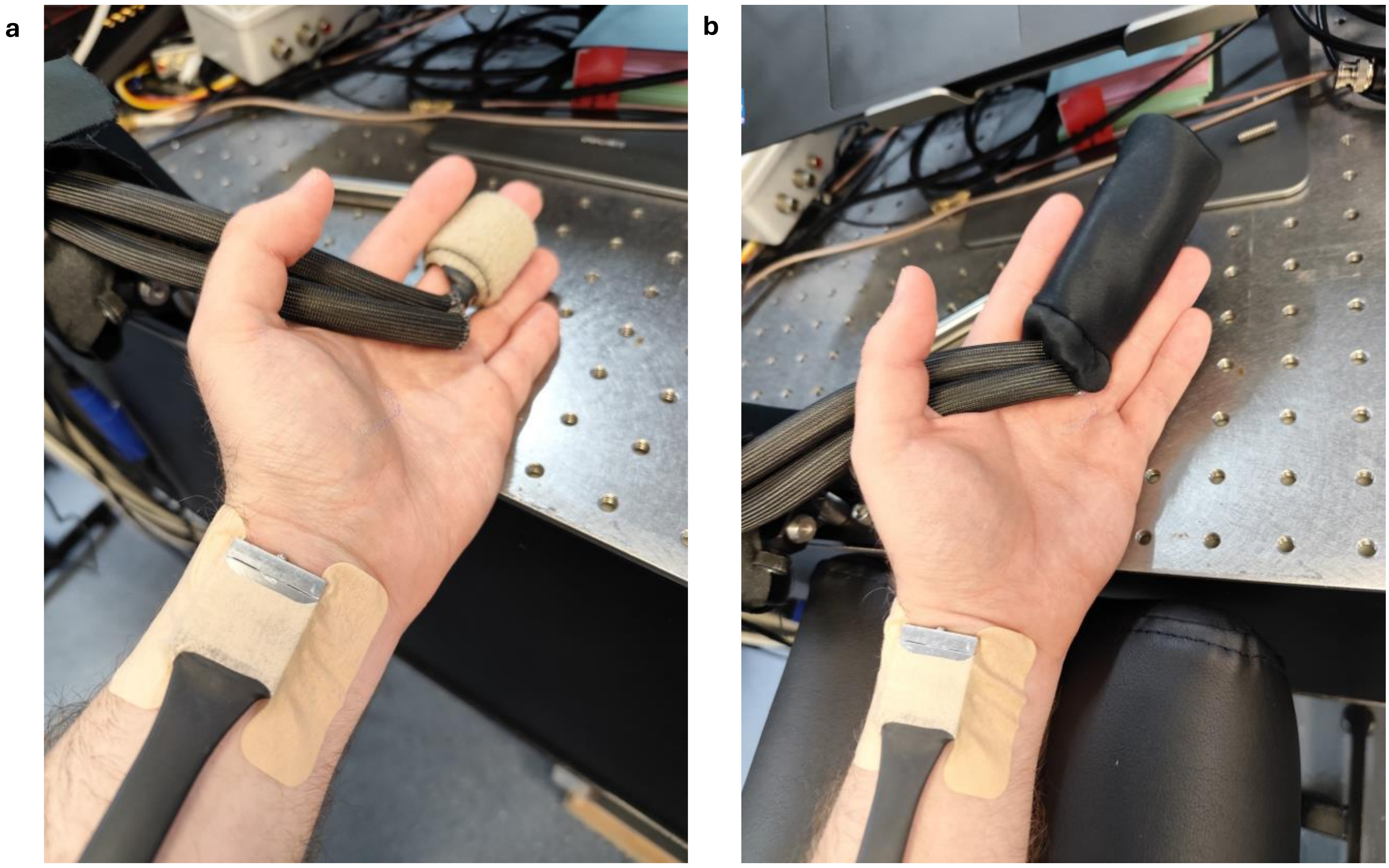

Supplementary Figure 43 – **Photographs of the optoacoustic sensor as a wearable device**. The optoacoustic sensor being worn on the arm together with the reference BP (blood pressure) cuff worn on the finger, **a** without the cot, used to protect the NOVA` s PPG sensor from stray optoacoustic light, and **b** with the cot.

Supplementary Figure 44 – Inducing blood pressure changes

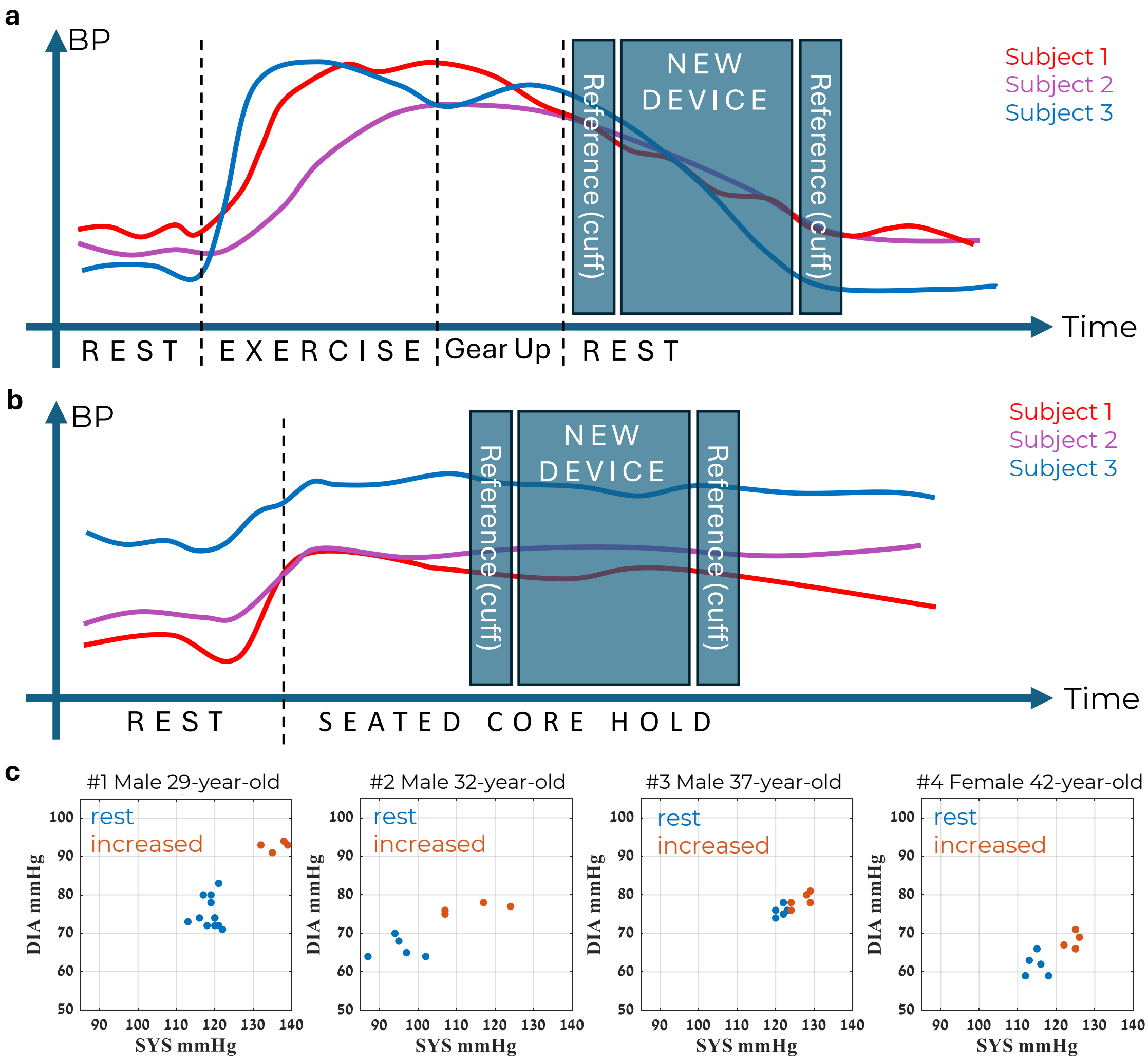

Supplementary Figure 44 - **Inducing blood pressure changes**. **a** Problems with exercise induced BP (blood pressure) changes. **b** The seated-core-hold position. **c** Demonstration of the effectiveness of the seated core hold position, measured with the M500 (Omron, Japan). The amount the BP increases is individual and related to individual physical shape. In some cases, the increase is modest. Even then, the variability does not exceed the baseline. Thus, although the resulting BP values may not rise substantially, degrading the system's overall error performance is avoided.

Supplementary Figure 45 – Additional BP estimation plots

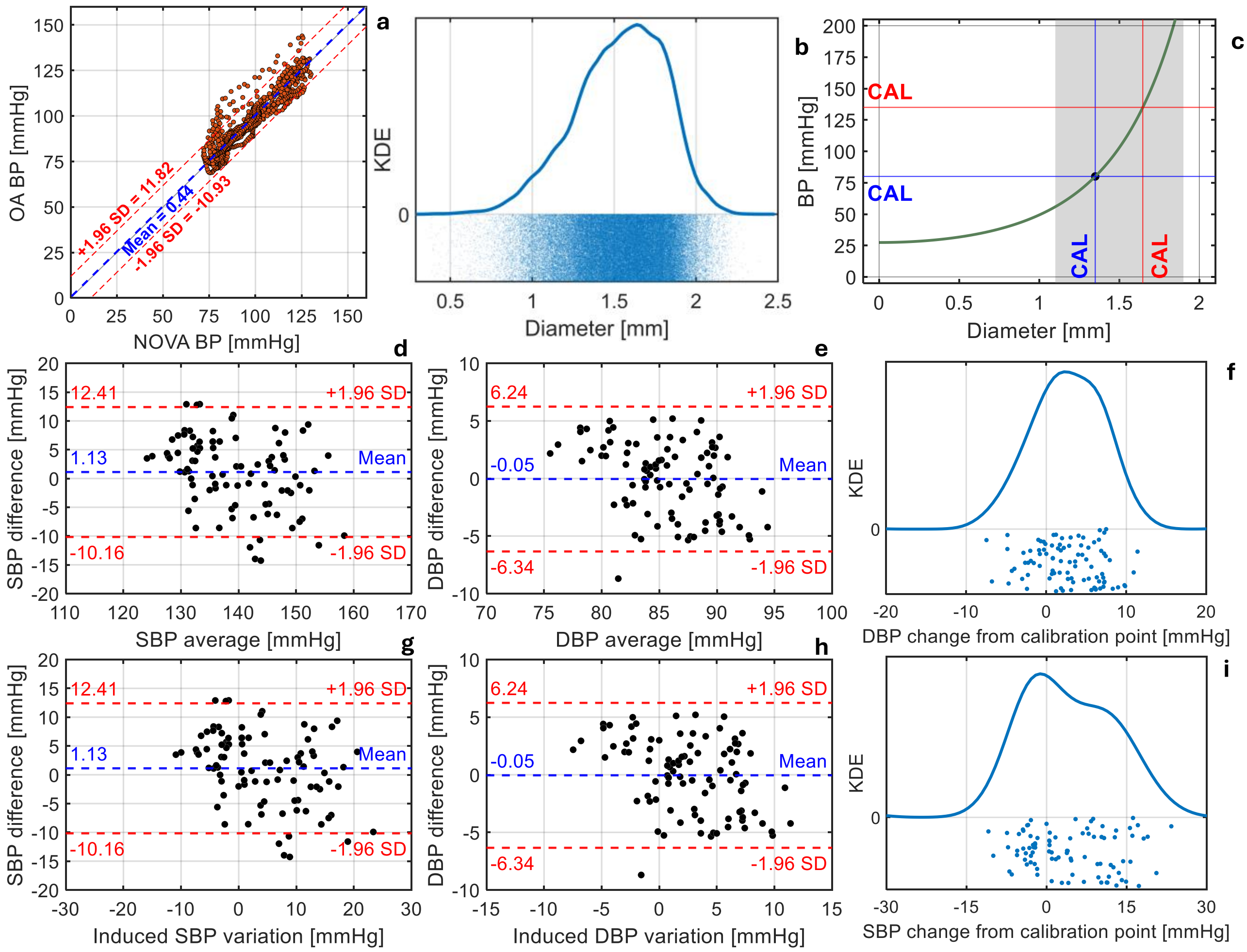

Supplementary Figure 45 – **Additional BP estimation plots**. **a** n = 1,000 estimation points from a single 10-second reading are scattered, showing good correlation not only for their extremal values, but for the entire waveform. **b** A distribution of diameter readings, counted per trigger event (n = 91,000). **c** The BP-diameter curve of the model, whose coefficients are calculated from the SBP and DBP values from NOVA, and from the systolic and diastolic diameters from the optoacoustic sensor. The diastolic pair is kept, while the systolic pair is expressed in beta (the slope). **d** Bland-Altman plot for the SBP estimation. **e** Bland-Altman plot for the DBP estimation. **f** A distribution of DBP reading difference from the calibration point. **g** Bland-Altman plot for the induced SBP variation from the calibration point. **h** Bland-Altman plot for the induced DBP variation from the calibration point. **i** A distribution of SBP reading difference from the calibration point. This figure only contains measurements performed on the 29-year-old male subject (n = 91 for d-i).

Supplementary Figure 46 – Pulse-echo diameter estimation with planewave Tx / individual element Rx

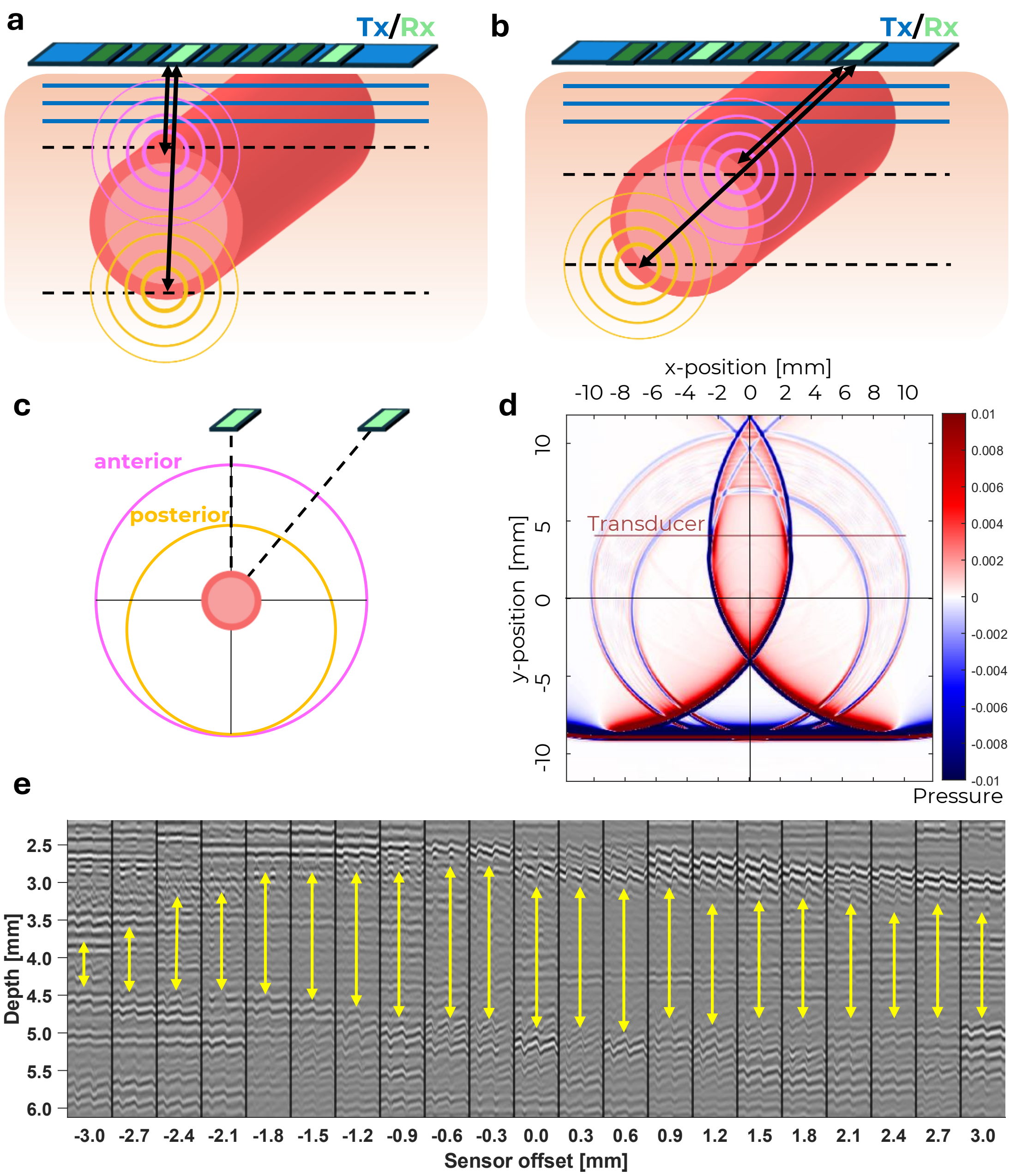

Supplementary Figure 46 – **Pulse-echo diameter estimation with planewave Tx / individual-element Rx**. The choice of Rx element offset changes the perceived diameter: In **a**, the minimal-offset element yields a correct reading, while the higher offset element in **b** yields a smaller one, marked by the dashed lines. The spatial propagation of the anterior and posterior echoes is illustrated in **c**, and simulated in **d**. An *in vivo* measurement (radial artery near the wrist of a 30-year-old male) of this operation mode is presented in **e**, where 2-second segments from the central elements are concatenated without processing.

Supplementary Figure 47 – Pulse-echo diameter estimation with Tx/Rx using planewaves

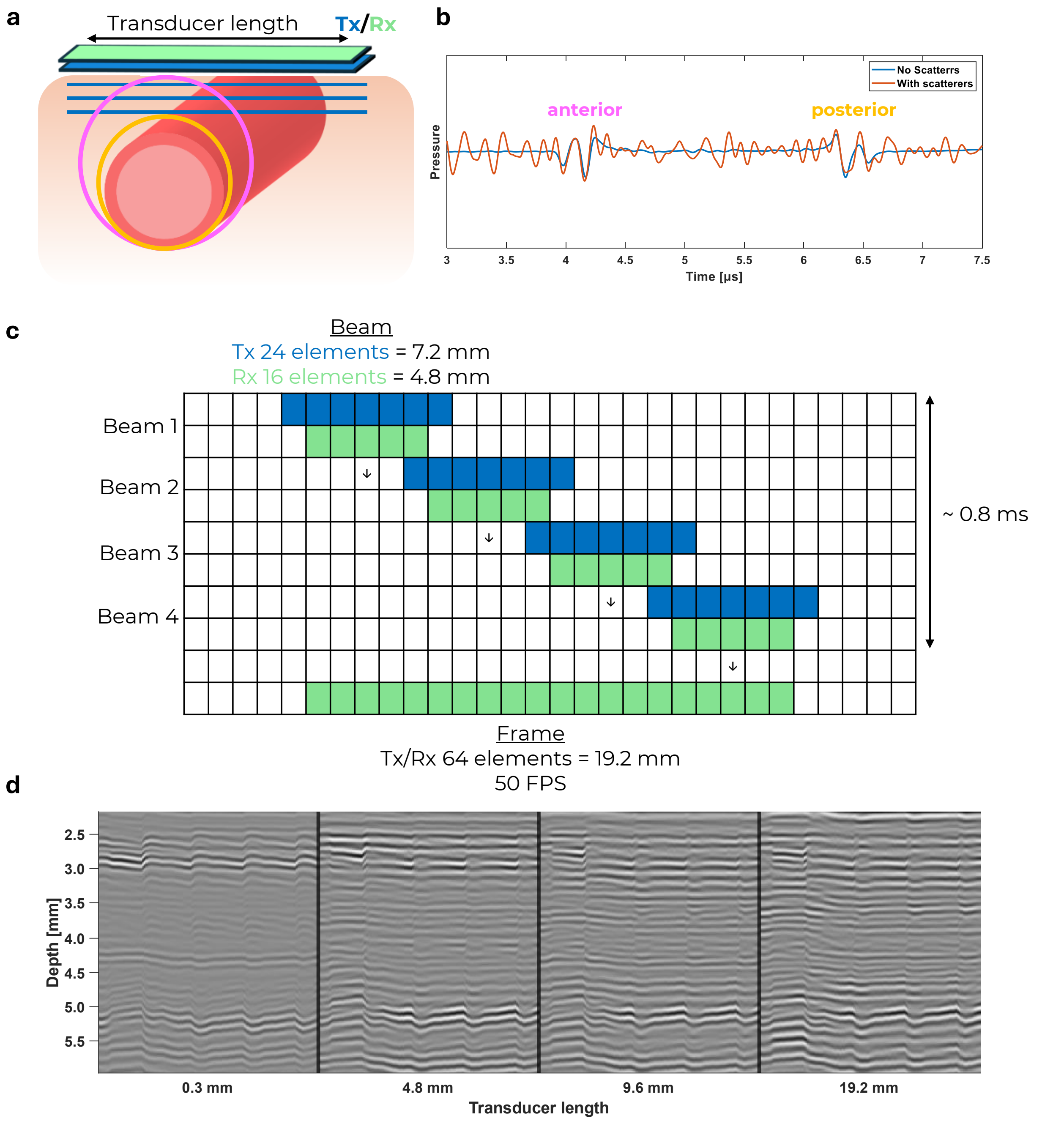

Supplementary Figure 47 – **Pulse-echo diameter estimation with Tx/Rx using planewaves**. **a** An illustration of the planewave Tx/Rx mode of operation. **b** A k-Wave simulation of the planewave Tx/Rx mode, with and without acoustic scatters included. **c** An illustration of the Telemed acquisition scheme. **d** *In vivo* demonstration (radial artery near the wrist of a 30-year-old male) of the mode for different transducer lengths with 4-second segments, showing that the signal to background ratio drops when transitioning from a single sensor to a plane wave Rx.

Supplementary References

1. Westerhof, N., Stergiopulos, N., Noble, M. I. M. & Westerhof, B. E. *Snapshots of Hemodynamics: An Aid for Clinical Research and Graduate Education*. (Springer International Publishing, Cham, 2019). doi:10.1007/978-3-319-91932-4.
2. Muntner, P. *et al*. Measurement of Blood Pressure in Humans: A Scientific Statement From the American Heart Association. *Hypertension* **73**, (2019).
3. Lewis, G. D. *et al*. Exercise Blood Pressure and the Risk of Incident Cardiovascular Disease (from the Framingham Heart Study). *The American Journal of Cardiology* **101**, 1614–1620 (2008).
4. Sharman, J. E. & LaGerche, A. Exercise blood pressure: clinical relevance and correct measurement. *J Hum Hypertens* **29**, 351–358 (2015).
5. McEniery, C. M., Cockcroft, J. R., Roman, M. J., Franklin, S. S. & Wilkinson, I. B. Central blood pressure: current evidence and clinical importance. *European Heart Journal* **35**, 1719–1725 (2014).
6. Juraschek, S. P. *et al*. Comparison of supine and seated orthostatic hypotension assessments and their association with falls and orthostatic symptoms. *J American Geriatrics Society* **70**, 2310–2319 (2022).
7. Fei Li, Ling Wang, Yubo Fan, & Deyu Li. Simulation of noninvasive blood pressure estimation using ultrasound contrast agent microbubbles. *IEEE Trans. Ultrason., Ferroelect., Freq. Contr.* **59**, 715–726 (2012).
8. Tremblay-Darveau, C., Williams, R. & Burns, P. N. Measuring Absolute Blood Pressure Using Microbubbles. *Ultrasound in Medicine & Biology* **40**, 775–787 (2014).
9. Andersen, K. S. & Jensen, J. A. Non-invasive estimation of blood pressure using ultrasound contrast agents. *Physics Procedia* **3**, 245–253 (2010).
10. IEEE Standard for Wearable Cuffless Blood Pressure Measuring Devices. doi:10.1109/IEEESTD.2014.6882122.
11. IEEE Standard for Wearable, Cuffless Blood Pressure Measuring Devices - Amendment 1. doi:10.1109/IEEESTD.2019.8859685.
12. Fang, J. *et al*. Perspectives of Global Standards on Validation of Blood Pressure Measuring Devices. *IEEE Trans. Biomed. Eng.* 1–15 (2025) doi:10.1109/TBME.2025.3587411.
13. International Organization for Standardization. *Non-Invasive Sphygmomanometers — Part 3: Clinical Investigation of Continuous Automated Measurement Type*. https://www.iso.org/standard/71161.html (2022).
14. Duck, F. A. *Physical Properties of Tissue: A Comprehensive Reference Book*. (Academic Press, London, 1990).
15. Kireev, D. *et al*. Continuous cuffless monitoring of arterial blood pressure via graphene bioimpedance tattoos. *Nat. Nanotechnol.* **17**, 864–870 (2022).
16. Gholamhosseini, H., Baig, M., Rastegar, S. & Lindén, M. Cuffless Blood Pressure Estimation Using Pulse Transit Time and Photoplethysmogram Intensity Ratio. *Stud Health Technol Inform* **249**, 77–83 (2018).
17. *The Handbook of Cuffless Blood Pressure Monitoring: A Practical Guide for Clinicians, Researchers, and Engineers*. (Springer International Publishing, Cham, 2019). doi:10.1007/978-3-030-24701-0.
18. Ma, Y. *et al*. Relation between blood pressure and pulse wave velocity for human arteries. *Proc. Natl. Acad. Sci. U.S.A.* **115**, 11144–11149 (2018).
19. Min, S. *et al*. Wearable blood pressure sensors for cardiovascular monitoring and machine learning algorithms for blood pressure estimation. *Nat Rev Cardiol* https://doi.org/10.1038/s41569-025-01127-0 (2025) doi:10.1038/s41569-025-01127-0.
20. Hayashi, K., Handa, H., Nagasawa, S., Okumura, A. & Moritake, K. Stiffness and elastic behavior of human intracranial and extracranial arteries. *Journal of Biomechanics* **13**, 175–184 (1980).
21. Meinders, J. M. & Hoeks, A. P. G. Simultaneous assessment of diameter and pressure waveforms in the carotid artery. *Ultrasound in Medicine & Biology* **30**, 147–154 (2004).

22. Stefanadis, C. *et al.* Pressure-Diameter Relation of the Human Aorta: A New Method of Determination by the Application of a Special Ultrasonic Dimension Catheter. *Circulation* **92**, 2210–2219 (1995).
23. Zhou, S. *et al.* Clinical validation of a wearable ultrasound sensor of blood pressure. *Nat. Biomed. Eng* **9**, 865–881 (2024).
24. Hughes, D. J., Babbs, C. F., Geddes, L. A. & Bourland, J. D. Measurements of Young's Modulus of Elasticity of the Canine Aorta with Ultrasound.
25. Ding, X.-R. & Zhang, Y.-T. Photoplethysmogram intensity ratio: A potential indicator for improving the accuracy of PTT-based cuffless blood pressure estimation. *Annu Int Conf IEEE Eng Med Biol Soc* **2015**, 398–401 (2015).
26. Peter, L., Noury, N. & Cerny, M. A review of methods for non-invasive and continuous blood pressure monitoring: Pulse transit time method is promising? *IRBM* **35**, 271–282 (2014).
27. Hennig, A. & Patzak, A. Continuous blood pressure measurement using pulse transit time. *Somnologie* **17**, 104–110 (2013).
28. Liu, A.-B., Hsu, P.-C., Chen, Z.-L. & Wu, H.-T. Measuring pulse wave velocity using ECG and photoplethysmography. *J Med Syst* **35**, 771–777 (2011).
29. Nabeel, P. M., Jayaraj, J., Srinivasa, K., Mohanasankar, S. & Chenniappan, M. Bi-Modal Arterial Compliance Probe for Calibration-Free Cuffless Blood Pressure Estimation. *IEEE Trans. Biomed. Eng.* **65**, 2392–2404 (2018).
30. Garrett, A. *et al.* Simultaneous photoplethysmography and blood flow measurements towards the estimation of blood pressure using speckle contrast optical spectroscopy. *Biomed. Opt. Express* **14**, 1594 (2023).
31. Einalou, Z. *et al.* Noninvasive continuous blood pressure prediction using FlexNIRS and machine learning during carotid endarterectomy. *J. Biomed. Opt.* **30**, (2025).
32. Hsiao, C., Hong, S., Branan, K. L., McMurray, J. & Cote, G. L. Predicting Blood Pressure Without a Cuff Using a Unique Multi-Modal Wearable Device and Machine Learning Algorithm. Preprint at https://doi.org/10.2139/ssrn.4967879 (2024).
33. Parati, G., Ochoa, J. E., Lombardi, C. & Bilo, G. Assessment and management of blood-pressure variability. *Nat Rev Cardiol* **10**, 143–155 (2013).
34. Wang, C. *et al.* Monitoring of the central blood pressure waveform via a conformal ultrasonic device. *Nat Biomed Eng* **2**, 687–695 (2018).
35. Lin, M. *et al.* A fully integrated wearable ultrasound system to monitor deep tissues in moving subjects. *Nat Biotechnol* **42**, 448–457 (2024).
36. Boas, D. A. & Yodh, A. G. Spatially varying dynamical properties of turbid media probed with diffusing temporal light correlation. *J. Opt. Soc. Am. A* **14**, 192 (1997).
37. Gao, X. *et al.* A photoacoustic patch for three-dimensional imaging of hemoglobin and core temperature. *Nat Commun* **13**, 7757 (2022).
38. Jin, H. *et al.* A flexible optoacoustic blood 'stethoscope' for noninvasive multiparametric cardiovascular monitoring. *Nat Commun* **14**, 4692 (2023).
39. Agrawal, S., Panchawagh, H. V. & Djordjev, K. Miniaturized Photoacoustic Wrist Sensor with Beamforming for Radial Artery Imaging and Vascular Dynamics Study. in *2025 IEEE International Ultrasonics Symposium (IUS)* 1–4 (IEEE, Utrecht, Netherlands, 2025). doi:10.1109/IUS62464.2025.11201743.
40. Panchawagh, H. V., Agrawal, S., Soukup, B. H. & Djordjev, K. Compact Photoacoustic Imaging Module for Wearable Health Monitoring Devices. in *2025 IEEE International Ultrasonics Symposium (IUS)* 1–4 (IEEE, Utrecht, Netherlands, 2025). doi:10.1109/IUS62464.2025.11201517.
41. Wang, L. V. Photoacoustic Imaging and Spectroscopy.
42. Wang, L. V. & Wu, H. *Biomedical Optics: Principles and Imaging*. (Wiley, Chicester, 2012).
43. Khan, M. I., Sun, T. & Diebold, G. J. Photoacoustic waves generated by absorption of laser radiation in optically thin cylinders. *The Journal of the Acoustical Society of America* **94**, 931–940 (1993).
44. Diebold, G. J., Sun, T. & Khan, M. I. Photoacoustic monopole radiation in one, two, and three dimensions. *Phys. Rev. Lett.* **67**, 3384–3387 (1991).

45. Marti, D., Aasbjerg, R. N., Andersen, P. E. & Hansen, A. K. MCmatlab: an open-source, user-friendly, MATLAB-integrated three-dimensional Monte Carlo light transport solver with heat diffusion and tissue damage. *J. Biomed. Opt.* **23**, 1 (2018).
46. Jacques, S. L. Optical properties of biological tissues: a review. *Phys. Med. Biol.* **58**, R37–R61 (2013).
47. Bosschaart, N., Edelman, G. J., Aalders, M. C. G., Van Leeuwen, T. G. & Faber, D. J. A literature review and novel theoretical approach on the optical properties of whole blood. *Lasers Med Sci* **29**, 453–479 (2014).
48. Van Gemert, M. J. C. *et al.* Optical properties of human blood vessel wall and plaque. *Lasers Surg Med* **5**, 235–237 (1985).
49. American National Standards Institute. American National Standard for Safe Use of Lasers (ANSI Z136.1). (2022).
50. Rock'well, R. J. RESEARCH ON HUMAN SKIN LASER DAMAGE THRESHOLDS. (1974).
51. Klein, E. *et al.* Laser Irradiation of the Skin**From the Department of Dermatology, Buffalo, NY. *Journal of Investigative Dermatology* **43**, 565–570 (1964).
52. Goldman, L., Blaney, D. J., Kindel, D. J. & Franke, E. K. Effect of the Laser Beam on the Skin**From the Departments of Dermatology and Physics of the University of Cincinnati. The study was done under a grant from the U.S. Public Health Service 0H0018. *Journal of Investigative Dermatology* **40**, 121–122 (1963).
53. Hazan, Y., Levi, A., Nagli, M. & Rosenthal, A. Silicon-photonics acoustic detector for optoacoustic micro-tomography. *Nat Commun* **13**, 1488 (2022).
54. Cubeddu, R., Pifferi, A., Taroni, P., Torricelli, A. & Valentini, G. A solid tissue phantom for photon migration studies. *Phys. Med. Biol.* **42**, 1971–1979 (1997).
55. Kuzmina, I. *et al.* Agar-based phantoms for skin diagnostic imaging. in *Tissue Optics and Photonics* (eds Zalevsky, Z., Tuchin, V. V. & Blondel, W. C.) 15 (SPIE, Online Only, France, 2020). doi:10.1117/12.2555674.
56. Souza, R. M. *et al.* Standard operating procedure to prepare agar phantoms. *J. Phys.: Conf. Ser.* **733**, 012044 (2016).
57. Jonasson, H. *et al.* Absorption and reduced scattering coefficients in epidermis and dermis from a Swedish cohort study. *J. Biomed. Opt.* **28**, (2023).
58. Ferreira, J. P. *et al.* Stability of blood gases when refrigerated. *New Zealand Journal of Medical Laboratory Science* **66**, 42–45 (2012).
59. S. V. Menon & C. S. Seelamantula. Robust Savitzky-Golay filters. in *2014 19th International Conference on Digital Signal Processing* 688–693 (2014). doi:10.1109/ICDSP.2014.6900752.
60. Or, Z., Levi, A. R., Hazan, Y. & Rosenthal, A. Hand-Held Optoacoustic System for the Localization of Mid-Depth Blood Vessels. *Photonics* **9**, 907 (2022).
61. Ilkhechi, A. K., Ceroici, C., Li, Z. & Zemp, R. Transparent capacitive micromachined ultrasonic transducer (CMUT) arrays for real-time photoacoustic applications. *Opt. Express* **28**, 13750 (2020).
62. Manwar, R., Kratkiewicz, K. & Avanaki, K. Overview of Ultrasound Detection Technologies for Photoacoustic Imaging. *Micromachines* **11**, 692 (2020).
63. Xia, W. *et al.* An optimized ultrasound detector for photoacoustic breast tomography. *Med Phys* **40**, 032901 (2013).
64. Zhang, H. *et al.* High Efficiency Silicon Nitride Grating Coupler with DBR. *Applied Physics A: Materials Science & Processing* **115**, 551 (2013).
65. Vitali, V., Lacava, C., Domínguez Bucio, T., Gardes, F. Y. & Petropoulos, P. Highly efficient dual-level grating couplers for silicon nitride photonics. *Sci Rep* **12**, 15436 (2022).
66. Van Vaerenbergh, T. *et al.* Wafer-level testing of inverse-designed and adjoint-inspired vertical grating coupler designs compatible with DUV lithography. *Opt. Express* **29**, 37021–37036 (2021).
67. Ceh, D., Peters, T. M. & Chen, E. C. S. Acoustic characterization of polyvinyl chloride and self-healing silicone as phantom materials. in (eds Hoeschen, C., Kontos, D. & Flohr, T. G.) 94123G (Orlando, Florida, United States, 2015). doi:10.1117/12.2081449.

68. Manickam, K., Machireddy, R. R. & Seshadri, S. Characterization of biomechanical properties of agar based tissue mimicking phantoms for ultrasound stiffness imaging techniques. *Journal of the Mechanical Behavior of Biomedical Materials* **35**, 132–143 (2014).
69. Zell, K., Sperl, J. I., Vogel, M. W., Niessner, R. & Haisch, C. Acoustical properties of selected tissue phantom materials for ultrasound imaging. *Phys. Med. Biol.* **52**, N475–N484 (2007).
70. Ziskin, M. C. Fundamental physics of ultrasound and its propagation in tissue. *RadioGraphics* **13**, 705–709 (1993).
