# Supplementary material for: Silicon-photonics optoacoustic sensor for fully wearable blood pressure monitoring
Source: Nat Commun. 2026 Sep 17;17:10931. doi: 10.1038/s41467-026-77580-3 (PMC13680363; doi:10.1038/s41467-026-77580-3)
Supplement: Supplementary file 3 [file 41467_2026_77580_MOESM3_ESM.pdf]

nature portfolio

Peer Review File

Silicon-photonics optoacoustic sensor for fully wearable blood pressure monitoring

Corresponding Author: Professor Amir Rosenthal

This file contains all reviewer reports in order by version, followed by all author rebuttals in order by version.

Version 0:

Reviewer comments:

Reviewer #1

(Remarks to the Author)
The authors propose a novel optoacoustic approach for noninvasive measurement of the arterial blood pressure waveform using a SPADE-based sensing system. The concept is innovative, and the technical effort is appreciated. However, several substantive concerns remain regarding validation, interpretation, and methodological rigor. Accordingly, major revision is recommended prior to consideration for publication.
1. ‘Specifically, the echoes from the artery`s walls are comparable in magnitude to signals from their vicinity, thus requiring spatial discrimination achieved either by fully imaging the artery`s morphology in 3D or using a focused acoustic beam that is properly aligned to the artery. A widely proposed solution is to use a linear array with sufficient spatial redundancy and to identify the transducer that captures the vessel diameter, showing that neither 3D imaging nor a focused beam is necessary.
2. One strength stated for the proposed work is the lowered system complexity. However, based on Supplementary Fig. 32 and Supplementary Fig. 36, the system complexity does not seem reduced: the major component still involves a wearable probe with a cm*cm level footprint and backend optical components and sampling components, which is of similar size, weight, and power as systems in the previously published literature. Moreover, each component appears necessary to ensure high-SNR signal generation and reception.
3. The real benefit of using the optoacoustic method for blood vessel diameter detection is that the broad field of diffused light can cover the full artery, while for pulse-echo ultrasound, it might be difficult. If a certain pulse-echo ultrasound transducer can transmit and receive ultrasound with a uniform directionality in the azimuth directions and strong angular selectivity in the elevational plane (which is achievable with a 1-D ultrasound array with planewave transmission and acoustic lens), it would achieve the same result as photoacoustic sensors with no need for precise alignment over the blood vessel, since the echo would be coming from the full vessel wall, not the anterior and posterior walls, similar to optoacoustic signal coming from the entire lumen of vessel. The homogeneous characteristic of the circular shape of the signal source provides the merit of loose requirement on sensor placement. One may say that in the axial direction along the vessel, the overextension of the ultrasound field would surely introduce some error due to triangulation, but that would be the same case for the proposed optoacoustic method.
4. The reported 125 MHz bandwidth of the SPADE detector is well-suited for resolving fine structural details. However, frequency components above roughly 20-30 MHz are generally expected to experience strong attenuation when propagating through several millimeters of human tissue. This attenuation could change the sharp signal features, such as inflection points or minima, that the diameter extraction algorithm depends on. The issue further deepens if the sensor is not well aligned to the target artery, which is a key application scenario of the proposed method, where the attenuation is stronger due to elongated acoustic path, leading to lowered signal fidelity. Additional discussion on how the system accounts for this depth-dependent loss would help clarify whether feature localization remains stable and free of systematic bias.
5. Identifying the distal vessel boundary by detecting a signal “minimum” may also be technically challenging in an optoacoustic context. Due to optical fluence decay, the deposited energy at the distal side of the artery is likely lower than at the proximal side, leading to a reduced signal-to-noise ratio. This could make minimum detection more sensitive to background noise or reflections from deeper structures. In a real-time implementation, such effects might occasionally cause instability or discontinuities in tracking the BP waveform.
6. The device characterization and method validation requires much better, close-to-reality design. What is the absolute amplitude of incident light after tissue penetration generated by the proposed small-footprint device? Also, the artery is located around 4 mm deep and has a diameter of 2 mm, so characterization should be done at 6 mm instead of 4 mm. What’s more, in Supplementary Fig. 15e, it appears that the device is emitting white, broadband light.

7. It is confusing how the waveform morphology would be like the ones in Fig. 2c,f, and Fig, 4, as the received pressure signal is still ultrasound and is supposed to come with positive-negative waveform, like in Ref.1 Of course, due to scatterer oscillation mode, signal filtering, and system errors, strict equality of positive and negative (expansion-compression) signal is not expected. However, in the aforementioned figures and in the left panel of Supplementary Fig. 25, where pulse-echo ultrasound is used, the signal appears unipolar.
8. The proposed method is validated against NOVA in Fig. 6. It would be recommended to validate the method against gold standard measurements, like a sphygmomanometer. Also, please show the limits of agreement for both SBP and DBP in Fig. 6i. Also, judging from Fig. 6h, there seems to be ~10 mmHg mean error for the SBP, which is considered large since the AAMI/ANSI/ISO 81060-2 requires a mean error ≤ ±5 mmHg and standard deviation ≤ 8 mmHg.
9. The authors state that 'the fundamental nature of pulse-echo ultrasound limits its applicability for fully wearable BP monitors, in which both the sensor and its electronics are miniaturized.' Fully wearable ultrasound BP monitors have been demonstrated, including in Ref.2,3

References
1 Nagli, M. et al. Silicon photonic acoustic detector (SPADE) using a silicon nitride microring resonator. Photoacoustics 32, 100527, doi:https://doi.org/10.1016/j.pacs.2023.100527 (2023).
2 Lin, M. et al. A fully integrated wearable ultrasound system to monitor deep tissues in moving subjects. Nat Biotechnol 42, 448-457, doi:10.1038/s41587-023-01800-0 (2024).
3 Yuan, J. et al. Skin-adaptive focused flexible micromachined ultrasound transducers for wearable cardiovascular health monitoring. Science Advances 11, eadw7632, doi:doi:10.1126/sciadv.adw7632 (2025).

Reviewer #2

(Remarks to the Author)
This is a very solid work where the authors demonstrated a Photoacoustic (PA) based blood-pressure sensing system based a silicon-photonics acoustic detector (SPADE) developed previously by the group. The approach is to directly measure the time variation of arterial lumen diameter in a single-element configuration to infer the blood pressure using the empirical Hayashi's model. SPADE is a silicon-nitride Bragg-cavity resonator coated with PDMS that exhibits a noise-equivalent pressure of ~2 mPa/√Hz, a wide bandwidth of ~125 MHz, and an effective sensing length of ~80 µm, yielding ~10–20 µm axial resolution and SNR ≈20 dB in vivo signal. The BP results were compared with a reference (Finapres NOVA). This work highlights its potential as a hardware foundation for future wearable blood-pressure monitoring systems. The manuscript was written with much technical details. I'd recommend acceptance after the authors address some technical questions outlined below:

1. The authors mention using grating couplers as the I/O for the acoustic chip. From the SEM image of the grating couplers in supplementary fig. 15b, the grating couplers seem to have an apodized design. What is the coupling efficiency of these couplers? Also, grating couplers need precise fiber-to-chip coupling to maintain good coupling efficiency and any small vibration can hamper the alignment. The authors mentioned that the polished fibers were coated with gold and then bonded with the grating couplers. The authors can elaborate on this bonding technique and how it insured a stable fiber-to-chip coupling. This will be beneficial to researchers working in the similar field. Also, the authors can also discuss why they chose grating couplers as I/O instead of edge couplers. The authors should also compare their coupler's performance with reported grating couplers based on Si3N4.
2. The authors use a bragg-cavity resonator to achieve a high Q-factor (2x105). What advantages does a bragg-cavity resonator have over other type of resonators, such as a microring resonator? Si3N4 microring resonators are typically reported to have very high Q-factor (106), which is one order higher than the reported value in this work, e.g. https://opg.optica.org/oe/fulltext.cfm?uri=oe-28-3-4010
3. In this work, the authors employ a single Bragg-cavity resonator as the ultrasound detector. Could the authors comment on the potential advantages or trade-offs of using an array of such resonators in this application?
4. In fig. 3d, one can observe two illumination spots due to the splitting of the input fiber bundle. Is the splitting to provide wider range of illumination to the artery?
5. In the in vivo experiments, the artery is manually pre-localized by palpation and the probe is then positioned and fine-adjusted by searching for the minimum signal delay. Could authors clarify how much operator intervention or search time is required and whether an automated initialization or self-localization procedure is feasible?
6. Please discuss limitation of the current system in realistic motion conditions such as wrist movement, or additional disturbances that expected in daily wearable use. E.g. these could strongly affect both optical coupling and mechanical loading. Can the authors extend the experimental evaluation to include such dynamic motion scenarios, or explicitly discuss the expected limitations?
7. The authors give a detailed explanation of their calibration setup in the BP monitoring and validation section. Is the calibration procedure subject-dependent? For example, will the same calibration procedure withhold for a new test subject? If not, could this model be made calibration-free in the future? What are the challenges for that?
8. Finally, although the sensing concept is compelling, the current implementation relies on bulky laboratory hardware (tunable laser, fiber interferometer, and bench-top electronics), and the gap to a truly wearable system remains large. The authors can discuss about the challenges to develop a fully wearable portable system and possible strategies to address these challenges.

Reviewer #3

(Remarks to the Author)
I co-reviewed this manuscript with one of the reviewers who provided the listed reports. This is part of the Nature Communications initiative to facilitate training in peer review and to provide appropriate recognition for Early Career Researchers who co-review manuscripts.

Version 1:

Reviewer comments:

Reviewer #1

(Remarks to the Author)
The revised manuscript is substantially improved, and the authors have made strong efforts to address the technical concerns raised in the previous review, which are greatly appreciated. It is recommended that the remaining concerns be addressed before this manuscript sees publication.
1. The bandwidth concern is now largely resolved. Supplementary Figs. 40–42 directly address the concern that tissue attenuation would reduce the effective bandwidth to well below the reported 125 MHz of the SPADE detector. The authors appropriately acknowledge that the intrinsic 125 MHz detector bandwidth is not the effective in vivo bandwidth at the radial-artery depth, and Supplementary Fig. 40 is helpful in showing that the diameter-estimation error remains small above approximately 6 MHz. The manuscript should nevertheless consistently refer to 125 MHz as the intrinsic detector bandwidth, rather than implying that this is the effective in vivo bandwidth. Also, this necessarily raises the question of whether the high bandwidth of 125 MHz is necessary, as shown in Supplementary Fig. 40, a much smaller bandwidth is sufficient.
2. The added analysis of the leading/trailing discontinuity ratio and the discussion of dark-field illumination provide a plausible explanation for why the posterior boundary remains detectable. However, because the method is intended for continuous monitoring, the authors should state explicitly whether any beats or measurements failed automatic boundary tracking, whether any events were rejected by the processing algorithm, and whether any manual correction was applied. This information would directly address the concern that minimum-based tracking may occasionally become unstable in real-time or long-term operation. The automated processing criteria should also be sufficiently clear to enable readers to understand how the reported BP accuracy metrics were obtained.
3. The optical-fluence characterization is improved as well. These additions address the concern that the posterior side of the artery may be insufficiently illuminated. The reviewer recommends that the authors, if possible, add a more explicit quantitative statement of the fluence, or the anterior/posterior signal ratio, at the relevant anterior and posterior arterial depths. This would make the response to the 4–6 mm depth issue clearer without requiring readers to infer values from the figure.
4. The clinical-validation language should be tempered. The additional NOVA validation against a brachial cuff is useful, and the revised Bland-Altman plot now appropriately separates SBP and DBP, and the apparent concern about a large SBP mean bias has also been resolved. However, the study still includes only two subjects, uses subject-specific calibration, and compares the optoacoustic estimates with a NOVA-derived radial reference waveform rather than with an invasive radial arterial line or a full sphygmomanometer-based validation protocol. The data support a pilot feasibility comparison, not full clinical validation. The statements “within clinically acceptable standards” and “corresponds to a grade A device” should be explicitly qualified or removed. A more accurate formulation would be that the two-subject pilot study achieved low MAD relative to a calibrated NOVA-derived radial reference, while full compliance with cuffless BP validation standards remains to be demonstrated in a larger clinical study. The manuscript should also avoid implying that the 120,000 sampled diameter-pressure pairs are statistically independent validation samples. The primary performance metrics appear to be based on 120 ten-second measurements, and this should be stated consistently.
5. The discussion of prior wearable ultrasound systems should also be more balanced. The authors now appropriately acknowledge that wearable ultrasound BP monitors and fully integrated wearable ultrasound systems have been reported. However, the revised manuscript should avoid implying that pulse-echo ultrasound is fundamentally incompatible with wearable BP monitoring. The authors’ data support a narrower and more defensible claim: current pulse-echo implementations face a trade-off between placement robustness and system complexity, particularly for small superficial arteries such as the radial artery near the wrist.

Reviewer #2

(Remarks to the Author)
The answers and revisions by the authors are satisfactory; acceptance is recommended.

Reviewer #3

(Remarks to the Author)
I co-reviewed this manuscript with one of the reviewers who provided the listed reports. This is part of the Nature Communications initiative to facilitate training in peer review and to provide appropriate recognition for Early Career Researchers who co-review manuscripts.

Version 2:

Reviewer comments:

Reviewer #1

(Remarks to the Author)
The authors have properly addressed the previous comments, and I have no further concerns. My suggestion is Accept.

Open Access This Peer Review File is licensed under a Creative Commons Attribution 4.0 International License, which permits use, sharing, adaptation, distribution and reproduction in any medium or format, as long as you give appropriate credit to the original author(s) and the source, provide a link to the Creative Commons license, and indicate if changes were made.
In cases where reviewers are anonymous, credit should be given to 'Anonymous Referee' and the source.
The images or other third party material in this Peer Review File are included in the article’s Creative Commons license, unless indicated otherwise in a credit line to the material. If material is not included in the article’s Creative Commons license and your intended use is not permitted by statutory regulation or exceeds the permitted use, you will need to obtain permission directly from the copyright holder.
To view a copy of this license, visit https://creativecommons.org/licenses/by/4.0/

Reviewer 1

The authors propose a novel optoacoustic approach for noninvasive measurement of the arterial blood pressure waveform using a SPADE-based sensing system. The concept is innovative, and the technical effort is appreciated. However, several substantive concerns remain regarding validation, interpretation, and methodological rigor. Accordingly, major revision is recommended prior to consideration for publication.

We appreciate the Reviewer's constructive feedback. The manuscript was modified according to the Reviewer's comments. In the following is a point-by-point response addressing each remark.

1-1

Specifically, the echoes from the artery`s walls are comparable in magnitude to signals from their vicinity, thus requiring spatial discrimination achieved either by fully imaging the artery`s morphology in 3D or using a focused acoustic beam that is properly aligned to the artery. A widely proposed solution is to use a linear array with sufficient spatial redundancy and to identify the transducer that captures the vessel diameter, showing that neither 3D imaging nor a focused beam is necessary.

Indeed, the most practical wearable ultrasound system today is based on a dense 1D array, as recently developed by the group of S. Xu in Ref.[1], which represents a good tradeoff between full 3D systems and focused single-element transducers. However, as now emphasized in the Introduction and supported with additional measurements (Supplementary Note 5 and Supplementary Fig. 47), the success of 1D arrays is a result of the focusing (or collimation) properties of their individual elements. The ability to choose the optimal element is effectively an alignment procedure that is performed electronically, instead of the mechanical procedure required for single-element transducers, but the elements themselves generate semi-collimated beams. Despite their success, 1D arrays still suffer from two disadvantages compared to an ideal 3D system:

- They require that the transducer be positioned parallel to the artery. As experimentally shown in Figs. 5i-l, even a yaw angle of 10° can lead to variations in the diameter estimation that go well beyond the variations expected in a resting position (see comparison to the results obtained by our system in Figs. 5a-d). This angle sensitivity requires careful positioning of the ultrasound patch, which might be reasonable in a hospital setting, but challenging in devices intended for home use.
- Even with dense arrays, a slight shift, smaller than the array pitch, in the position of the transducer can still lead to large errors in the diameter estimation. For example, we show in Supplementary Fig. 8 that a shift of 0.3 mm leads to a typical estimation error of 150 µm in a measurement performed in the radial artery close to the wrist. We note that the most advanced wearable 1D array for BP monitoring from the group of S. Xu[1] had an element pitch of 0.5 mm, which could lead to comparable errors if applied close to the wrist. Interestingly, the work in Ref.[1] used the ultrasound patch in the region of the elbow, where the artery is larger (brachial), thus reducing the positioning sensitivity.

In our reply to Comment 1-3, we show that when using planewaves, which lack focus in the lateral direction, echoes from the sides of the artery become significant, thus demonstrating the importance of focusing in pulse-echo monitoring of arterial diameter.

1-2

One strength stated for the proposed work is the lowered system complexity. However, based on Supplementary Fig. 32 and Supplementary Fig. 36, the system complexity does not seem reduced: the major component still involves a wearable probe with a cm*cm level footprint and backend optical components and sampling components, which are of similar size, weight, and power as systems in the previously published literature. Moreover, each component appears necessary to ensure high-SNR signal generation and reception.

Indeed, the current prototype system is still bulky and based on expensive components, as is often the case with first-generation systems. However, it is not true that these types of components are essential to ensure high SNR in all configurations. Rather, these components simplified the engineering effort required to develop the first prototype, enabling us to focus on the fundamentals. We now discuss in more detail in the Discussion how the system's complexity could be massively reduced, potentially to the level of a wearable device, while maintaining sufficient SNR. Importantly, our proposed strategy for reducing complexity relies on components and capabilities which have already been developed in previous works, and thus it may be achieved by pure engineering effort. In the following, we briefly discuss the main components in our system and how they could be replaced:

- Optoacoustic laser: in our setup we used a preexisting InnoLas OPO source that can deliver up to 30 mJ pulses with durations of 7 ns at arbitrary wavelengths in the range of 680 - 980 nm. In all three parameters, the performance of this laser was superfluous, but it did simply the system development considering the overall uncertainty in overall performance. In practice, we used pulses of only 10 mJ (4.9 mJ at the skin) and a single wavelength of 800 nm, whereas the pulse duration could have been increased to 25 ns without affecting the bandwidth of the signal (see our reply to Comment 1-4). Additionally, our analysis shows that the intrinsic noise of the sensor was approximately half of the background signal from the tissue (Methods), and therefore similar results could have been obtained with lower pulse energies, *e.g.,* 3 mJ. Such energy can be readily achieved with pulsed laser diodes, which are commercially available (e.g., Lumibird). Furthermore, since such laser diodes can operate at a much higher repetition rate than the OPO source used in this work, lower energies may be used, enabling cheaper sources, with signal averaging used to partially compensate for the loss of signal. An additional option is VCSELs, which has been demonstrated both in optoacoustic patches[2] and handheld devices[3]. Readout system: The interferometric system currently consists of a fiber-based Mach-Zehnder interferometer, photodiodes, and a tunable CW laser. All these components can be miniaturized today on a single silicon-photonics chip without sacrificing performance. Typical examples may be found in Ref [4].
- Sampling electronics: since we need to sample only a single channel and do not require to generate a voltage pulse, the electronics are considerably simplified. For example, typical chips for analog to digital conversion from TI (e.g. ADC3563) have typical dimensions of 5 × 5 mm and cost less than 10$.
- Wearable probe: Indeed, the wearable probe in our work had dimensions with cm × cm footprint to enable ±1 cm positioning robustness. While ultrasound patches, based on dense arrays, would require the same footprint, there is a major difference. In the case of ultrasound patches, the entire patch needs to be in contact with the skin to enable all the individual elements to operate. In our case, only a single element, which can be sub-millimeter in width, needs to be in contact with the tissue, whereas the illumination can be applied without any contact, facilitating simpler designs. For example, the illumination may be applied by a wearable, loose contact, wrist band or watch.
- System simplification via increased sensitivity: We now discuss that further improvement to the sensitivity of SPADE may be achieved, enabling further reduction in the optoacoustic illumination power, and thus simpler miniaturization of the source. Specifically, since our main noise source is photon shot noise (Supplementary Fig. 13), which is independent of the resonator's Q-factor, further improvement in sensitivity can be achieved by using higher Q-factors, which will lead to stronger signals. Indeed, as mentioned by Reviewer 2, Q-factors of 10^6, 5 times higher than ours, are common in silicon photonics.

1-3

The real benefit of using the optoacoustic method for blood vessel diameter detection is that the broad field of diffused light can cover the full artery, while for pulse-echo ultrasound, it might be difficult. If a certain pulse-echo ultrasound transducer can transmit and receive ultrasound with a uniform directionality in the azimuth directions and strong angular selectivity in the elevational plane (which is achievable with a 1-D ultrasound array with planewave transmission and acoustic lens), it would achieve the same result as photoacoustic sensors with no need for precise alignment over the blood vessel, since the echo would be coming from the full vessel wall, not the anterior and posterior walls, similar to optoacoustic signal coming from the entire lumen of vessel. The homogeneous characteristic of the circular shape of the signal source provides the merit of loose requirement on sensor placement. One may say that in the axial direction along the vessel, the overextension of the ultrasound field would surely introduce some error due to triangulation, but that would be the same case for the proposed optoacoustic method.

The Reviewer suggests a new, alternative pulse-echo approach to measure the diameter of arteries, which uses plane waves to emulate the geometrical underpinnings of the optoacoustic method. To the best of our knowledge, this is an original idea that has not been previously studied, and we therefore performed our own study, which is described in the next paragraph. Our results (added to the now expanded Supplementary Note 5) show that using planewaves, although elegant and conceptually compelling in its affinity to the optoacoustic approach, produces borderline-quality signals, which are worse than those produced by 1D array ultrasound patches. This lower performance may explain why planewaves were never adopted in previous ultrasound studies of S. Xu`s group, despite them having all the relevant hardware available. Our study was realized using a 1D array, as suggested, and included two options for the reception (Rx): single-element detector and planar detector. The previously described Telemed platform was used for both options, with special configurations for each.

- Planewave Tx / single-element Rx**:** Our simulations show that if the detector is point like, *i.e.,* lacks any focusing, the delays from the artery walls depend on its angle with respect to the plane wave. The reason for this effect is that the delay is determined by the total acoustic path of the incident wave and scattered wave, whose length depends on the angle between these two waves. Thus, the delays cannot be directly translated to distances because this relative angle and the true paths are unknown. This result is well known in scattering theory in the frequency domain, where the phase of the scattered waves depends on the angle[5]. The most extreme case of this effect, although not relevant to our work, is when the detector is on the other side of the artery. In that case, the scattered waves from the back and front of the artery, as well as the incident wave, reach the detector at the same time since they all include the same total travelled distance. *In vivo* measurements of this approach agree with the theory and with the simulations, and are all shown in the newly added Supplementary Fig. 46.
- Both Tx and Rx using planewaves**:** If the detector also corresponds to a planewave, *i.e.,* it is identical to the ultrasound transmitter as suggested by the Reviewer, then the delays of the echoes from the front and back of the artery can be used to readily calculated the artery diameter (up to the contribution of the artery wall) using the time-of-flight principle, as is depicted in the newly added Supplementary Fig. 47. This case could emulate the optoacoustic configuration in an ideal scenario in which the artery is the only source of echo signals. However, when planewaves are used, also the surrounding tissue contributes to the echo signals. As we experimentally show in Supplementary Fig. 47d, the larger the planewave, the more background signal is acquired, where already at 9.6 mm width it is challenging to discern the signals from the artery's wall from the background. When the full span of our transducer, 19.2 mm, was used, which is comparable to the 2 cm coverage of the optoacoustic probe, the echoes from the artery wall and surrounding tissue were effectively equal. Thus, the difference between pulse-echo ultrasound and Optoacoustics is not merely geometrical, but it is rather inherently physical and anatomical – Optoacoustics has an advantage in sensing and imaging of blood vessels. Namely, since the optoacoustic sources are generated only where light is absorbed by blood, the signal from isolated blood vessels dominate the measurement. In a pulse-echo measurement, the signals

from the artery do not dominate the measurement in the same way, thus requiring a sort of focusing to reject undesired background echoes (see our reply to Comment 1-1).

- Additional practical issues**:** While we show that using planewaves in pulse-echo ultrasound does not produce results comparable to Optoacoustics, it is also important to acknowledge the practical limitations that such an approach would impose:
  1) The entire ultrasound transducer would need to be in contact with the tissue, whereas in our work, only a miniature element requires contact.
  2) Conformal patches would not work properly in this approach since they would be curved and therefore not generate a planewave. Thus, a large flat transducer would be needed, making the device less wearable.
  3) If one chooses the option of planewave Tx with single-element Rx, one will still need an array from which to choose the best element. This would lead to the same complexity as the original pulse-echo patches in which the element facing the artery is used for Tx/Rx.
  4) A planewave focused in the elevational direction using an acoustic lens (as suggested by the Reviewer) effectively sees a cross section of the cylindrical artery. If the artery is orthogonal to the propagation direction (zero pitch angle), this cross section would be a circle. However, if pitch angle is nonzero, the cross section is a cylinder which is elongated in the propagation direction. Accordingly, in the presence of a pitch angle the acoustic delays will produce an estimate that is larger than the true diameter.

1-4

The reported 125 MHz bandwidth of the SPADE detector is well-suited for resolving fine structural details. However, frequency components above roughly 20-30 MHz are generally expected to experience strong attenuation when propagating through several millimeters of human tissue. This attenuation could change the sharp signal features, such as inflection points or minima, that the diameter extraction algorithm depends on. The issue further deepens if the sensor is not well aligned to the target artery, which is a key application scenario of the proposed method, where the attenuation is stronger due to elongated acoustic path, leading to lowered signal fidelity. Additional discussion on how the system accounts for this depth-dependent loss would help clarify whether feature localization remains stable and free of systematic bias.

We thank the Reviewer for this insightful comment, which was not sufficiently address in the original manuscript. Indeed, while the extreme bandwidth of SPADE could be useful for very superficial blood vessels, acoustic attenuation limits the usable bandwidth to below 20 MHz at the depths relevant to our work. To address the Reviewer's comment, we quantified the bandwidth reduction due to acoustic attenuation and analyzed its effect on the signal fidelity. Our analysis shows that although acoustic attenuation reduces the bandwidth, its effect on the diameter estimation is negligible, even under the uncertainty in the position of the detector. In addition, even in the presence of acoustic attenuation, the bandwidth of the signals detected in our system still exceed those of previous wearable system. Thus, our system still possesses a bandwidth advantage for wearable BP monitoring enabled by SPADE, albeit much more modest than SPADE's intrinsic capabilities. The manuscript was modified to address this crucial point (Discussion and Supplementary Materials), as summarized in the following:

- The BW estimations were performed using three different approaches. In the first approach, the signal's bandwidth was estimated from the phantom measurement described in Supplementary Note 11. As shown in Supplementary Table 3, the acoustic attenuation of our phantom resembles those of tissue. In the second approach, numerical simulations were conducted with k-wave using typical attenuation values from the literature. The results of the two approaches are shown in shown in Supplementary Fig. 41a, where the bandwidth is presented as a function of the offset. As shown in the figure, the phantom measurement and numerical simulation are in general agreement, respectively revealing bandwidth limits of 18 and 17 MHz when the detector is above the artery, and 8 and 6 MHz when the detector is at an offset of 1 cm from the artery. In the third approach, we used the *in vivo* data to assess the signal's bandwidth. Here, a distribution of the BW values was generated for 114,000 events from 90 random measurements, showing that the estimated bandwidth had a median value of 11.68 MHz, and was in the range of 5 - 28 MHz for 95% of the measurements, with only 7% of the measurements under 6 Mhz. The technique used to estimate the bandwidth from the acoustic signals is described in Supplementary Fig. 42b and Supplementary Table 5.
- The effect of the limited BW on the estimation accuracy of the artery diameter is now studied in more detail in Supplementary Fig. 40. The results show that for bandwidths above 6 MHz, the absolute error in the diameter estimation is lower than 3 µm, which is an insignificant value, corresponding to less than 1 mmHg, as shown in Supplementary Fig. 18b.
- We summarize the performance achieved by different systems Supplementary Fig. 42, showing that even in the presence of acoustic attenuation, our system effectively achieves considerably higher bandwidths than those achieved by piezoelectric-based ultrasound patches. Furthermore, the figure shows that if the OPO source used in the current work for optoacoustic excitation were to be replaced by laser diodes with a pulse FWHM of 20 ns, an intrinsic BW of 21 MHz would be achieved, which is higher than the limitation imposed by acoustic attenuation. Thus, our BW analysis strengthens our response to Comment 1-2 that the OPO source may be replaced by cheaper diode lasers without affecting the signal fidelity.

1-5

Identifying the distal vessel boundary by detecting a signal “minimum” may also be technically challenging in an optoacoustic context. Due to optical fluence decay, the deposited energy at the distal side of the artery is likely lower than at the proximal side, leading to a reduced signal-to-noise ratio. This could make minimum detection more sensitive to background noise or reflections from deeper structures. In a real-time implementation, such effects might occasionally cause instability or discontinuities in tracking the BP waveform.

Indeed, the decay in optical fluence can lead, in some cases, to a weaker signal from the artery’s posterior side, and we agree with the Reviewer that searching for the minimum in an optoacoustic signal may, in the general case, be prone to instabilities. To reduce this effect, we used broad, dark-field illumination and deep-penetration NIR illumination for which light scatters around the artery to reach its posterior end (originally discussed in Supplementary Note 8). Since the depths are relatively superficial, the difference between the leading and trailing discontinuities is acceptable when dark-field illumination is used. In our *in vivo* study we found that the ratio between the signal’s leading discontinuity and trailing discontinuity was typically in the 0.55 – 1.24 range (FWHM), as shown in the newly added Supplementary Fig. 23a. The trailing discontinuity often surpasses the leading one, as exemplified in Fig. 5d. We note that signals and reflections from deeper tissue arrive later in time than the signal of the posterior point and explain that the effect of these signals is eliminated by applying a window to the signal (Methods). In practice, after applying a window, the signal’s minimum proved as a very robust identifier of the artery’s posterior.

To further study how the amplitude of the signal’s discontinuities changes with the illumination, we performed a numerical study in the newly added Supplementary Fig. 23b. The numerical results show that the ratio between the signal’s maximum and minimum does not change considerably as a function of the optoacoustic sensor’s offset, generally supporting robust results obtained in the experiments. We now highlight in the discussion that the relatively comparable magnitude of the leading the trailing discontinuities were a robust feature of our *in vivo* study, which is now also supported by the numerical simulations. Interestingly, while the numerical simulations predicted that the anterior wall will always produce a stronger signal than the posterior wall, in some measurements the opposite was true. A possible explanation for this effect is that the radial bone, which was not simulated, and is approximately 4 mm below the artery acts as a reflector that contributes to the illumination of the posterior wall.

1-6

The device characterization and method validation requires much better, close-to-reality design. What is the absolute amplitude of incident light after tissue penetration generated by the proposed small-footprint device? Also, the artery is located around 4 mm deep and has a diameter of 2 mm, so characterization should be done at 6 mm instead of 4 mm. What's more, in Supplementary Fig. 15e, it appears that the device is emitting white, broadband light.

We have added additional simulation results in Supplementary Fig. 12 depicting both the illumination pattern and fluence at different penetration depths and showing good correlation to the 4 mm photograph in Fig. 3b. The original goal of the characterization in Fig. 3b at a typical depth was merely to show that the dark region in the illumination at the probe's output does not remain dark after traveling 4 mm and reaching the artery, but it is still weaker than in sides, which is desirable in order to reduce the magnitude of the leading discontinuity to the level of the trailing discontinuity (see our response to Comment 1-5).

Regarding the posterior side of the artery and the illumination that reaches it, we added the numerical analysis of Supplementary Fig. 23, also discussed in our response to Comment 1-5, showing that the illumination is comparable to that of the anterior side (up to a factor of 2) owing to the dark-field illumination.

Regarding Supplementary Fig. 26e (originally named 15e), the photo was taken with white light merely for illustration purposes. The true wavelength of 800 nm is essentially undetectable by the human eye. While the digital camera could still "see" this wavelength, we preferred not to give the false impression that the illumination is visible. We replaced the photo with a new one that matches the other ones in Supplementary Fig. 26, in which the OPO was temporarily set to 680 nm so that a (conventional) camera can capture it.

1-7

It is confusing how the waveform morphology would be like the ones in Fig. 2c,f, and Fig, 4, as the received pressure signal is still ultrasound and is supposed to come with positive-negative waveform, like in Ref.[6] Of course, due to scatterer oscillation mode, signal filtering, and system errors, strict equality of positive and negative (expansion-compression) signal is not expected. However, in the afore mentioned figures and in the left panel of Supplementary Fig. 25, where pulse-echo ultrasound is used, the signal appears unipolar.

The optoacoustic waveforms shown in Figs. 2c, 2f and 4, while may be confusing in the context of pulse-echo ultrasound, fit very well the predictions of optoacoustic theory. While in pulse-echo ultrasound the transmitted and back-scattered waves have a carrier frequency and thus rapidly oscillate between positive and negative values (which we believe this is what the Reviewer meant by "positive-negative waveform"), the optoacoustic signals are inherently wideband. The signals that appear in Fig. 2c match the analytical solution to the wave equation in cylindrical coordinates found over 2 decades ago, which also appears in review literature[7,8] (as originally shown in Supplementary Note 7 and Supplementary Fig. 4). Indeed, when optoacoustic signals are detected by narrowband transducers that lack the low frequencies, they resemble pulse-echo signals, but the inherent nature of optoacoustic signals is not oscillatory, but rather bipolar. In the left panel of Supplementary Figs. 25a-d, the signals were measured in an optoacoustic, not pulse-echo, configuration. The piezoelectric transducer used in this optoacoustic measurement was connected to a DAQ with a high electric input impedance, specifically designed for optoacoustics, leading to high sensitivity also for the lower frequencies, enabling us to capture the bipolar (non-oscillatory) nature of the wideband optoacoustic signals. The pulse-echo signals are shown on the right panel of Supplementary Figs. 25e-h, and they were indeed oscillatory.

1-8

The proposed method is validated against NOVA in Fig. 6. It would be recommended to validate the method against gold standard measurements, like a sphygmomanometer. Also, please show the limits of agreement for both SBP and DBP in Fig. 6i. Also, judging from Fig. 6h, there seems to be ~10 mmHg mean error for the SBP, which is considered large since the AAMI/ANSI/ISO 81060-2 requires a mean error ≤ ±5 mmHg and standard deviation ≤ 8 mmHg.

The Finapres NOVA was certified according to the ISO 81060-2 standard[9] (note that this standard is for automated sphygmomanometers, not for cuffless devices[11]) and it included a brachial cuff for calibration. As we have described, the brachial cuff was used consistently in each session. Alternatively, using only a simple brachial cuff, while indeed the recommended approach by the IEEE standard, is more difficult and prone to errors. To address the Reviewer's concerns, we have taken upon ourselves to re-validate the NOVA accuracy against the brachial cuff, as depicted in the newly added Supplementary Fig. 32. As now emphasized in the manuscript, the NOVA system has been used as a reference in previous works published in leading journals, *e.g.,* Ref.[10].

Regarding Fig. 6i - the limits of agreement have been separated as suggested and we thank the Reviewer for bringing this to our attention. Fig. 6h does not indicate a mean error of ~10 mmHg, the mean error is under 1 mmHg.

1-9

The authors state that 'the fundamental nature of pulse-echo ultrasound limits its applicability for fully wearable BP monitors, in which both the sensor and its electronics are miniaturized.' Fully wearable ultrasound BP monitors have been demonstrated, including in Ref.[12,13]

Indeed, our phrasing was not precise as we have not defined what "fully wearable" means, and we now clarify this point in the Introduction. We now explain that fully wearable, in this context means that the monitor's operation is fully robust to placement (position and angle) errors and all the electronics are wearable. We argue that the pulse-echo approach with 1D arrays currently does not fulfill either of these two criteria since it is sensitive to the relative angle with respect to the artery and has yet demonstrated wearable electronics. Our optoacoustic approach fulfills the first condition, as demonstrated both in the phantom and *in vivo* studies. While we still do not fulfill the second condition yet, as correctly pointed out by the Reviewer in Comment 1-2, we do offer a clear vision for complete wearability, based on technologies already demonstrated in the literature.

Reviewer 2

This is a very solid work where the authors demonstrated a Photoacoustic (PA) based blood-pressure sensing system based on a silicon-photonics acoustic detector (SPADE) developed previously by the group. The approach is to directly measure the time variation of arterial lumen diameter in a single-element configuration to infer the blood pressure using the empirical Hayashi's model. SPADE is a silicon-nitride Bragg-cavity resonator coated with PDMS that exhibits a noise-equivalent pressure of ~2 mPa/√Hz, a wide bandwidth of ~125 MHz, and an effective sensing length of ~80 µm, yielding ~10–20 µm axial resolution and SNR ≈20 dB in vivo signal. The BP results were compared with a reference (Finapres NOVA). This work highlights its potential as a hardware foundation for future wearable blood-pressure monitoring systems. The manuscript was written with much technical details. I'd recommend acceptance after the authors address some technical questions outlined below.

We appreciate the Reviewer's constructive feedback. The manuscript was modified according to the Reviewer's comments. In the following is a point-by-point response addressing each remark.

2-1

The authors mention using grating couplers as the I/O for the acoustic chip. From the SEM image of the grating couplers in Supplementary Fig. 15b, the grating couplers seem to have an apodized design. What is the coupling efficiency of these couplers? Also, grating couplers need precise fiber-to-chip coupling to maintain good coupling efficiency and any small vibration can hamper the alignment. The authors mentioned that the polished fibers were coated with gold and then bonded with the grating couplers. The authors can elaborate on this bonding technique and how it insured a stable fiber-to-chip coupling. This will be beneficial to researchers working in the similar field. Also, the authors can also discuss why they chose grating couplers as I/O instead of edge couplers. The authors should also compare their coupler's performance with reported grating couplers based on Si3N4.

The Reviewer is correct that fiber coupling is a significant challenge when the SiPh chip is integrated into a wearable device. Indeed, in most applications, the fiber-coupled chips are isolated from any external forces. In our work, we have devoted significant efforts to make the coupling sufficiently robust to endure the applied force applied when the device is pressed against the tissue. In the revised version of the manuscript, we further discuss this issue, highlighting this challenge and our solution. The main reason we chose grating couplers is the simplicity of the assembly, ability to easily inspect all the devices on the chip without having to cut it, and the advantage of having the fiber directly bonded to silicon chip, which is more rigid than the glass substrate, thus further contributing to the robustness of the device.

As described (Fig. 3c), the couplers had an insertion loss of 6 dB per fiber, in agreement with our previous work[6]. Our grating couplers were produced in a single etching step with a feature size of 300 nm and without any additional structures such as bottom mirrors to enhance coupling. In the newly added Supplementary Note 15 we briefly discuss more efficient grating couplers, which rely on more advanced fabrication approaches.

We now discuss our robust bonding technique in more detail in the Methods, under "SPADE assembly". We also fully describe the gear used and the rational for the material chosen. In addition, Supplementary Fig. 27 was expanded with more detail.

2-2

The authors use a bragg-cavity resonator to achieve a high Q-factor (2x105). What advantages does a bragg-cavity resonator have over other type of resonators, such as a microring resonator? Si3N4 microring resonators are typically reported to have very high Q-factor (106), which is one order higher than the reported value in this work, e.g. [14].

The main advantage of a Bragg cavity resonator is its better mode localization, leading to a smaller detector. The width of our detector is approximately equal to the width of the waveguide, *i.e.,* 1 µm, which is almost two orders of magnitude smaller than the typical acoustic wavelength in the system. As a result, the acoustic signals could be detected with no distortions even at large offsets. The length of our detector was approximately 83 µm, which is comparable to the acoustic wavelength (see in the Discussion our assessment for the dominant acoustic frequencies in the measurement), making our sensor robust against tilting. With these dimensions, our sensors may be regarded as point like. In contrast, high-Q micro-rings in silicon nitride typically have diameters of hundreds of microns, leading to a forward-looking detector that could not properly detect signals from blood vessels that are at an offset. In the reference suggested by the Reviewer, which we now cite, the micro-ring had a diameter of 600 µm.

2-3

In this work, the authors employ a single Bragg-cavity resonator as the ultrasound detector. Could the authors Comment on the potential advantages or trade-offs of using an array of such resonators in this application?

The tradeoffs in using an array of resonators are now discussed in the concluding paragraphs. The most obvious disadvantage of using arrays of resonators is the increased system complexity and challenges in miniaturizing the interferometric readout system and sampling electronics, which would need to be scaled up. Nonetheless, the increased complexity might be acceptable for a small number of resonators, which could deliver significant benefits. For example, if the elements are positioned far apart, they may enable an even easier positioning, guaranteeing that at least one detector is in the vicinity of the artery. The possibility of operating an array of detectors in series, rather than parallel, is a possible approach to achieve an acceptable increase in complexity. Specifically, the resonators may be wavelength encoded, enabling their fabrication on a single bus waveguide and sharing the optical components used in the interferometric system.

2-4

In Fig. 3d, one can observe two illumination spots due to the splitting of the input fiber bundle. Is the splitting to provide wider range of illumination to the artery?

The main goal of splitting the fiber bundle is to generate a dark-field illumination - a technique commonly used in Optoacoustics to enhance the relative signal from deep-seated targets (see Refs.[15] in the revised manuscript). This point is now analyzed in the newly added Supplementary Fig. 23. When conventional widefield illumination is used, *i.e.,* without a gap, and is centered on the artery, much of the light is blocked by the artery, leading to a weaker signal from its posterior end. As a result, the behavior of the Optoacoustics signal for such illumination would be highly dependent on positioning.

2-5

In the in vivo experiments, the artery is manually pre-localized by palpation and the probe is then positioned and fine-adjusted by searching for the minimum signal delay. Could authors clarify how much operator intervention or search time is required and whether an automated initialization or self-localization procedure is feasible?

As we now explain in the revised text, after the initial palpation is performed to find the general location of the radial artery, positioning the probe to get a usable pulsating signal takes only several seconds for an experienced operator. The fast and simple positioning is a result of the robustness of the probe to rotation and translation. We note that it is not essential to adjust the probe to reach minimum signal delay (that part was mentioned in the narrower context of Fig. 4 and was not enforced in the clinical study itself), which simplifies the positioning procedure. For example, in Fig. 4e it is shown that a proper signal is maintained even when the probe was moved to a position with a larger delay. As now mentioned in the Discussion, proper positioning depends on a single factor - does the signature pulsatile signal appear in the live M-mode data (observed on an oscilloscope). Since only a single parameter determines if the positioning is appropriate, simple computer-generated feedback can be used to guide inexperienced users on how to place the probe.

2-6

Please discuss limitations of the current system in realistic motion conditions such as wrist movement, or additional disturbances that expected in daily wearable use. E.g. these could strongly affect both optical coupling and mechanical loading. Can the authors extend the experimental evaluation to include such dynamic motion scenarios, or explicitly discuss the expected limitations?

The topic of motion was mentioned briefly in Supplementary Note 1 and Supplementary Table 1. Additional addressment is now added to the Discussion, stating that our focus was on measuring BP in a motionless seated position, and that our validation is limited to that. We have not performed testing in non-standard positions, nor in motion-involving positions. While our system could be compatible with such conditions as it is now discussed, we have decided to focus on BP monitoring in the resting position. Below is an extended explanation.

Why our system is technically compatible with realistic motion conditions

- As thoroughly validated in the text, our BP sensor does not require accurate placement. Thus, if the sensor is slightly moved because of the user motion, no effect is expected on BP accuracy.
- Moreover, as shown in Fig. 4e, even during the relative motion of the BP sensor on the skin, it kept accurately monitoring the artery diameter.
- While the amplitude of the optoacoustic signal may fluctuate due to changes in optical or acoustic coupling, it should not affect the accuracy of our diameter estimations, which depend on the delays in the optoacoustic signal.

Reasons for focusing on measurements in the resting position only

- Full compliance with standards in this field is achievable without testing for non-standard positions and without motion tests. Given that this milestone has yet to be fully achieved by wearable cuffless device, we feel that expanding beyond it at this stage is premature.
- Standards in this field primarily call for the sitting position to be evaluated, as it is the most clinically meaningful position and it is aligned with the American hearts association's guidelines for performing blood pressure measurements. The standards state that if other positions (supine, standing) are tested, each one should be evaluated fully and separately. Note that readings from each of those positions are different and bear different, more specialized, clinical meanings.
- Standards either outright discourage motion testing or simply ignore it. Their reasoning is that the reference devices themselves are incompatible with motion:
    1. Automated sphygmomanometers would withhold readings.
    2. Motion makes manual sphygmomanometry difficult, unreliable, and it extends beyond its clinical guidelines.
    3. The exitance of an A-line either call for minimal motion (if it was applied to a healthy volunteer purely for testing, which by itself is strongly discouraged by the standards and is misaligned with the good practices of clinical studies), or it serve as an indication that the subject is very ill and could not move anyway.
- Leading works in the field[1] also, understandably, avoided testing in motion; they had used motion merely to induce BP changes and had measure afterwards in static conditions.

2-7

The authors give a detailed explanation of their calibration setup in the BP monitoring and validation section. Is the calibration procedure subject-dependent? For example, will the same calibration procedure withhold for a new test subject? If not, could this model be made calibration-free in the future? What are the challenges for that?

We thank the Reviewer for raising this important point. The calibration procedure is indeed subject-specific, a detail we now explicitly clarify in the revised manuscript.

Transitioning our specific model to a calibration-free framework presents significant challenges. Because cuffless BP estimation models (Supplementary Note 3) rely on physiological surrogates rather than direct pressure, they involve a multitude of parameters. These parameters exhibit high inter-subject variability and can also fluctuate situationally within the same individual. While calibration establishes these parameters using direct reference pressure measurements, a "calibration-free" approach must estimate them indirectly, introducing additional complexity and potential error.

In this work, we opted for an established, validated model. Given that achieving the evolving clinical standardization for cuffless BP monitoring remains an open challenge for the field, our priority was to first reach this critical milestone using a robust and reliable methodology. Our system collects physiological data with fidelity that matches or exceeds systems attempting calibration-free approaches, most notably through our highly accurate measurement of the full diameter waveform.

A calibration-free system remains an important long-term goal. As we have now expanded upon in the Discussion, several recent works have focused on building such frameworks[16,17]. We believe that once the primary milestone of standardized accuracy is confidently achieved, the high-quality, comprehensive data captured by our optoacoustic system will provide an excellent foundation for relaxing calibration requirements in future iterations.

2-8

Finally, although the sensing concept is compelling, the current implementation relies on bulky laboratory hardware (tunable laser, fiber interferometer, and bench-top electronics), and the gap to a truly wearable system remains large. The authors can discuss the challenges to develop a fully wearable portable system and possible strategies to address these challenges.

Indeed, the current prototype system is still bulky and based on expensive components, as is often the case with first-generation systems. We now discuss in more detail in the Discussion how the system's complexity could be massively reduced, potentially to the level of a wearable device. Importantly, our proposed strategy for reducing complexity relies on components and capabilities which have already been developed in previous works, and thus it may be achieved by pure engineering effort. In the following, we briefly discuss the main components in our system and how they could be replaced:

- Optoacoustic laser: in our setup we used a preexisting InnoLas OPO source that can deliver up to 30 mJ pulses with durations of 7 ns at arbitrary wavelengths in the range of 680 - 980 nm. In all three parameters, the performance of this laser was superfluous, but it did simply the system development considering the overall uncertainty in overall performance. In practice, we used pulses of only 10 mJ (4.9 mJ at the skin) and a single wavelength of 800 nm, whereas the pulse duration could have been increased to 25 ns without affecting the bandwidth of the signal (see our reply to Comment 1-4). Additionally, our analysis shows that the intrinsic noise of the sensor was approximately half of the background signal from the tissue (Methods), and therefore similar results could have been obtained with lower pulse energies, *e.g.,* 3 mJ. Such energy can be readily achieved with pulsed laser diodes, which are commercially available (e.g., Lumibird). Furthermore, since such laser diodes can operate at a much higher repetition rate than the OPO source used in this work, lower energies may be used, enabling cheaper sources, with signal averaging used to partially compensate for the loss of signal. An additional option for low-cost sources is VCSELs, which were used in Ref.[2].
- Readout system: The interferometric system currently consists of a fiber-based Mach-Zehnder interferometer, photodiodes, and a tunable CW laser. All these components can be miniaturized today on a single silicon-photonics chip without sacrificing performance. Typical examples may be found in Ref [4].
- Sampling electronics: since we need to sample only a single channel and do not require to generate a voltage pulse, the electronics are considerably simplified. For example, typical chips for analog to digital conversion from TI (e.g. ADC3563) have typical dimensions of 5 × 5 mm and cost less than 10$.
- Wearable probe: the wearable probe in our work had dimensions with cm × cm footprint to enable ±1 cm positioning robustness. While ultrasound patches, based on dense arrays, would require the same footprint, there is a major difference. In the case of ultrasound patches, the entire patch needs to be in contact with the skin to enable all the individual elements to operate. In our case, only a single element, which can be sub-millimeter in width, needs to be in contact with the tissue, whereas the illumination can be applied without any contact, facilitating simpler designs. For example, the illumination may be applied by a wearable, loose contact, wrist band or watch.
- System simplification via increased sensitivity: We now discuss that further improvement to the sensitivity of SPADE may be achieved, enabling further reduction in the optoacoustic illumination power, and thus simpler miniaturization of the source. Specifically, since our main noise source is photon shot noise (Supplementary Fig. 13), which is independent of the resonator's Q-factor, further improvement in sensitivity can be achieved by using higher Q-factors, which will lead to stronger signals. As mentioned in Comment 2-2, Q-factors of 10^6, 5 times higher than ours, are common in silicon photonics.

References

1. Zhou, S. *et al.* Clinical validation of a wearable ultrasound sensor of blood pressure. *Nat. Biomed. Eng* **9**, 865–881 (2024).
2. Gao, X. *et al.* A photoacoustic patch for three-dimensional imaging of hemoglobin and core temperature. *Nat Commun* **13**, 7757 (2022).
3. Konradl, J. *et al.* High-power compact VCSEL subsystem as game-changer for photoacoustic medical imaging and sensing. in *Vertical-Cavity Surface-Emitting Lasers XXIX* (eds Choquette, K. D. & Graham, L. A.) 22 (SPIE, San Francisco, United States, 2025). doi:10.1117/12.3042742.
4. Deng, H. *et al.* Single-Chip Silicon Photonic Engine for Analog Optical and Microwave Signals Processing. Preprint at https://doi.org/10.21203/rs.3.rs-4539048/v1 (2024).
5. Boas, D. A. & Yodh, A. G. Spatially varying dynamical properties of turbid media probed with diffusing temporal light correlation. *J. Opt. Soc. Am. A* **14**, 192 (1997).
6. Nagli, M. *et al.* Silicon photonic acoustic detector (SPADE) using a silicon nitride microring resonator. *Photoacoustics* **32**, 100527 (2023).
7. Wang, L. V. Photoacoustic Imaging and Spectroscopy.
8. Hysi, E., Moore, M. J., Strohm, E. M. & Kolios, M. C. A tutorial in photoacoustic microscopy and tomography signal processing methods. *Journal of Applied Physics* **129**, 141102 (2021).
9. Guelen, I. *et al.* Validation of brachial artery pressure reconstruction from finger arterial pressure. *Journal of Hypertension* **26**, (2008).
10. Kireev, D. *et al.* Continuous cuffless monitoring of arterial blood pressure via graphene bioimpedance tattoos. *Nat. Nanotechnol.* **17**, 864–870 (2022).
11. Fang, J. *et al.* Perspectives of Global Standards on Validation of Blood Pressure Measuring Devices. *IEEE Trans. Biomed. Eng.* 1–15 (2025) doi:10.1109/TBME.2025.3587411.
12. Lin, M. *et al.* A fully integrated wearable ultrasound system to monitor deep tissues in moving subjects. *Nat Biotechnol* **42**, 448–457 (2024).
13. Yuan, J. *et al.* Skin-adaptive focused flexible micromachined ultrasound transducers for wearable cardiovascular health monitoring. *Science AdvAnceS* (2025).
14. Sinclair, M. *et al.* 1.4 million Q factor $Si_3 N_4$ micro-ring resonator at 780 nm wavelength for chip-scale atomic systems. *Opt. Express* **28**, 4010 (2020).
15. Maslov, K., Stoica, G. & Wang, L. V. In vivo dark-field reflection-mode photoacoustic microscopy. *Opt. Lett.* **30**, 625–627 (2005).
16. Nabeel, P. M., Jayaraj, J., Srinivasa, K., Mohanasankar, S. & Chenniappan, M. Bi-Modal Arterial Compliance Probe for Calibration-Free Cuffless Blood Pressure Estimation. *IEEE Trans. Biomed. Eng.* **65**, 2392–2404 (2018).
17. Tang, J. *et al.* Calibration-free estimation algorithm for cuffless continuous blood pressure measurement based on ultrasonic devices. *NSO* 20240014 (2024) doi:10.1360/nso/20240014.

Reviewer 1

The revised manuscript is substantially improved, and the authors have made strong efforts to address the technical concerns raised in the previous review, which are greatly appreciated. It is recommended that the remaining concerns be addressed before this manuscript sees publication.

We appreciate the Reviewer's constructive feedback. The manuscript was modified according to the Reviewer's comments. In the following is a point-by-point response addressing each remark.

1-1

The bandwidth concern is now largely resolved. Supplementary Figs. 40–42 directly address the concern that tissue attenuation would reduce the effective bandwidth to well below the reported 125 MHz of the SPADE detector. The authors appropriately acknowledge that the intrinsic 125 MHz detector bandwidth is not the effective in vivo bandwidth at the radial-artery depth, and Supplementary Fig. 40 is helpful in showing that the diameter-estimation error remains small above approximately 6 MHz. The manuscript should nevertheless consistently refer to 125 MHz as the intrinsic detector bandwidth, rather than implying that this is the effective in vivo bandwidth. Also, this necessarily raises the question of whether the high bandwidth of 125 MHz is necessary, as shown in Supplementary Fig. 40, a much smaller bandwidth is sufficient.

We thank the reviewer for this observation. We agree that parts of the previous text, if read separately, could give the false impression that 125 MHz reflects the *in vivo* signal bandwidth. We have revised the manuscript to consistently refer to 125 MHz as the intrinsic SPADE bandwidth, measured with a dedicated optoacoustic source of 1 ns pulse duration, and to state explicitly (in both the Introduction and Discussion) that this significantly exceeds the bandwidth of the signals from the radial artery.

Regarding whether 125 MHz is necessary: we have updated Supplementary Table 2 to separate the bandwidth limitations into Tx* (governed by the excitation laser pulse width) and Rx (governed by the sensor), with the total effective bandwidth and axial resolution determined by whichever is the limiting factor. This analysis shows that SPADE's 125 MHz shifts the bottleneck away from the detector and onto the laser and tissue, allowing the tissue's intrinsic bandwidth potential to be fully realized with a sufficiently short-pulse source and with no limitations imposed by the SPADE. Even though the entire 125 MHz of SPADE is not necessary, we consider the excess in bandwidth to be an important property to report for three reasons: (**1**) Common wearable piezoelectric transducers operate at bandwidths too low to fully capture the optoacoustic signal from the radial artery in all cases, making them a limiting factor in competing patch technologies. (**2**) The 125 MHz bandwidth is an inherent property of SPADE that did not come at the expense of other performance metrics. (**3**) There would be little practical benefit from reducing it, except possibly lower-cost electronics.

1-2

The added analysis of the leading/trailing discontinuity ratio and the discussion of dark-field illumination provide a plausible explanation for why the posterior boundary remains detectable. However, because the method is intended for continuous monitoring, the authors should state explicitly whether any beats or measurements failed automatic boundary tracking, whether any events were rejected by the processing algorithm, and whether any manual correction was applied. This information would directly address the concern that minimum-based tracking may occasionally become unstable in real-time or long-term operation. The automated processing criteria should also be sufficiently clear to enable readers to understand how the reported BP accuracy metrics were obtained.

We thank the reviewer for this important clarification request. We address each point in turn.

Failure rate and boundary tracking. In the results reported in this study, no events were rejected. Because the analysis was performed off-line, every instance in which the automatic detector failed was corrected by manually redirecting it to the correct region. The overall failure rate of the envelope detection stage was approximately 5%, and failures occurred with equal prevalence at the anterior and posterior boundaries - the trailing (posterior) boundary was not disproportionately affected. We therefore note that the question of envelope detection reliability is decoupled from the question of leading/trailing discontinuity ratio.

Two-stage algorithm and failure modes. The boundary localization algorithm operates in two sequential stages. The first stage identifies the region containing the leading and trailing edges to within 50 ns; the second stage extracts the precise inflection and minimum points within those regions. The success rate of the second stage was 100% and had no manual intervention. Failures were confined exclusively to the first stage and were not attributable to reduced SNR - they arose from the boundary-tracking software mistakenly latching onto an acoustic reflection or background source, i.e., a reduced signal-to-background rather than signal-to-noise ratio.

Path to fully automated real-time operation. We acknowledge that continuous monitoring will ultimately require a fully automated, intervention-free first stage. We note, however, that the visual unambiguity of the true boundary makes this a straightforward engineering task, particularly with a learning-based approach trained on a modest labeled dataset. Given the rapid pace of development in this area, we consider it more prudent to defer this effort to when full real-time operation is needed, at which point we anticipate it can be accomplished with minimal effort. Data from our planned large-scale clinical trial will be used to train such a detector.

The relevant section of the Methods has been updated to explicitly state the two-stage nature of the algorithm, the 5% first-stage failure rate, the absence of any rejected events, the nature and equal prevalence of anterior/posterior failures, and the role of manual correction in the off-line analysis.

1-3

The optical-fluence characterization is improved as well. These additions address the concern that the posterior side of the artery may be insufficiently illuminated. The reviewer recommends that the authors, if possible, add a more explicit quantitative statement of the fluence, or the anterior/posterior signal ratio, at the relevant anterior and posterior arterial depths. This would make the response to the 4–6 mm depth issue clearer without requiring readers to infer values from the figure.

We thank the reviewer for this suggestion; two additions have been made to address it. First, the device characterization section now explicitly states the simulated fluence at the anterior and posterior wall depth ranges, indicating adequate illumination across the arterial cross-section. Second, we now explicitly quantify the anterior-to-posterior signal amplitude ratio from the *in vivo* measurements, confirming comparable signal generation at both boundaries.

1-4

The clinical-validation language should be tempered. The additional NOVA validation against a brachial cuff is useful, and the revised Bland-Altman plot now appropriately separates SBP and DBP, and the apparent concern about a large SBP mean bias has also been resolved. However, the study still includes only two subjects, uses subject-specific calibration, and compares the optoacoustic estimates with a NOVA-derived radial reference waveform rather than with an invasive radial arterial line or a full sphygmomanometer-based validation protocol. The data supports a pilot feasibility comparison, not full clinical validation. The statements “within clinically acceptable standards” and “corresponds to a grade A device” should be explicitly qualified or removed. A more accurate formulation would be that the two-subject pilot study achieved low MAD relative to a calibrated NOVA-derived radial reference, while full compliance with cuffless BP validation standards remains to be demonstrated in a larger clinical study. The manuscript should also avoid implying that the 120,000 sampled diameter-pressure pairs are statistically independent validation samples. The primary performance metrics appear to be based on 120 ten-second measurements, and this should be stated consistently.

We thank the reviewer for these clarifications. The manuscript and Supplementary Information have been revised accordingly: the "grade A" and "clinically acceptable" language has been replaced with a contextual comparison to leading wearable BP patches of similar participant numbers, the 120-measurement basis of the primary performance metrics is now stated consistently, and the Discussion explicitly states that full compliance with cuffless BP validation standards remains to be demonstrated in a larger study. We note that invoking standard grading metrics in the context of pilot studies - without fully meeting their requirements - is common practice in leading published literature[1]. The only work to our knowledge that has rigorously met these requirements is the wearable ultrasound BP study by Zhou et al.[2], and we agree that this sets the appropriate bar. We have followed the Reviewer's guidance and adopted more conservative language throughout.

1. Kireev, D. *et al*. Continuous cuffless monitoring of arterial blood pressure via graphene bioimpedance tattoos. *Nat. Nanotechnol.* **17**, 864–870 (2022).
2. Zhou, S. *et al*. Clinical validation of a wearable ultrasound sensor of blood pressure. *Nat. Biomed. Eng* **9**, 865–881 (2024).

1-5

The discussion of prior wearable ultrasound systems should also be more balanced. The authors now appropriately acknowledge that wearable ultrasound BP monitors and fully integrated wearable ultrasound systems have been reported. However, the revised manuscript should avoid implying that pulse-echo ultrasound is fundamentally incompatible with wearable BP monitoring. The authors' data support a narrower and more defensible claim: current pulse-echo implementations face a trade-off between placement robustness and system complexity, particularly for small superficial arteries such as the radial artery near the wrist.

We thank the reviewer for this suggestion and agree that the original framing was overstated. The manuscript and Supplementary Information have been revised to avoid implying a fundamental incompatibility between pulse-echo ultrasound and wearable BP monitoring. The claim has been narrowed to smaller arteries specifically, and the Discussion now explicitly acknowledges the currently greater maturity and conformability of piezoelectric patch technology before arguing that the optoacoustic approach carries an inherent advantage for BP monitoring, owing to its superior vessel-to-tissue contrast and enhanced placement insensitivity.
